# Leisure Activities: A Narrative Review of Established Evidence and New Perspectives on the Cognitive Reserve Framework in Healthy Populations Across the Adult Lifespan

**DOI:** 10.3390/brainsci16101031

**Published:** 2026-09-26

**Authors:** Martina Rizzuti, Erica Berretta, Valeria Gosti, Chiara Baglioni, Laura Serra, Francesca Gelfo

**Affiliations:** 1 Department of Human Sciences, Università degli Studi Guglielmo Marconi, 00193 Rome, Italy; m.rizzuti@unimarconi.it (M.R.); e.berretta@unimarconi.it (E.B.); v.gosti@unimarconi.it (V.G.); c.baglioni@unimarconi.it (C.B.); l.serra@unimarconi.it (L.S.); 2 LIFE—Istituto di Ricerca e Cura—Santa Lucia IRCCS, 00179 Rome, Italy; 3 Department of Psychiatry and Psychotherapy, Medical Center—University of Freiburg, Faculty of Medicine, University of Freiburg, 79104 Freiburg, Germany

**Keywords:** cognitive reserve, leisure activities, brain resilience, healthy subjects

## Abstract

**Highlights:**

**What are the main findings?**

- Across adulthood, engagement in cognitive, social, and physical leisure activities is associated with better cognitive performance and emotional well-being, with cognitive activities more closely linked to cognitive gains and social/physical activities more closely linked to combined cognitive–emotional benefits.
- Neurophysiological and neurobiological markers (e.g., BDNF, cortisol, functional connectivity) reported alongside these benefits should be interpreted as underlying mechanisms that may accompany, but do not by themselves demonstrate, an increase in cognitive reserve, which was not directly measured in any of the studies reviewed.

**What are the implications of the main findings?**

- Cognitive, social, and physical leisure activities represent accessible and modifiable behavioral targets that could be promoted across adulthood to support healthy cognitive aging, thereby potentially contributing to the construction of cognitive reserve over the lifespan.
- Because most of the available evidence is observational or short-term, longitudinal and experimental studies are needed to clarify causal pathways and to disentangle genuine reserve-building effects from confounders such as education and socioeconomic status.

**Abstract:**

Background/Objectives: The concept of Cognitive Reserve (CR) has assumed a central role in research on the prevention of cognitive decline. The term refers to the brain’s capacity to manage age-related changes or pathologies by enhancing neural efficiency and utilizing alternative networks or more efficient cognitive strategies. In human subjects, CR is commonly estimated through indicators such as educational attainment, occupational complexity, and involvement in leisure activities. Leisure activities have been identified as a significant behavioral indicator and can be classified into three main subcomponents: cognitive, physical, and social activities. This narrative review aims to summarize how leisure activities provide cognitive and/or emotional benefits and how these activities help build CR and brain resilience. Methods: Given the conceptual and methodological heterogeneity of this literature, a narrative review design was adopted for an integrative synthesis across cognitive, social, and physical domains in healthy individuals aged 18 and older. Selected studies’ primary outcomes were cognitive and/or emotional effects, with neurophysiological effects as secondary outcomes, a prerequisite for inclusion. Results: Across adulthood, participation in cognitive, social, and physical leisure activities was consistently associated with better cognitive performance and emotional well-being, with cognitive activities more closely linked to cognitive gains and social and physical activities more closely linked to combined benefits; however, evidence is observational or short-term, limiting causal inference. Conclusions: Leisure activities are consistently associated with cognitive and emotional well-being in healthy adults and may support CR development, although longitudinal and intervention research is needed to clarify causal pathways and confounders such as education and socioeconomic status.

## 1. Introduction

### 1.1. The Concept of Reserve

The term “reserve” refers to an individual’s capacity to sustain a particular function in the presence of damage or disease [1]. A substantial body of literature has been devoted to this topic by various researchers [1,2,3,4,5,6,7], who distinguish among: (1) “Brain Reserve” (BR), denoting the “neurobiological capital” of the brain’s physical structure, such as brain volume, number of neurons, and synaptic density, which is commonly operationalized through proxies like head circumference, intracranial volume, and cortical thickness; (2) “Cognitive Reserve” (CR), referring to the brain’s adaptive capacity to cope with age- or pathology-related changes by exploiting pre-existing cognitive strategies more efficiently and/or by recruiting alternative cognitive processes; (3) “Neural Reserve” (NR), which refers to the intrinsic operational efficiency of brain networks to optimize information processing.

In recent years, these three concepts have been complemented by the notions of Neural Compensation (NC) and Brain Maintenance (BM). NC is defined as the process by which individuals who have suffered brain damage utilise alternative brain structures or circuitry when the neural function of a primary network is unable to maintain sufficient capacity to cope with the environmental demands. This concept is related to the concept of CR [7,8]. BM, complementary to CR, denotes the preservation of brain integrity over time by slowing or preventing age-related neural changes and pathology driven by genetics and lifestyle factors [1,2,3,4]. It is important to acknowledge that, despite these conceptual distinctions, the term “cognitive reserve” is used in the literature as an umbrella term that refers to the broader complexity of these constructs related to reserve.

### 1.2. Cognitive Reserve Proxies

CR is accumulated through enriching life experiences [3,5]. Such experiences have been demonstrated to increase brain plasticity and resistance to cell death [3,9]. It is evident that a number of factors contribute to the development of CR, including educational level (manifested in years of schooling), professional complexity, and leisure activities. These factors are recognised as proxy measures of CR [3,5]. However, as outlined in this study [5], additional factors that promote CR have been described. These include bilingualism [10], continuing education [11], and a healthy lifestyle that includes a Mediterranean diet, regular physical activity, responsible alcohol consumption, limiting caffeine and nicotine, avoiding overeating, and maintaining good sleep habits [12]. From this perspective, CR should not be understood as a static construct, but as the result of a process that develops over time.

#### Assessment Tools for Cognitive Reserve

In order to assess an approximate estimate of CR, several questionnaires have been developed; Table 1 summarizes some of the questionnaires that include different factors that can be considered as reserve proxies (such as educational and occupational attainments, and different leisure activities).

**Table 1:** Tools to estimate Cognitive Reserve.

Instrument	Domains/Content Assessed	Population/Setting
Cognitive Reserve Index Questionnaire (CRIq) [13] and its short version, the s-CRIq [14] (short CRIq version)	Education, occupational activity, and leisure activity from age 18 onward	Clinical and research setting
Global Enrichment Index (GE-I) [15]	Education, occupational complexity (9 ISTAT categories), and cognitive, social, and physical activities across three age groups (20–40, 40–65, ≥65 years)	Alzheimer’s disease research (original validation in an Italian sample)
Cognitive Reserve Questionnaire (CRQ) [16]	Personal and parental education, training, and occupation	Clinical dementia setting
Lifetime of Experiences Questionnaire (LEQ) [17]	Educational, occupational, and cognitive activities at different stages of life	Originally validated in adults aged ≥ 65 years
Modified Cognitive Reserve Scale (mCRS) [18]	Hobbies, information-seeking behaviours, and social relationships (education and occupation not included)	General adult sample
Cognitive Reserve Assessment Scale in Health (CRASH) [19]	Education, occupation, intellectual and recreational activities	Developed for populations with severe mental illness

### 1.3. The Role of Leisure Activities in Modulating Cognitive Reserve

Leisure activities have been proposed as an important predictor of well-being and possible factors contributing to CR [20]. Indeed, participation in leisure activities has been demonstrated to be correlated with enhanced mental and physical well-being [21]. These activities are typically characterised as voluntary, non-compulsory, non-work-related, and based on personal pleasure and interests [22,23]. In young people, leisure activities have been shown to positively influence identity by satisfying their needs for belonging and self-actualisation, developing creativity and self-confidence, and acquiring new skills [24]. However, these activities have also been examined in the context of aging. Indeed, Jopp and Hertzog [25] describe how engagement in stimulating recreational activities has been linked to higher levels of well-being and lower levels of depression in old age, good physical health, independence in daily living activities, and good cognitive functioning.

The classification of leisure activities through the scientific literature has been subject to varied approaches, reflecting the diverse range of experiences that these activities embody. Kleiber et al. [26] identify leisure activities in three types: relaxed (e.g., socialising, reading, listening to music), serious (e.g., sports, arts), and unclassified (e.g., thinking, resting). Everard et al. [27] propose a classification system based on the level of physical and mental commitment required by different activities. The activities are divided into two categories: high-demand activities, such as sport and gardening, and low-demand activities, such as sewing or reading. Furthermore, instrumental activities—such as shopping—are introduced, which, although less demanding, still constitute a significant part of leisure time [27]. Similarly, Holder et al. [22] propose a distinction between active activities, such as playing sports, and passive activities, such as watching television or listening to music. Passmore et al. [28] focus on the purpose of activities, distinguishing between leisure time in the traditional sense, which includes passive and solitary activities, and leisure time for fulfilment, which includes activities that pose challenges and opportunities for personal growth. An alternative approach is that of Ellis et al. [29], who emphasise the *expressive aspect* of recreational activities, introducing categories such as transitional leisure, linked to artistic practices, hobbies, music, and sport, and fantasy-related leisure, such as reading or watching television programmes. Finally, Stephan et al. [30], have proposed a higher-order categorisation system that distinguishes between physical, social and mental activities.

The tripartite classification of cognitive, social, and physical activities adopted in the present narrative review serves as an organizational framework rather than considering these as a set of mutually exclusive categories. Leisure activities often involve overlapping cognitive, physical, and social components [31,32]. For the purposes of this review, each activity is discussed under the domain it most clearly exemplifies. This organisational choice does not imply that the reported outcomes can be attributed exclusively to that domain.

In summary, participation in leisure activities encompassing the cognitive, social, and physical domains has been proposed as a plausible contributor to CR and, more broadly, to healthier aging and resilience to neurodegenerative disease.

While reserves are defined as a whole in neurobiological terms, CR cannot be observed directly. The “Reserve, Resilience and Protective Factors Professional Interest Area of the Alzheimer’s Association” workgroup [33] has proposed that CR reflects the flexibility with which cognitive processes are able to adapt; this explains why two individuals with a comparable degree of brain aging or pathology may exhibit very different levels of cognitive or functional impairment [1]. Consistent with this, CR is typically inferred from behavioural outcomes: an individual is considered to show high CR when his/her cognitive functioning is better than expected given the degree of age- or pathology-related brain changes [2,3].

#### 1.3.1. Assessment Tools for Leisure Activities

There are numerous questionnaires used to assess specific leisure activities. Table 2 summarises some of the questionnaires assessing cognitive, social or physical leisure activities.

**Table 2:** Questionnaires used to assess leisure activity engagement.

Instrument	Domains/Content Assessed	Population/Notes
Victoria Longitudinal Study-Activity Lifestyle Questionnaire (VLS-ALQ) [34], updated Self-Report Activity Questionnaire [25]	Cognitive, social and physical activity frequency; the updated version further distinguishes private, public, and religious social activities	Young to older adults
Paragraphs About Leisure (PAL) [35]	Need-gratification across art, music, sports, volunteering, education, and culture	Mainly university students
Cognitive Leisure Activity Scale (CLAS) [36]	Passive activities (such as watching TV or listening to music), gaming (such as solving puzzles or playing chess), social (such as socialising with friends), artistic (such as painting and drawing), and physical exercise	Adults aged 38–98 years
Florida Cognitive Activities Scale (FCAS) [37]	Degree and intensity of cognitive and social engagement in daily activities	General adult samples
Whitehall II Study (WHII) questionnaire [38]	13 past-year leisure activities (e.g., volunteering, cultural events, social visits, hobbies), weighted by cognitive/social engagement level	Adult occupational cohort
Godin-Shephard Leisure-Time Physical Activity Questionnaire (GSLTPAQ) [39]	Leisure Score Index (LSI) derived from weekly light/moderate/vigorous activity frequency; classifies sufficient vs. insufficient activity against international guidelines	General adult samples; classification validity more recently confirmed against objective fitness and body-composition markers [40]
Quick Physical Activity Rating (QPAR) [41]	Passive activities, walking, hobbies, exercise and housework, distinguishing between light, moderate and vigorous activities	Adults with an average age of around 75
Leisure activities of adolescent schoolchildren [42]	Outdoor activities (e.g., sailing or swimming), entertainment (e.g., going to concerts or the cinema), sports (e.g., rugby), social activities (e.g., dinners with friends or family visits), and hobbies (e.g., listening to music or playing billiards)	Schoolchildren; adolescent population

#### 1.3.2. Multidimensional Benefits of Leisure Activities

The model developed by Fancourt et al. [23] posits that leisure activities can engender multilevel effects involving psychological, biological, social, and behavioural dimensions. At the individual level, they contribute to emotional well-being, strengthen resilience, and build a sense of self. Furthermore, they have the capacity to stimulate physiological mechanisms involving various systems, including the neuroendocrine, immune, central nervous, and cardiometabolic systems, thereby producing integrated and positive responses for the body. From a sociological perspective, recreational activities have been shown to facilitate the expansion of relational networks, the strengthening of existing bonds, and the development of healthy habits among individuals within their respective communities. At the group level, collective engagement in these activities has been demonstrated to have a number of notable consequences. These include the strengthening of cohesion, the fostering of the construction of a common identity, and the influence of social attitudes and cooperative behaviours, through repeated shared participations, which fosters mutual trust and reinforced group norms [23].

According to the CR framework [1], the majority of previous reviews focused on pathological populations [43,44,45,46]. Conversely, when investigating healthy subjects, most studies have assessed only specific activities [47] or, even when considering a broader spectrum, focused mainly on healthy older adults and on traditional activities (such as writing, reading, playing cards, visiting relatives, going to the cinema, watching TV, and engaging in individual sports) [48]. The present review aims to extend current knowledge by focusing specifically on healthy participants across the adult lifespan and by considering a wide spectrum of cognitive, social, and physical leisure activities beyond those traditionally studied. Moreover, this perspective allows for the consideration of leisure engagement not solely in terms of cognitive outcomes, but also in relation to emotional, neurophysiological, and neurobiological findings, which may offer complementary insights into potential contribution to CR. This broader perspective may facilitate the identification of areas where the evidence is heterogeneous or insufficiently characterized, thereby identifying environmental stimulating factors that can be leveraged to promote cognitive functioning and well-being in healthy individuals.

## 2. Materials and Methods

### 2.1. Review Design

The present review adopted a narrative approach for three principal reasons: the aim was to integrate a literature that is conceptually and methodologically heterogeneous (i), encompassing cognitive, social, and physical leisure domains (ii), and multiple outcome types (cognitive, emotional, and neurophysiological) (iii) within a unified conceptual framework [49,50]. In order to ascertain the methodological transparency, this study adheres to the SANRA quality criteria for narrative reviews [51], clarifying the literature search strategy, the eligibility criteria applied, and the data synthesis.

### 2.2. Search Strategy and Study Identification

For this narrative review, relevant studies were identified through searches conducted in the PubMed and Scopus databases, with no time restrictions, up to January 2026.

The search strategy was developed to explore the relationship between cognitive reserve and involvement in leisure activities. The following terms were combined for the purposes of this study: cognitive reserve, cognitive activities (e.g., “reading”, “board games”), social engagement (e.g., “social support”, “social participation”, “social interaction”), and physical activities (e.g., “sports”, “walking”, “yoga”). The search was refined using the Boolean operators AND and OR to include a wide range of activities and cognitive and emotional outcomes. According to the SANRA quality criteria [51], the combinations of terms were used to identify relevant articles exploring how cognitive, social, and physical leisure activities may influence CR and mental health in healthy adults. Relevant literature was identified through an iterative citation-searching process. A manual screening of the reference lists of retrieved and key articles was conducted to identify additional relevant studies through the process of backward citation searching. Regardless of the route through which the study was identified, each article considered for inclusion was assessed against the eligibility criteria described below.

### 2.3. Eligibility Criteria and Study Selection

The studies included in the review had to meet the following criteria: (1) the study population was required to comprise healthy individuals aged 18 years or older; (2) the variables under consideration were required to relate to cognitive, physical, and social activities undertaken during leisure time, and therefore could not be activities carried out for or during work; (3) the studies were required to include cognitive and/or emotional outcomes associated with cognitive, social, or physical leisure activities, with any neurophysiological or neurobiological effects considered as secondary outcomes. Because CR is a latent construct that cannot be directly observed, cognitive and emotional outcomes were considered relevant to the study of processes potentially related to cognitive reserve, rather than as direct measures of the construct. The activities encompassed those categorised as leisure activities, along with those subjected to experimental scrutiny to evaluate their impact on well-being and their potential relevance to CR-related processes. The classification of studies was determined by the application of controlled conditions or specific interventions on activities, thereby categorising them as experimental studies. Conversely, if the analysis focused on existing activities or spontaneous behaviours, they were designated as observational studies.

The following criteria were applied to the exclusion of studies: (1) reviews or meta-analyses; (2) conference proceedings; (3) animal studies.

### 2.4. Data Extraction

Findings were extracted narratively within each of the three activity domains (cognitive, social, and physical), considering study design, typology, frequency, and duration of the intervention, age of the subjects, sex distribution, and outcome types.

## 3. Results

The 83 studies included in the present review are heterogeneous for methodological design, demographical features of the sample, and typology/frequency/duration of the intervention when considered. Details are reported in Table 3, Table 4 and Table 5, and in the specific sections. Results are synthesized according to the three main domains of interest (i.e., cognitive, social, and physical leisure activities). Moreover, within each domain of interest the results are pooled for the specific activity (such as reading, writing, painting, etc.).

### 3.1. The Effects of Cognitive Leisure Activities

Several studies have reported cognitive and emotional effects associated with engaging in cognitively stimulating leisure activities. Within the context of leisure-time activities, cognitively stimulating activities comprise a heterogeneous group of practices that differ in terms of cognitive load, emotional involvement, and degree of active participation. A plethora of activities have been studied in terms of their potential to be defined as cognitively stimulating. Such activities include: reading; writing; gaming and puzzle-based activities; learning a new language; attending the theatre, cinema, or concert; listening to music and playing a musical instrument. For clarity, the available evidence is presented by grouping studies according to the primary type of cognitive engagement involved.

We included 25 studies focused on cognitive leisure activities. Of these, 36% (9/25) were observational studies (incorporating both cross-sectional and longitudinal designs), while the remaining 64% were experimental (including randomized controlled trials and non-randomized studies). Among the experimental studies, 69% assessed outcomes immediately post-intervention (acute outcomes), whereas 31% evaluated outcomes in both the short and long term (mixed acute/long-term) (see Table 3 for further details).

#### 3.1.1. Reading

Among cognitive activities, reading has received considerable attention due to its accessibility and its potential role in supporting CR throughout life. This has been attributed to its classification as an active, novel, and receptive form of intellectual engagement, which, unlike passive activities such as watching television, has been shown to moderate the effect of age on semantic memory [52]. Two studies involving reading activities were included. Wang et al. [53] conducted a population study of cognitively healthy adults (age range: 40–100 years), comparing regular readers (defined as individuals who read on average, at least one book for a month over a period of at least one year) with non-readers. Participants demonstrating regular reading habits exhibited significantly higher cognitive performance in several domains, including global cognition, attention, memory, language, visuospatial skills, and executive functions (Table 3). The findings of logistic and linear regression analyses indicated that the association between reading and cognitive performance was independent of age and sex. However, to assess whether reading activities have a compensatory effect for low education, participants with low education (≤12 years) who committed to reading more books were able to achieve similar or even superior cognitive scores in tests involving global cognition, memory, and attention, when compared with those with high education. No significant differences in cortical thickness or hippocampal volume were observed between readers and non-readers in magnetic resonance imaging analyses (Figure 1); a positive correlation between years of education and hippocampal volume was identified (Figure 1). A further study [54], conducted in China on a large group of elderly people (age range: 83–93 years), found that participants who regularly engaged in mentally stimulating activities, such as reading books or newspapers, watching television or listening to the radio, and playing cards or mahjong, were less likely to suffer from cognitive decline. In particular, it was observed that individuals who engaged in reading on a regular basis were less prone to experience cognitive decline compared to those who never read (Table 3).

#### 3.1.2. Expressive Writing

In addition, experimental studies have examined the cognitive and emotional effects of expressive writing as a form of cognitively and emotionally engaging activity. In two different six-month-long experiments involving university students (age range: 18–19 years), Klein and Boals [55] examined the effects of expressive writing on working memory (WM). In the first experiment, students were tasked for 2 weeks with articulating their reflections and sentiments concerning the transition to university life. A significant difference in working memory scores was observed in the experimental group after six weeks (Table 3). This difference was not observed in the control group, which consisted of students assigned to write about a trivial topic. In the second experiment, students who engaged in expressive writing about a negative personal experience demonstrated larger WM gains and a reduction in intrusive thoughts compared to those who wrote about a positive experience or a neutral topic. In their study, Cheung and Ng [56] examined the impact of expressive writing on cognitive reappraisal over time by comparing expressive writing with conventional writing and the absence of writing. For a period of three days, participants (age range: 18–25 years) in the expressive writing condition were asked to write about their deepest thoughts and emotions regarding a traumatic experience. The findings of the study indicated that expressive writing was associated with enhancements in adaptive emotional regulation strategies, including cognitive reappraisal, as well as favorable outcomes in terms of well-being, such as life satisfaction and overall health, in a sample of young adults (Table 3).

#### 3.1.3. Gaming and Puzzle-Based Activities

Research has shown that gaming can be considered a form of mentally stimulating leisure activity, involving high levels of active engagement. This includes an experimental crossover study by Urwyler et al. [57] conducted on middle-aged and elderly adults (age range: 45–75 years). In this study, the participants alternated between a control condition involving reading newspapers and a digital cognitive training intervention based on puzzle games. The intervention was delivered via tablet and monitored using an artificial intelligence-based recording system. The results demonstrated a substantial enhancement in visual scanning performance and a significant improvement in the visuospatial-executive domain in assessments following the gaming intervention. In a cross-sectional study [58], the association between gaming activities and cognitive functions in young and middle-aged adults (age range: 18–59 years) was analyzed. Controlling for age and education, time spent playing video games significantly predicted performance in several cognitive domains, including fluid intelligence, mental flexibility, working memory, planning, and visuospatial processing speed (Table 3). Overall, the results indicate that time spent gaming significantly predicts cognitive performance, with video games having a greater influence than board games. In particular, fluid intelligence abilities increase with increased time spent playing video games, especially beyond 10 h per week. Planning skills and verbal skills also show a positive relationship with time spent playing video games, although this association tends to stabilize between 10 and 20 h of play per week. In contrast, visuospatial processing speed improves with increasing play time up to about 20 h per week, then declines in the most avid players. Moreover, the study by Kaynak et al. [59] demonstrated that the integration of crossword puzzles into distance learning for nursing students resulted in a significant enhancement in cognitive performance, particularly in terms of problem-solving and decision-making skills (Table 3).

#### 3.1.4. Linguistic Activities and Bilingualism

Beyond reading, writing, and gaming, language activities represent a form of cognitive stimulation. Recent experimental studies have examined the role of linguistic activities, such as learning a new language or using bilingualism, as forms of cognitive stimulation. Bubbico et al. [60] conducted an experimental study on middle-aged and elderly Italian adults (age range: 59–79 years) to investigate the effects of learning a new language (English) on cognitive functions. The sample comprised 26 participants, who were assigned to either an experimental group or a control group. The linguistic intervention entailed structured foreign language learning, while the control group did not receive any linguistic training. However, the control group completed pre- and post-assessments, as well as a neuropsychological battery both before and after the study period. To ensure that there were no changes in their lifestyle, control participants were contacted via monthly phone calls throughout the 4-month study. Both groups also underwent resting-state functional Magnetic Resonance Imaging (fMRI) scans at the beginning and end of the study period. The results demonstrated a marked global cognition in the experimental group (Table 3), accompanied by increased functional connectivity in the right inferior frontal gyrus (rIFG), right superior frontal gyrus (rSFG), and left superior parietal lobule (lSPL) (Figure 1). Chen et al. [61] conducted a study on young adults to examine the relationship between bilingual experience and executive functions. The sample comprised 60 participants, including 30 Chinese-English bilinguals and 30 Chinese monolinguals, who were matched based on age, intelligence, and socioeconomic status. The findings of the study demonstrated that bilingualism significantly predicted a better performance on both auditory and visual working memory tasks (Table 3).

#### 3.1.5. Artistic Performances and Live Cultural Experiences

Beyond structured learning activities such as music and language training, cognitively stimulating leisure activities also include the receptive and experiential engagement with artistic performances. Gabriel et al. [62] conducted a study on young and middle-aged adults (age range: 21–67 years) who watched a multidisciplinary performance combining dance, singing, and guitar, performed live or via video projection in a counterbalanced design within subjects. The results of the study demonstrated that the live performance elicited higher levels of subjective enjoyment and arousal than did the recorded version and the baseline conditions (Table 3). Anxiety levels exhibited a decrease after having seen both live and recorded performances (Table 3), with no significant differences observed between the two formats. Furthermore, physiological measurements indicated heightened emotional engagement during the live performance, as evidenced by increased autonomic activation and heart rate (HR) modulation in response to musical intensity (Figure 1). Concurrently, electroencephalogram (EEG) patterns suggested elevated levels of cognitive absorption during the live performance (Figure 1).

#### 3.1.6. From Receptive Artistic Experiences to Participatory Creative Activities

In addition to receptive artistic experiences, a substantial body of research has focused on participatory creative activities, which require active cognitive engagement, manual skills, and emotional involvement. These activities encompass drawing, visual arts, handicrafts, and other forms of creative production. Drake, Hastedt, and James [63] conducted an experimental study on young adults (age range: 19–25 years) who completed four drawing sessions over four consecutive days. Participants were assigned to one of two drawing conditions: drawing to express emotions or drawing to distract themselves from emotional content. The results indicated that engaging in drawing activities led to improvements in mood during the sessions, with distraction-focused drawing producing greater benefits than expressive drawing (Table 3). Specifically, participants in the distraction condition reported greater positive affect and life satisfaction, along with less negative affect (Table 3), both after a single session and after repeated sessions over four days. Notably, the emotional benefits of drawing persisted over time, suggesting that brief but repeated engagement in creative activities may have lasting positive effects on emotional well-being. Bone et al. [64] conducted a longitudinal study in which participants were followed from adolescence to late adulthood (age range: from 17–18 to 71–72 years), with a view to examining the long-term associations between engagement with the arts and cognitive functioning. Following the accounting for structural demographic and socioeconomic determinants that may influence both arts participation and cognition, the authors distinguished between participatory art activities (e.g., painting, music production, manual work) and receptive art activities (e.g., attending concerts, theatre performances, and museum visits). The findings indicated that engagement in the arts was associated with superior cognitive outcomes seven years later (Table 3). Specifically, it was observed that engaging in receptive art activities for a duration of up to three hours per week was associated with enhanced memory performance. In contrast, both participatory and receptive engagement for a duration of up to one hour per week were associated with improved executive functioning and language abilities (Table 3). Concurrent studies by Jiang et al. [65] examined the impact of a structured artistic intervention on the psychological well-being of young adults (average age: 21.28 ± 1.84). A substantial sample of participants was assigned to an intervention group, which took part in a lacquer painting artistic practice programme that was spread over several weeks, or to a control group that received no specific guidance or activities. Assessments of mental well-being, incorporating indicators of well-being, depression, anxiety, and stress, were collected prior to the intervention, immediately following the intervention, and two months subsequently. The findings indicated that, in comparison with the control group, participants engaged in artistic practice exhibited enhanced mental well-being, particularly a decrease in depression levels immediately following the intervention. Furthermore, a distinct configuration of relationships between mental health variables was observed in the intervention group, with social well-being being associated with reduced levels of stress and depression (Table 3). This pattern was not evident in the control group. However, these associations tended to diminish over time, suggesting that the effects of the artistic intervention, while significant in the short term, may not be sustained in the long term without ongoing involvement. Mashinchi et al. [66] conducted an observational study of older adults (age range: 67–89 years) to examine the association between involvement in handicraft art leisure activities (HALAs) and cognitive functioning. The sample consisted of older adults who were divided into two groups: those who participated in HALAs (such as sewing, quilting, knitting, crocheting, painting, drawing, woodworking, crafts, writing and gardening) and those who did not. The results indicated that participation in HALAs was associated with superior performance in specific cognitive domains, particularly in working memory and visuospatial and nonverbal reasoning tasks (Table 3). These associations were independent of factors commonly considered in the cognitive reserve literature, such as age, education level, and occupational history. Park et al. [67] conducted an experimental study on older adults (age range: 60–89 years) to assess the impact of different forms of engagement in daily life on cognitive function. Participants engaged in either productive engagement programs, which entailed acquiring novel complex skills over a period of at least 15 h per week for a duration of three months, or receptive engagement programs, involving participation in social activities and novel experiences without the acquisition of specific skills. Productive activities included courses in digital photography, quilting, and combinations of both. The cognitive functions assessed included episodic memory, processing speed, mental control, and visuospatial skills. The findings of the study demonstrated that the acquisition of novel competencies, notably digital photography, exhibited a positive impact on episodic memory, with a more modest effect on processing speed and visuospatial abilities (Table 3). Conversely, social and receptive activities alone did not elicit significant outcomes. Iizuka et al. [68] conducted a study on older adults to examine the effects of a creative and manual activity program (MONOZUKURI), including both crafts and creative food preparation, on cognitive functioning. Participants were randomly assigned to either an intervention group, which attended 12 weekly sessions of practical activities, or an active control group, which participated in health education classes. The results indicated that the intervention group (average age: 74.9 ± 5.5) demonstrated enhancements in executive functions (Table 3). Wang et al. [69] conducted a study on middle-aged and elderly adults (age range: 56–84 years) to evaluate the effects of a cooking training program on cognitive functioning. Participants were challenged with performing multitasking cooking activities, which entailed the simultaneous management of multiple food preparations and the execution of a concurrent secondary task, namely the setting of the table. The results of the study indicated that cooking training selectively improved executive control efficiency (Table 3).

#### 3.1.7. Music Listening

In contrast to active musical training, listening to music is a primarily receptive leisure activity that nonetheless engages emotional, cognitive and physiological processes. A number of studies have examined the effects of music listening on stress, emotional regulation, and cognitive performance. Linnemann et al. [70] conducted an experimental study on young adults (age range: 18–31 years) to assess the effect of listening to music on perceived stress. Participants were instructed to record their music listening activity and subjective stress levels multiple times daily. A subgroup of the participants provided saliva samples for subsequent analysis of cortisol and alpha-amylase levels. The findings indicated that music listening led to a reduction in perceived stress levels (Table 3), with greater effects observed when music was utilized for relaxation purposes. Furthermore, the implementation of relaxing music resulted in a reduction of cortisol concentrations (Figure 1), while energizing music led to an increase in alpha-amylase activity. van Goethem and Sloboda [71] conducted a study on young adults and middle-aged adults (age range: 18–64 years) to analyze the use of music in regulating emotions in everyday life. Participants recorded episodes of music listening in weekly diaries, indicating the emotional regulation strategies they adopted. The results showed that music was frequently used to modulate mood (Table 3), making it the second most commonly used strategy for regulating emotions after talking to friends. Ferreri and Rodriguez-Fornells [72] conducted a study on young adults (average age: 20.57 ± 4.11) to explore the influence of music on episodic memory and emotional experience. The participants, who were non-professional musicians, listened to excerpts of classical music and evaluated the degree of perceived musical gratification. The findings indicated that musical excerpts perceived as most rewarding were more likely to be remembered, and that individual differences in the capacity to experience musical gratification could serve as predictors of memory performance. The study posits the hypothesis that music-related reward responses may directly modulate higher cognitive functions, such as episodic memory, and thereby account for individual differences in memory performance (Table 3). Vincenzi et al. [73] conducted a study on older adults (age range: 65–75 years) with a view to examining the impact of listening to music on cognition and emotional state. Participants were exposed to musical excerpts characterised by positive or negative emotional valence, along with spoken recordings. These were followed by cognitive assessments before and after the listening experience. The results showed that listening to happy music improved working memory, increased arousal and improved mood. This was particularly evident when listening to longer music excerpts compared to spoken word recordings and negatively valenced music (Table 3). Conversely, sad music did not evoke analogous effects. However, at the individual level, improvements in working memory were not directly related to changes in mood or arousal, suggesting that happy music can optimise cognitive performance in older adults even independently of emotional effects.

#### 3.1.8. Musical Activities

In addition to play, musical activities such as learning and playing a musical instrument represent a complex form of cognitive stimulation. This is because they simultaneously involve perceptual, motor, executive, and emotional processes. Several studies have demonstrated both cognitive and emotional effects in learning and/or playing a musical instrument. MacRitchie et al. [74] examined the effects of a 10-week piano training program on the cognitive and motor skills of healthy elderly beginners (over 65 years old), demonstrating an improvement in visuomotor skills (Table 3). In 2011, Hanna-Pladdy and MacKay [75] compared three distinct groups (age range: 60–83 years) in a comparative study. The first group comprised non-musicians, defined as individuals who had never received formal training as instrumental musicians and lacked the ability to play an instrument, or read music. The second group consisted of low-activity musicians, characterised by experience playing a musical instrument for a minimum of 1 year, but less than 9 years, and formal training. The third group, referred to as high-activity musicians, included individuals who had a minimum of 10 years of regular experience playing a musical instrument and formal training. Subsequent post hoc analyses indicated that high-activity musicians exhibited superior cognitive performance in comparison to non-musicians, particularly with regard to nonverbal memory recall, visuomotor speed, cognitive flexibility, and naming (Table 3). Conversely, no significant disparities were observed in semantic and letter fluency skills. Furthermore, regression analyses demonstrated that years of musical practice and age of instrument acquisition were significant predictors of cognitive performance, particularly for visuospatial memory and processing speed. Seinfeld et al. [76] conducted a comparative analysis of the cognitive and emotional performance of two groups of elderly individuals (age range: 60–84 years). The experimental group participated in a 4-month piano lesson programme, which comprised weekly meetings in small groups integrating music theory and instrumental practice, accompanied by daily individual practice. Conversely, the control group engaged in a diverse array of physical, artistic, and cognitive leisure activities. The results demonstrated significant enhancements in the piano training group with regard to executive functions, particularly in terms of inhibitory control and divided attention processes (Table 3), and a tendency towards improvement in visuomotor and visual scanning skills. The intervention also produced a reduction in depressive symptoms, an improvement in positive affective states, and an improvement in psychological and physical quality of life (Table 3). With regard to motor skills, both groups demonstrated significant enhancements. Wang et al. [77] conducted a longitudinal study of older adults with the objective of examining the effects of learning a musical instrument on age-related cognitive and brain decline. The initial sample comprised 53 participants, with a 4-year follow-up on 32 subjects (average age: 76.00 ± 5.44). Within this group, one subgroup continued to engage in musical training over time, while another subgroup ceased their musical training and instead participated in alternative recreational activities, such as table tennis, gymnastics, and stretching exercises at the centre. The intervention programme comprised progressive instrumental training, initially spanning 12 weeks and subsequently extended to 16 weeks. This encompassed the reading of musical scores, exercises designed to enhance finger dexterity and independence, and the execution of musical performance tasks of escalating complexity. The findings indicated that the group who sustained their engagement in music training exhibited superior preservation of verbal working memory (Table 3) over a period of four years in comparison to those who had discontinued this activity. At the neural level, this cognitive preservation was associated with greater integrity of subcortical structures, particularly the right putamen, and more efficient functioning of the cerebellum, indicating a reduced need for cortical compensatory mechanisms (Figure 1).

**Figure 1:**
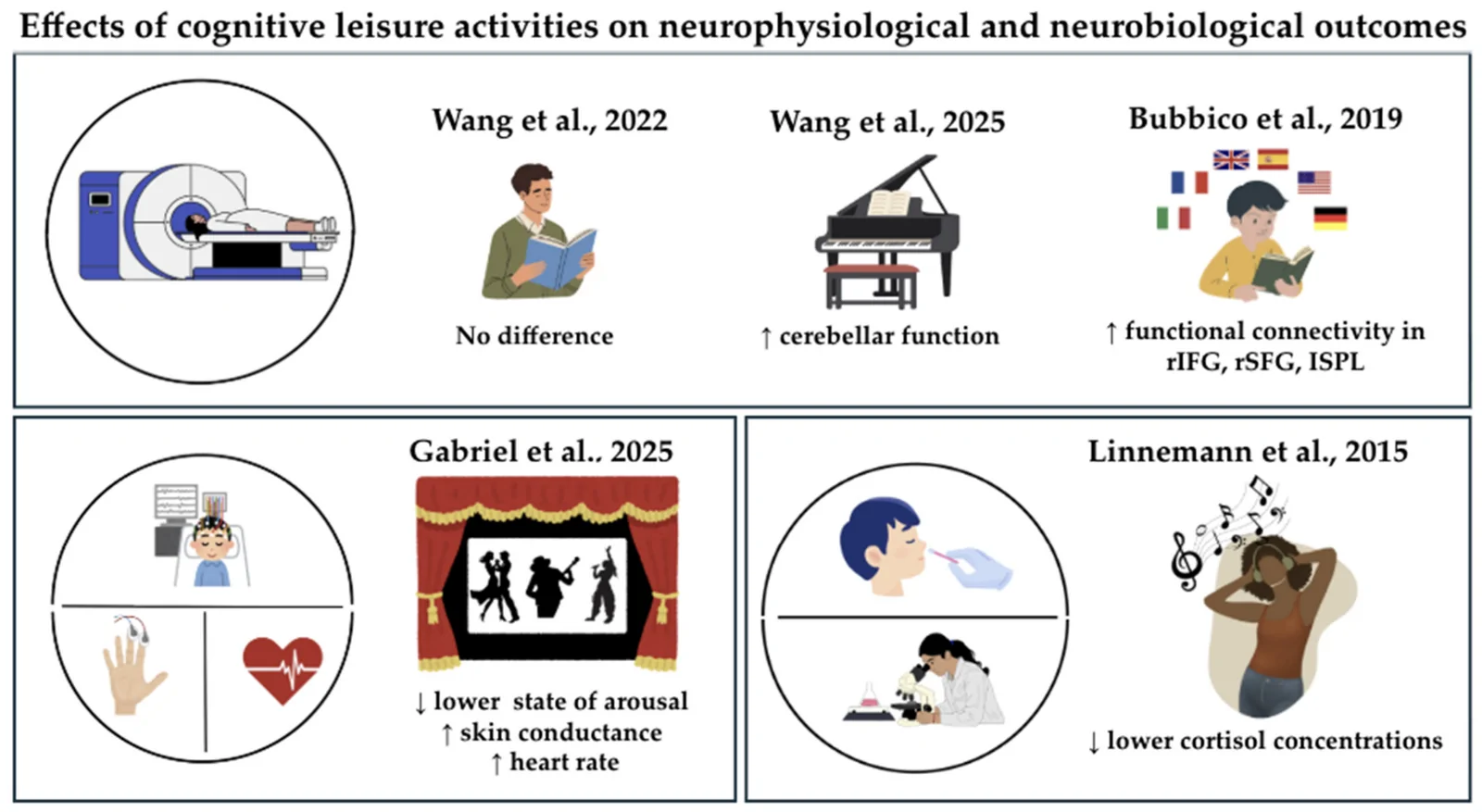
Schematic overview of the selected studies illustrating the impact of cognitive leisure activities on neurophysiological and neurobiological outcomes, assessed through instrumental techniques (fMRI, EEG, electrocardiogram - ECG, electrophysiology -EP) and blood and saliva analyses. Arrows indicate the direction of change (upward = increase; downward = decrease). At the top, MRI studies [77] report increased cerebellar function in piano players. Wang et al. [53] found no significant differences in cortical thickness or hippocampal volume between readers and non-readers, while Bubbico et al. [60], reported increased functional connectivity in specific regions among participants learning a new language. The bottom left panel [62] reduced neural activation and increased skin conductance and HR in participants who danced and sang while playing an instrument, assessed with EEG, ECG, and EP. The bottom right panel [70] reports decreased cortisol levels in participants listening to music, measured from saliva samples.

**Table 3:** Effects of cognitive-based leisure activities on cognitive functioning, emotional well-being, and neurobiological/neurophysiological outcomes.

Reference	Population	Sample Size (*n*)	Sex	Age Range (Years)	Average Age (Years)	Study	CR Measured by Questionnaire	LA Measured by Questionnaire	Specific Activity	Frequency/Duration	Cognitive or Emotional Outcome	Cognitive or Emotional Function Measure	Outcome Assessment Timing	Acute/Long-Term Effects	Improvement	Neurophysiological/Neurobiological Outcome
***Reading***																
Wang et al., 2022 [53]	Middle-aged and older adults	459	F: 56.6%	40–100	60.14 ± 8.69	Cross-sectional study	No	In-house measure	Reading	-	Cognitive: attention, language, memory, visual construction skills, inhibition	MMSE, MoCA, CDR, RAVLT, ROCF, Stroop Color–Word, Test-Victoria version, TMT, CDT, BNT, SDMT, NPI	-	-	↑ attention ↑ memory ↑ language ↑ visuospatial ↑ executive function	Yes: MRI
Mao et al., 2020 [54]	Older adults	10,741	F: 54.4%	83–93	-	Prospective population-based longitudinal cohort study	No	In-house measure	Reading books or newspapers and other (watching TV, listening radio, playing cards or mahjong)	-	Cognitive: screening within: orientation, registration, attention, language, memory, visual construction skill	MMSE	Baseline survey and 3.4-year follow-up	-	↑ general cognitive abilities	No
***Expressive writing***																
Klein and Boals, 2001 [55]	Young adults	Study 1: 71 Study 2: 101	Study 1: F: 60.6% Study 2: F: 58.4%	Study 1: 18–19 Study 2: -	-	Randomized experimental trials	No	In-house measure	Writing	3 sessions	Cognitive: working memory	Study 1: OSPAN Study 2: OSPAN, IES	Exp 1: Baseline, 1-week, and 7-week post-writing; Exp 2: Baseline, immediate, and 7–8 week post-writing	Mixed acute and long-term effects	↑ working memory ↓ intrusive and avoidant thinking about a stressful experience	No
Cheung and Ng, 2021 [56]	Young adults	121	F: 64.46%	18–25	21.81 ± 1.67	RCT (3 conditions)	No	In-house measure	Writing	3 consecutive days (20 min/day)	Emotional: emotional regulation, cognitive reappraisal, depressive symptoms, general health, and life satisfaction	ERQ, Patient Health Questionnaire-9, 5-item general health subscale of the SFHS-36, 5-item Satisfaction with Life Scale	Baseline (Day 1), 1-day follow-up (Day 5), and 1-month follow-up (Day 35)	Mixed acute and long-term effects	↑ cognitive reappraisal ↑ expressive suppression ↑ life satisfaction ↑ general health over time	No
***Gaming and puzzle-based activities***																
Urwyler et al., 2023 [57]	Middle-aged and older adults	12	F: 50%	45–75	58.92 ± 10.28	Pilot feasibility randomized crossover trial	No	No	Puzzle games	8 weeks	Cognitive and Emotional: working memory, attention, executive function, mood, anxiety	TAP task, MoCA, TMT, Snellgrove Maze Test, POMS, STAI, QoL questionnaire	3 measurement waves: Pre-test (baseline), Mid-test (8 weeks), Post-test (16 weeks)	Mixed acute and long-term effects	↑ visual attention ↑ visuospatial	No
Martinez et al., 2023 [58]	Young adults and middle-aged	496	F: 53.61%	18–59	M = 28.08	Cross-sectional correlational study	No	In-house measure	Video games and board games	-	Cognitive: fluid intelligence, mental flexibility, planning, visual and verbal working memory, visual spatial processing	Raven SPM38 (series D and E), Monsell Task, ToL, CBT, operation span task, visual search task	-	-	↑ mental flexibility ↑ planning ↑ visual working memory ↑ visuospatial processing ↑ fluid intelligence ↑ verbal	No
Kaynak et al., 2023 [59]	-	112	F: 83.04%	-	-	Quasi-experimental pretest-posttest study	No	In-house measure	Crossword for education	14 weeks	Cognitive: problem solving	PSI-A, CDMNS	Pre-test and Post-test	Acute effects	↑ problem solving ↑ decision making	No
***Linguistic activities and bilingualism***																
Bubbico et al., 2019 [60]	Middle-aged and older adults	26	F = 73.08%	59–79	IG: 69.5 ± 5.3 CG: 65.7 ± 3.7	Controlled intervention trial	No	In-house measure	Learning a new language	4 months (120 min/week of second language training)	Cognitive: global cognition, attention, cognitive flexibility, phonological lexicon, short and long episodic memory, executive function	MMSE, TMT, FAS verbal fluency, Babcock Memory test, FAB	Pre-intervention (T0) and Post-intervention (T1/4 months)	Acute effects	↑ global cognition	Yes: MRI
Chen et al., 2025 [61]	young adults	60	F = 78.33%	-	Bilinguals: 20.21 ± 1.34 Monolinguals: 19.96 ± 1.58”	Cross-sectional study	No	LSBQ (Chinese version) [78]	Bilingual	-	Cognitive: inhibitory control, working memory, and cognitive flexibility	VST, AST, VNB, ANB, VTS, ATS	-	-	↑ visual working memory	No
***Artistic performances and live cultural experiences***																
Gabriel et al., 2025 [62]	Young adults and middle age	27	F = 70.37%	21–67	M = 39.37	Crossover design	No	In-house measure	Performance combining dance, singing, and guitar, presented either live or as a video projection	Duration of theater performance (3 structural segments: S1, S2, S3)	Emotional: pleasantness, anxiety, anhedonia	BMIS, STAI-Y, Chapman Physical anhedonia scale	Pre-experiment, post-video performance, and post-live performance	Acute effects	↑ global emotion ↑ arousal ↓ anxiety	Yes: EEG, electrodermal and cardiac activities
***From receptive artistic experiences to participatory creative activities***																
Drake, Hastedt and James, 2016 [63]	Young adults	40	F = 57.5%	19–25	21.4 ± 1.2	Experimental trial (randomized condition assignment)	No	No	Drawing	4 sessions over 4 days	Emotional: mood	SWLS, PANAS	Time 1 (Baseline), Time 2 (after 1 day/session), and Time 3 (after 4 days/sessions)	Acute effects	↑ positive affect ↓ negative affect ↑ life satisfaction	No
Bone et al., 2024 [64]	Older adults	3245 for memory and 2926 for EF/language	F = 53%	from 17–18 to 71–72 *	-	Prospective longitudinal observational cohort study	No	In-house measure	Participatory (e.g., painting, making music, crafts) and receptive (e.g., concert, play, museum) arts engagement separately	-	Cognitive: working memory (immediate recall, delayed recall, and digit ordering tests), EF/language (similarities, letter fluency, and category fluency test)	Similarities test (WAIS), LF, CF, DOT, IWR, DWR from TICS	-	-	↑ executive function ↑ language ↑ memory	No
Jiang et al., 2025 [65]	Young adults	353	IG: F = 57.37% CG: F = 55.78%	-	21.28 ± 1.84	Double-blind RCT	No	In-house measure	Underwent lacquer painting art practice	Total of 48 h, twice a week, with each session lasting 3 h	Emotional: well-being, depression, anxiety, stress	MHC-SF, DASS-21	Baseline (T1), Post-intervention (T2), and Follow-up (T3)	Mixed acute and long-term effects	↓ depression ↓ stress ↑ well-being	No
Mashinchi et al., 2024 [66]	Older adults	50	F = 68%	67–89	79.82 ± 6.28	Cross-sectional study	No	In-house measure	HALAs	-	Cognitive: Verbal Comprehension Index, Perceptual Reasoning Index, Working Memory Index, Processing Speed Index	WAIS: SIM, VOC, INF, BD, MRT, VP, DS, AR, SSp, CD	-	-	↑ abstract visual information ↑ verbal reasoning ↑ working memory	No
Park et al., 2014 [67]	Older adults	221	F = 73.9%	60–90	71.67 ± 7.29	Randomized controlled intervention trial	No	In-house measure	Quilt, photo, and dual conditions	3 months (15 h/week)	Cognitive: processing speed, mental control, episodic memory, visuospatial processing	DCT, FT, Cogstate, HVLT-M, CANTAB, VRM, SMT, SOC, RPM-M	Pre-intervention (baseline) and Post-intervention (after 14 weeks)	Acute effects	↑ episodic memory ↑ visuospatial processing ↑ processing speed	No
Iizuka et al., 2025 [68]	Older adults	49	F = 83.67%	-	IG: 74.9 ± 5.5 CG: 75.4 ± 6.3	Single-blind RCT	No	In-house measure	Handicrafts and creative cooking	3 months	Cognitive: executive function, verbal ability, attention, processing speed, memory, and working memory	TMT, verbal fluency tasks: LF, CF, CD- subtest of the WAIS, LM- I and II subtests from WMS-R, VMST from the WMS-R, DS from the WAIS	Pre-intervention (baseline) and Post-intervention (after 3 months)	Acute effects	↑ executive function	No
Wang et al., 2011 [69]	Middle age and older adults	57	F = 61%	56–84	IG: 62.9 ± 5.6 CG: 65.5 ± 7.4	Mixed-design controlled cognitive intervention	No	In-house measure	Cooking training	5 weeks (1 session/week, ~30–60 min per session)	Cognitive: attention, working memory	WAIS DS, arithmetic, LNS, digit symbol coding, and SSp	Pre-test (baseline) and Post-test (after 5 weeks)	Acute effects	↑ executive control processing	No
***Music listening***																
Linnemann et al., 2015 [70]	Young adults	55	F:63.64%	18–31	23.20 ± 3.11	Within-subject Ecological study	No	In-house measure and MPQ [79]	Music listening	5 consecutive days across 2 conditions	Emotional: stress	Momentary subjective stress levels were measured using the item ‘At this moment, I feel stressed’	6 momentary daily assessments across two 5-day measurement waves	-	↓ stress	Yes: salivary cortisol
van Goethem and Sloboda, 2011 [71]	Young adults and middle age	44	F:52.27%	18–64	M = 28.7	Experience sampling/Event-based diary study	No	In-house measure	Music listening	-	Emotional: affect regulation	Booklet with short questionnaires	Event-based sampling immediately after music listening episodes	-	↑ affect regulation	No
Ferreri and Rodriguez-Fornells, 2017 [72]	Young adults	30	F:66.67%	-	20.57 ± 4.11	Within-subject	No	In-house measure and BMRQ [80]	Music listening	-	Cognitive and emotional: episodic memory and emotional valence	MBEA, Physical Anhedonia Scale	Encoding phase (Day 1) and Recognition test (24 h retrieval)	Acute effects	↑ memory	No
Vincenzi et al., 2022 [73]	Older adults	132	F:46.21%	65–75	69.39 ± 3.06	Experimental study	No	AFML [81]	Music listening	Single auditory exposure session: Short (≈2.5 min) vs. Long (≈8 min)	Cognitive: WM and executive functions (verbal fluency, cognitive flexibility, and speed), and arithmetic ability	SVAMA, WAIS-, PSW-B, PANAS, SAM, TMT, backward Corsi blocks, AC-FL, and verbal fluency	Pre-test and post-test	Acute effects	↑ working memory ↑ arousal ↑ mood	No
***Music activities***																
MacRitchie et al., 2020 [74]	Older adults	17	F = 76.47%	over 65	70.9 ± 5.5	Waitlist-controlled intervention trial	No	In-house measure	Learning to play a musical instrument	10 weeks	Cognitive: visuo-motor skills	TMT	Pre-test and Post-test (3 testing waves for waitlist control)	Acute effects	↑ visuo-motor skills	No
Hanna-Pladdy and MacKay, 2011 [75]	Older adults	70	F = 50/60%	60–83	NM: 69.7 ± 7.9 LAM: 69.5 ± 6.6 HAM: 70.8 ± 6.3	Cross-sectional comparative study (3 lifespan activity groups)	No	In-house measure	Playing a musical instrument	-	Cognitive: verbal intellectual functioning, memory, attention, working memory, and language functions	AMNART, CVLT-II short version, WMS-II, Visual Reproduction I and II subtests, DS, subtest of the WAIS, LNS, SSp, Trails A, and Trails B, BNT, semantic and phonemic fluency	-	-	HAM: ↑ naming, ↑ nonverbal memory recall, ↑ visuomotor speed, ↑visuomotor sequencing, ↑ cognitive flexibility	No
Seinfeld et al., 2013 [76]	Older adults	29	F = 75.86%	60–84	IG: 69.30 ± 2.03 CG: 69.56 ± 1.43	Controlled intervention study	No	In-house measure	Playing a musical instrument	4 months (45 min/day practice + weekly piano lessons)	Cognitive and emotional: motor ability, attention, executive function, second for mood and QoL	FTT, Grooved Pegboard, Block Design forms part of the Spanish version of WAIS, SSF, SSB, TMT-, SDMT, Stroop Test, Formal Lexical Task, BDI, POMS, WHOQOL-BREF	Pre-test (baseline) and Post-test (immediately after 4 months)	Acute effects	↑ inhibitory control ↑ divided attention processes, ↑ motor ability ↑ mood ↓ depression ↑ subjective well-being	No
Wang et al., 2025 [77]	Older adults	32	F = 71.87%	-	76.00 ± 5.44	Longitudinal follow-up study	No	In-house measure	Playing a musical instrument	Initial 12–16 weeks + 4-year follow-up (continue vs. stop group)	Cognitive: verbal working memory, verbal memory, and executive control	DS, Verbal Fluency tasks, Logical Memory Test, RBMT, TMT	Baseline (pre-training), post-intervention, and 4-year follow-up	Mixed acute and long-term effects	↑ verbal working memory	Yes: MRI

Notes: The arrows indicate the direction of change (upward = increase; downward = decrease). * Age range refers to participants’ ages across the follow-up period (from adolescence to late adulthood). Abbreviations: AC-FL: Arithmetic Ability; AFML: Adaptive Functions of Music Listening Scale; AMNART: American National Adult Reading Test; ANB: Auditory N-Back Task; AR: Arithmetic; AST: Auditory Stroop Task; ATS: Auditory Task-Switching Paradigm; BBQ-36: 36-item bilingual questionnaire (Vietnamese-English); BD: Block Design; BDI: Beck Depression Inventory; BMIS: Brief Mood Introspection Scale; BMRQ: Barcelona Music Reward Questionnaire; BNT: Boston Naming Test; CANTAB: Cambridge Neuropsychological Test Automated Battery; CBT: Corsi Block-Tapping Task; CD: Coding; CDMNS: Clinical Decision Making in Nursing Scale; CDR: Clinical Dementia Rating; CDT: Clock Drawing Test; CF: Category Fluency Task; CG: Control Group; CR: Cognitive Reserve; CVLT-II: California Verbal Learning Test, Second Edition; DASS-21: Depression, Anxiety, and Stress Scales; DCT: Digit-Comparison Tasks; DOT: Digit Ordering Test (WAIS-III adapted); DS: Digit Span; EEG: Electroencephalogram; EF: Executive Function; ERQ: Emotion Regulation Questionnaire; F: Female; FAB: Frontal Assessment Battery; FT: Flanker Tasks; FTT: Finger Tapping Test; HALAs: handicraft art leisure activities; HAM: High Activity Musicians; HVLT: Hopkins Verbal Learning Test; IES: Horowitz’s Impact of Events Scale; IG: Intervention Group; INF: Information; I; LA: Leisure Activities; LAM: Low Activity Musicians; LF: Letter Fluency; LM: Logical Memory; LNS: Letter-Number Sequencing; LSBQ: Language and Social Background Questionnaire; M: Mean; MBEA: Montreal Battery of Evaluation of Amusia; MHC-SF: Mental Health Continuum—Short Form; MMSE: Mini-Mental State Examination; MoCA: Montreal Cognitive Assessment; MRT: Matrix Reasoning Task; MPQ: Music Preference Questionnaire; MRI: Magnetic Resonance Imaging; NM: Non Musicians; NPI: Neuropsychiatric Inventory; OSPAN: Arithmetic Operation–Word Memory Span Task; PANAS: Positive and Negative Affect Schedule; POMS: Profile of Mood States; PSI-A: Problem Solving Inventory Form-A; PSW-B: Psychological and Social Well-Being; QoL: Quality of Life; RAVLT: Rey Auditory Verbal Learning Test; RBMT: Rivermead Behavioral Memory Test; RCT: Randomized controlled trial; ROCF: Rey–Osterrieth Complex Figure Test; RPM: Raven’s Progressive Matrices; SAM: Self-Assessment Manikin; SDMT: Symbol Digit Modalities Test; SFHS: Short-Form Health Survey; SIM: Similarities; SMT: Spatial Memory Task; SOC: Stockings of Cambridge Task; SPM: Standard Progressive Matrices; SSp: Spatial Span; SS: Symbol Search; SSB: Spatial Span Backwards; SSF: Spatial Span Forward; STAI: State–Trait Anxiety Inventory; STAI-Y: State–Trait Anxiety Inventory-Y; SVAMA: Italian Checklist for Multidimensional Assessment; SWLS: Satisfaction with Life Scale; TAP: Test of Attentional Performance; TICS: Telephone Interview for Cognitive Status; TMT: Trail Making Test; ToL: Tower of London; VNB: Visual N-Back Task; VMST: Visual Memory Span Test; VOC: Vocabulary; VP: Visual Puzzles; VRM: Verbal Recognition Memory; VST: Visual Stroop Task; VTS: Visual Task-Switching Paradigm; WAIS: Wechsler Adult Intelligence Scale; WHOQOL-BREF: World Health Organization Quality of Life Brief Questionnaire; WM: Working Memory; WMS-III: Wechsler Memory Scale Third Edition; WMS-R: Wechsler Memory Scale–Revised.

### 3.2. The Effects of Social Leisure Activities

A growing body of research has examined the cognitive and emotional effects associated with participation in social leisure activities. Social activities constitute a heterogeneous domain, encompassing both informal and structured forms of interaction that vary in frequency, complexity, and degree of involvement. These activities include, but are not limited to, visiting friends and relatives, participating in religious or community events, volunteering, attending cultural and artistic venues, traveling, and participating in socially integrated recreational activities.

We included 29 studies referring to social leisure activities. 76% (22 out of 29) were observational studies (including cross-sectional and longitudinal design). The remaining 24% were experimental studies (including randomized–controlled trials and non-randomized studies). Among the experimental studies, 72% assessed outcomes immediately post-intervention (acute outcomes), 14% evaluated outcomes in the long term, whereas 14% evaluated outcomes in both the short and long term (mixed acute/long-term) (see Table 4 for further details).

#### 3.2.1. Reading and Game-Based Social Activities

In the context of socially integrated activities, studies have examined the potential cognitive benefits of shared reading and game-based practices. Weziak-Bialowolska et al. [82] conducted a large-scale study of middle-aged and older adults (age range: 50–80+ years), examining the role of socially integrated cognitive activities, including reading and gaming, in relation to emotional aspects. The authors distinguished between different sedentary forms of cognitive and social leisure, including activities such as reading books, magazines, and newspapers, word and number games played alone, and competitive games such as cards or chess played in social settings. With regard to the results relating to activities in the social domain, the study found prospective associations with greater happiness, lower levels of loneliness, and a reduced risk of Alzheimer’s disease (Table 4). Furthermore, occasional participation in these social activities was linked to greater optimism and a lower risk of depression (Table 4). In a similar vein, Plummer et al. [83] conducted a study that examined the effects of participation in an intergenerational book club. The book club met once weekly for 1 h for a period of 8 weeks, and the study’s sample population comprised young adults aged 19–24 and older adults aged 60–89. The results of the study indicate that adult and older participants exhibited substantial enhancements in their cognitive performance as a whole during the intervention period (Table 4). The emotional outcomes, which included depression, anxiety, stress and quality of life, demonstrated stability, potentially indicative of elevated baseline levels (Table 4).

#### 3.2.2. Travel as a Social Experience

In this section, travel is considered specifically in terms of its social dimension. It is defined as a form of recreational social activity that combines novelty, physical movement, and interpersonal interaction. This is treated in this section as a social leisure activity precisely because of this interpersonal component. In the study conducted by Baroi [84], the impact of a structured group travel experience on young adults (age range: 23–26 years) was examined. The results indicated a substantial decrease in perceived stress and an increase in happiness and life satisfaction following participation (Table 4). Rehman et al. [85] conducted a study on a large sample of young and middle-aged adults (age range: 22–60 years), finding that frequent leisure travel was positively associated with psychological well-being. This relationship was found to be partially mediated by stress reduction and improved social support (Table 4). In older populations, Pugh et al. [86] demonstrated that participation (age range: 57–97 years) in day trips and overnight trips was associated with better overall cognition (Table 4). In contrast, loneliness was associated with diminished semantic memory performance (Table 4). Subsequent analysis demonstrated that social activity exhibited a substantial interaction with the element of time, such that increased social participation was correlated with more effective maintenance of episodic memory and superior organisational skills, in addition to enhanced perceptual speed during the follow-up period. Greater social activity was also associated with superior performance in semantic memory and working memory (Table 4).

#### 3.2.3. Visiting Friends and Informal Social Contact

A substantial number of studies have focused on informal social interactions, such as visits to friends and relatives. Segel-Karpas and Lachman [87] examined middle-aged and older adults (age range: 32–84 years), demonstrating that frequent social contact (visits, phone calls, letters, or e-mails with family and friends) was positively associated with episodic memory and executive functions (Table 4), with personality traits moderating these effects. Two studies, Mai et al. [88] and Mao et al. [89], both of which were conducted on older adult populations (participants’ age ranged from 65 to 117 years in Mai et al. [88] and from 60 to 108 years in Mao et al. [89]), reported that regular social engagement was associated with better overall cognitive functioning and reduced loneliness (Table 4). In particular, Mai et al. [88] considered a wide range of activities, including domestic tasks such as housework, outdoor activities such as tai chi and group dancing, reading newspapers or books, visiting friends or relatives, playing cards or mahjong, caring for pets, and participating in organised social activities (Table 4). Mao et al. [89] examined social interaction and cognitive activities, including visiting friends and family, participating in community activities, using the internet, volunteering, and playing board games (for example, cards, mahjong and chess) (Table 4). Yu et al. [90] also found that greater diversity and frequency of social interactions in a large sample of adults (age range: 45–65 years) predicted cognitive scores in the domains of memory, mental integrity and total cognition (Table 4). In their study, Jang et al. [91] analysed a sample of community-dwelling older adults (age range: 50–109 years), focusing on informal, non-institutionalised forms of social engagement. Such forms of engagement included visits to friends and family, frequent social contacts, and spontaneous helping behaviours. The activities were conducted exclusively in person and reflected unstructured daily social interactions. The study demonstrated that a higher frequency of such interactions was associated with superior overall cognitive performance (Table 4). Zhaoyang et al. [92] conducted a study on adults and older adults (≥70 years) using a daily assessment approach. Participants reported on a daily basis on the quality and enjoyment of social interactions, including informal conversations and spontaneous social interactions. The study demonstrated that social interactions experienced as more enjoyable were associated with enhanced cognitive performance in memory binding and processing speed (Table 4).

#### 3.2.4. Community and Social Engagement: In-Person Versus Technology-Mediated Participation

This section examines social engagement through the lens of participation modality—in person versus technology mediated. In person engagement is considered here specifically through religious and community-based activities, while technology-mediated social engagement is examined more broadly, irrespective of its religious or community character. Both have been studied as a potential protective factor for cognitive functioning and socio-emotional well-being in older age.

##### In-Person Social Activities

Borgeest et al. [93] (conducted a study on a population of cognitively healthy older adults (age range: 18–88 years), with the aim of reconstructing in-person participation in religious and community activities (e.g., attendance at religious services, social events, and informal gatherings) throughout adulthood. The findings indicated that an elevated cumulative frequency of social and religious engagement was correlated with enhanced cognitive performance in advanced age, even after adjusting for factors such as education, health status, and socioeconomic conditions (Table 4).

##### Technology-Mediated Social Activities

Jeon et al. [94] examined a sample of older adults (age range: 50–99 years), assessing both in-person social activities (meetings with friends and family, participation in religious services) and digital social activities, such as online communication, participation in virtual groups, and social interactions mediated by technological platforms. The study demonstrated that increased social participation, irrespective of mode (offline or online), was associated with enhanced levels of cognitive functioning (Table 4) at both baseline and follow-up, suggesting that digital interactions may offer a partial compensation for reduced in-person social opportunities. Dodge et al. [95] conducted a study of socially isolated older adults (≥75 years), assigning participants to either an online conversational engagement intervention or a control condition. The intervention comprised structured, regular conversations via digital platforms, which were designed to stimulate verbal interaction and social engagement. The behavioural outcomes demonstrated significant improvements in semantic fluency and social satisfaction (Table 4). Furthermore, resting-state fMRI analyses demonstrated heightened functional connectivity within the dorsal attention network (DAN) (Figure 2). Mois et al. [96] conducted a study on a population of older adults (age range: 65–99 years) at risk of social isolation, who underwent a socialisation intervention based on group videoconferencing with predominantly relational content. The activities, conducted entirely online, involved regular meetings focused on social sharing. The results demonstrated a substantial decrease in loneliness (Table 4). Nakahara et al. [97] conducted a study on older adults (≥65 years), engaged in social activities facilitated by information and communication technologies, encompassing online communication and participation in digital social networks. The study demonstrated that increased digital social participation was associated with enhanced verbal memory performance and reduced levels of loneliness (Table 4).

#### 3.2.5. Volunteering and Social Clubs

Volunteering and participating in social clubs are structured forms of social engagement that can have long-term benefits. While the previous section addressed community and religious belonging expressed through attendance and informal participation, this selection focused on a more structured, role-based form of civic engagement, namely volunteering for an organisation and formal membership in clubs or associations. In a large study conducted in 12 European countries, Cohn-Schwartz [98] showed that participation (age range: 60–101 years) in volunteering and social clubs predicted better cognitive functioning and mental health four years later (Table 4). Hyun et al. [99] found that volunteering (age range: 70–91 years) and other forms of social participation (e.g., volunteer work for religious, charitable, political, health-related or other organisations; meetings of any organised group; socialising with friends or relatives) were associated with reduced short-term cognitive variability (Table 4). Similarly, Chen et al. [100], in a sample of middle-aged and older adults (≥45 years), reported that community participation (e.g., community organisation activities, school attendance, training courses, interacting with friends, playing mahjong, chess or cards, visiting a community activity room, dancing, exercising or practising qigong) and volunteering were associated with better cognitive functioning (Table 4). Ma et al. [101] studied the role of perceived social support, such as the availability of emotional, informational, or instrumental help from others. The participants’ age range was 65–116 years. Social support from family and friends was defined as contact with family and friends (who one talks to most; who one talks to first when they have something on their mind or an idea; who they ask for help first when they have problems or difficulties), assistance with illness (whether family members provided assistance when participants were ill) and money received (whether participants received money from their children). Social support from outside the family included perceptions of the availability of community social services and social insurance in a large cohort of cognitively healthy older adults. Higher levels of support from family, friends and community services were associated with a lower risk of cognitive decline (Table 4). Similarly, Son et al. [102] found that active participation in social organisations (e.g., religious groups, senior clubs, revolving credit associations, recreational/cultural/sports groups, alumni associations, hometown alliances, clan gatherings, volunteer groups, political parties, and interest groups) was associated with better cognitive functioning in a longitudinal study of Korean older adults (age range: 55–90 years). The frequency of association activities was associated with better preserved cognitive functioning (Table 4).

#### 3.2.6. Artistic and Cultural Social Activities

Participation in social, cultural and artistic activities has been associated with both cognitive and emotional benefits. Fancourt et al. [103] studied a large group of older adults (age range: 52–90 years). They found that attending museums, galleries, theatres and concerts was linked to slower cognitive decline. This was particularly true in terms of memory and semantic fluency (Table 4). Rathje et al. [104] conducted three experimental studies on adults (average age: 61.59 ± 14.70; 53.34 ± 16.58; 55.22 ± 18.54) and demonstrated that attending live theatre performances increases empathy, prosocial attitudes and charitable behaviour (Table 4). Similarly, Kühnapfel et al. [105] demonstrated that participating (age range: 21–79 years) in a public art exhibition resulted in a more positive mood, a greater sense of connection, increased well-being, and a reduction in negative mood (Table 4). Pitkala et al. [106] reported that participation (age range: 75–92 years) in group artistic and leisure activities led to improvements in cognitive functioning and psychological well-being (Table 4).

#### 3.2.7. Social Activities Related to Music

The emotional regulation associated with socially shared musical experiences has also been examined. Linnemann et al. [107] conducted an experimental study with young adults (age range: 20–32 years) which demonstrated that listening to music in a social setting was associated with reduced stress levels, as evidenced by subjective reports (Table 4) and physiological indicators (Figure 2).

#### 3.2.8. Mixed Social Activities

Several studies have examined the impact of participating in a combination of social activities, which often include informal interactions, community involvement and recreational pursuits. Wang et al. [108] examined an elderly population (average age: 71 ± 5) and found that participating in a variety of social, cognitive and physical activities—social activities such as visiting family or friends, receiving guests at home, and giving advice; cognitive activities such as sewing, weaving, reading, playing a musical instrument, playing cards, chess, mahjong, and attending the Beijing Opera; and physical activities such as gardening, walking, and group exercise—was associated with a slower rate of cognitive decline. This was particularly evident in social activities, where better overall cognition was observed (Table 4), as well as in activities involving greater mental engagement, where improvements in language and executive functions were noted. Conversely, greater physical activity was associated with better episodic memory. Krueger et al. [109] examined a wide range of social activities in older adults (average age: 80.2 ± 7.5), including participation in religious events, volunteering, social visits, and travel. Greater overall social involvement was associated with better global cognition, particularly with regard to working memory, perceptual speed, and visuospatial ability (Table 4). More recently, Dixon et al. [110], in a sample of older adults (age range: 60–79 years), reported that greater involvement in mixed social activities (e.g., visiting friends or family, going to the community centre, volunteering, and attending or participating in religious services) was associated with better global cognition.

**Figure 2:**
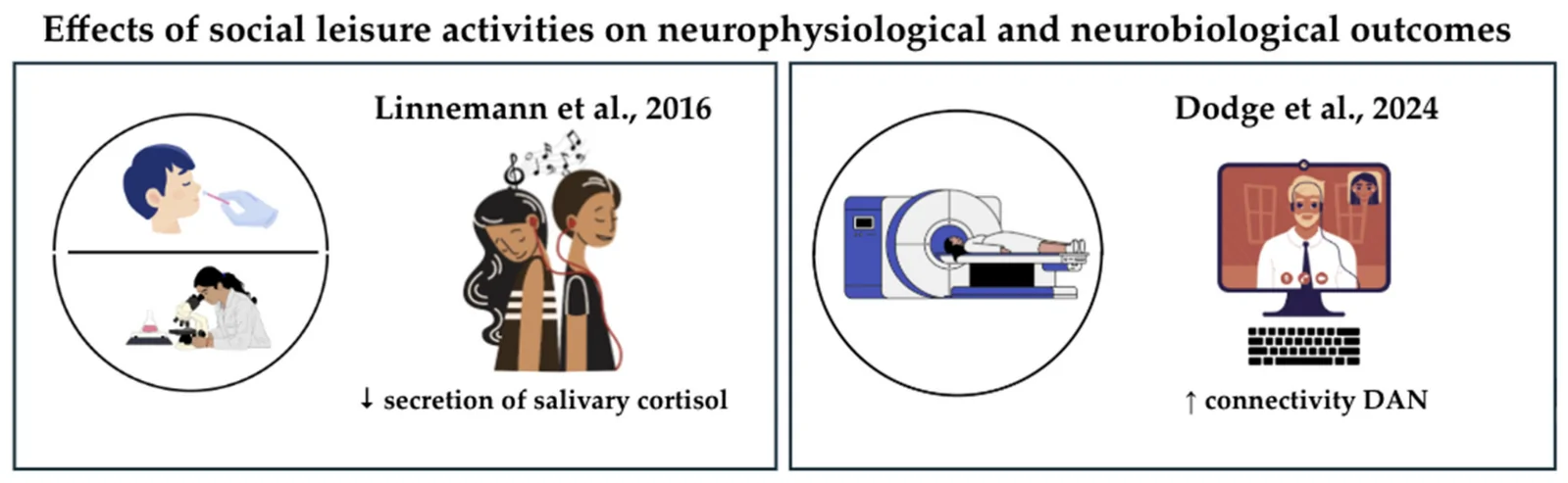
Schematic overview of selected studies illustrating the impact of social leisure activities on neurophysiological and neurobiological outcomes, assessed using instrumental techniques (fMRI) and saliva analysis. Arrows indicate the direction of change (upward = increase; downward = decrease). A study by Linnemann [107] demonstrated a decline in cortisol levels through the analysis of saliva samples from participants who listened to music in the presence of others. The panel on the right presents a study [95] that utilised fMRI to demonstrate an augmentation in connectivity within the Dorsal Attention Network in participants engaged in interpersonal online conversations.

**Table 4:** Effects of social-based leisure activities on cognitive functioning, emotional well-being, and neurobiological/neurophysiological outcomes.

Reference	Population	Sample Size (*n*)	Sex	Age Range (Years)	Average Age (Years)	Study	CR Measured by Questionnaire	LA Measured by Questionnaire	Specific Activity	Frequency/Duration	Cognitive or Emotional Outcome	Cognitive or Emotional Function Measure	Outcome Assessment Timing	Acute/Long-Term Effect	Improvement	Neurophysiological/Neurobiological Outcome
***Reading and game-based social activities***																
Weziak-Bialowolska et al., 2023 [82]	Middle aged and older adults	19,821	F: 59.29%	50–80+	64.5 ± 8.72	Prospective longitudinal study	No	In-house measure	Social mind games	-	Emotional: wellbeing, depression	Loneliness scale, EURO-D ≥ 4, ADL, IADL, Cognitive Impairment Date orientation	Pre-baseline and 6-year follow-up	-	↓ depression ↓ cognitive impairment ↓ loneliness ↑ wellbeing ↑ happiness ↑ optimism	No
Plummer et al., 2023 [83]	Young and older adults	28	F: 85.7%	19–24 60–89	70.71 ± 8.86	Pilot quasi-experimental study	No	No	Book club	8–10 weeks	Cognitive and emotional: cognition, fluency, depression, anxiety, stress, quality life	MoCA, DASS-21, GDS, OPQOL-brief	Pre-intervention (baseline) and Post-intervention	Acute effect	↑ global cognition	No
***Travel as a social experience***																
Baroi, 2024 [84]	Young adults	35	F: 34.3%	23–26	24.03 ± 0.89	Quasi-experimental study (pre-test/post-test control group design)	No	In-house measure	Travel and recreational activities	Short-term vacation/respite period	Emotional: stress, happiness, life satisfaction	PSS, SWLS, OHQ	Pre-test and Post-test	Acute effect	↓ stress ↑ happiness ↑ life satisfaction ↑ well-being	No
Rehman et al., 2025 [85]	Young and middle-aged adults	1051	F: 45.39%	22–60	-	Cross-sectional study	No	In-house measure (LTQ)	Travel	-	Emotional: stress, social support	PSS-10, WEMWBS, SSRS	-	-	↑ well-being ↓ stress ↑ social support	No
Pugh et al., 2020 [86]	Older adults	617	F: 77%	57–97	73.31 ± 6.43	Longitudinal prospective cohort study	No	In-house measure	Go on day trips or overnight trips	-	Cognitive and emotional: episodic memory, working memory, semantic memory, perceptual orientation, and perceptual speed, Perceived discrimination, Loneliness	WLM; WLR; WL-ReG, LM from WMS-R, DS from the WMS-R, Digit Ordering, BNT, CERAD-NB, 15-item Wide Range Achievement Test reading test, Line Orientation, RMP, SDMT, Stroop. Detroit Area Study Everyday Discriminations Scale, Jong-Gierveld Loneliness Scale	Longitudinal annual follow-up (up to 14 visits; mean 4.19 visits)	-	↑ global cognition ↑ episodic memory ↓ loneliness ↑ semantic memory ↑ working memory ↑ perceptual organization ↑ perceptual speed	No
***Visiting friends and informal social contact***																
Segel-Karpas and Lachman, 2016 [87]	Middle-aged and older adults	3524	F: 55%	32–84	55.8 ± 12.31	Cross-sectional survey study	No	In-house measure	Visits, phone calls, letters, or e-mails with family and friends	-	Cognitive: episodic memory, executive functioning	BTACT, immediate recall and delayed recall of a list of 15 words, WM span, VF, inductive reasoning, processing speed task	-	-	↑ episodic memory ↑ executive functioning	No
Mai et al., 2023 [88]	Older adults	12,852	F: 54.60%	65–117	84.02 ± 11.19	Cross-sectional study	No	In-house measure	Informal social and leisure activities	-	Cognitive: orientation, calculation, recall, and language	MMSE	-	-	↑ global cognition ↓ loneliness	No
Mao et al., 2023 [89]	Older adults	9803	F: 50.58%	60–108	68.68 ± 6.57	Cross-sectional study	No	In-house measure	Social interaction and volunteering, board games	-	Cognitive: orientation, episodic memory, calculation, language, naming, and visuospatial ability	TICS	-	-	↑ global cognition	No
Yu et al., 2025 [90]	Middle aged adults	8089	F: 51.12%	45–65	54.87 ± 5.92	Longitudinal study	No	In-house measure	Social engagement activities and volunteering, board games	-	Cognitive: orientation, numeric ability, recall tasks, visuospatial ability, episodic memory,	TICS	7-year follow-up across 4 waves (2011, 2013, 2015, 2018)	-	↑ global cognition ↑ memory ↑ mental intactness	No
Jang et al., 2026 [91]	Older adults	29,777	F: 58.8%	50–109	M = 62.7	Longitudinal panel cohort study	No	In-house measure	Informal social visits and helping	-	Cognitive and emotional: immediate word recall, delayed word recall, serial 7 s subtraction, and backward counting from 20, depressive mood	m-TICS, CES-D	14-year longitudinal follow-up across 8 waves (1998–2012)	-	↑ global cognition	No
Zhaoyang et al., 2021 [92]	Older adults	312	F: 67%	≥70 years	76.965 ± 4.849	Ambulatory Assessment/EMA study	No	In-house measure	Direct and digital social interaction	-	Cognitive: processing speed and attention, spatial working memory, intra-item feature memory binding	Mobile cognitive tests—Symbol search, Grid memory, color shape	16-day EMA protocol (5 momentary assessments per day)	-	↑ memory binding ↑ processing speed	No
***In-person religious and social activities***																
Borgeest et al., 2020 [93]	Young, middle-aged and older adults	708	F: 50.7%	18–88	53.4 ± 18.62	Cross-sectional study	LEQ	In-house combined measure and LEQ [17]	Social outings, religious activities, travel	-	Cognitive: Executive Function, Language Functions, Emotional Processing, Memory, Motor and Action Function	HT, STW, SC, PPP, VF, PC, FR, ERR, VSTM, SR, CMS, CMCV	-	-	↑ global cognition	No
***Technology-mediated social activities***																
Jeon et al., 2024 [94]	Middle aged and older adults	3650 (cross-sectional)—2034 (longitudinal)	F: 59.6%	50–99	67.68 ± 9.77	Prospective cohort study (cross-sectional and longitudinal on a subsample)	No	In-house measure	Internet-Based Social Activity and Face-to-Face Social Activity	-	Cognitive: episodic memory, WM, and attention and processing speed	The immediate and delayed recall of a set of 10 words, serial 7 subtraction, and backward counting	-	-	↑ global cognition	No
Dodge et al., 2024 [95]	Older adults	86	F: 75.6%	75+	79.6 ± 4 80.0 ± 3.7	RCT	No	In-house measure and LSNS-6 [111]	Interpersonal conversations	12 months (6-month induction [4 times per week] + 6-month maintenance [2 times per week])	Cognitive and emotional: language-based executive function, episodic memory, well-being	CFA, CS-IR CS-DR from the NACC UDSV3. NIH-EB	Baseline, Month 6, and Month 12	Long-term effect (immediately after maintenance)	↑ semantic fluency ↑ social satisfaction	Yes: fMRI
Mois et al., 2025 [96]	Older adults	99	F: 25.38%	65–99	73.5 ± 6.3 74.6 ± 7.1	RCT	No	In-house measure and Lubben Social Network Index (LSNS) [112]	Small-group discussions	8 weeks (2 sessions/week)	Emotional: friendship, loneliness, quality of life	Friendship Scale, UCLA Loneliness Scale, Quality of Life	Baseline, Week 4, and Week 8	Acute effects	↓ loneliness	No
Nakahara et al., 2024 [97]	Older adults	113	F: 92%	65+	78.6 ± 5.2	Cross-sectional exploratory study	No	In-house measure and LSNS–6 [111]	Digital and phone social contact	-	Cognitive and emotional: word recall, loneliness, depressive mood	Mini-Co, TIL UCLA Loneliness Scale, CES–D.	-	-	↑ global cognition ↓ loneliness	No
***Volunteering social clubs***																
Cohn-Schwartz, 2020 [98]	Older adults	19,997	F: 57.1%	60–101	70.11 ± 7.29	Longitudinal prospective cohort study	No	In-house measure	Sports or social club, Volunteering	-	Cognitive and emotional: short-term verbal learning and memory, semantic memory and processing Speed, depressed mood, pessimism, irritability, quality of life	Rey’s Auditory Verbal Learning Test, Fluency (animals), EURO-D scale, CASP-12 scale	4-year longitudinal follow-up across 3 waves (T1: 2011, T2: 2013, T3: 2015)	-	↑ global cognition ↑ mental health	No
Hyun et al., 2025 [99]	Older adults	304	F: 68%	70–91	77.5 ± 4.8	Ambulatory Assessment/EMA study	No	In-house measure and (3-item social participation scale)	Organized groups and volunteering	-	Cognitive: processing speed, associative working memory	EMA mobile cognitive tasks—Symbol Match, Color–Shape Binding, Color-Dot	14-day EMA protocol (6 assessments/day)	-	↑ global cognition	No
Chen et al., 2026 [100]	Middle-aged and older adults	3048	-	≥45 years	M = 54.49	Longitudinal panel cohort study	No	In-house measure and	Community, social and leisure activities	-	Cognitive: orientation, immediate and delayed word recall, serial sevens subtraction, and a figure-drawing task	MMSE	Longitudinal panel waves	-	↑ global cognition	No
Ma et al., 2024 [101]	Older adults	7720	F: 48.9%	65–116	83.54 ± 10.78	Longitudinal prospective cohort study	No	In-house measure	Family and community support network.	-	Cognitive: localization ability, place localization ability, reaction ability, attention and calculation ability, repetition ability, recall ability, naming ability, understanding and self-coordination ability, and visuospatial ability.	Chinese version of the MMSE	10-year follow-up across 4 waves (2008–2018; mean 5.1 years)	-	↑ global cognition	No
Son et al., 2023 [102]	Middle-aged and Older adults	4731	F: 58.8%	55–90	64.97 ± 6.89	Longitudinal study	No	In-house measure	Organizational and social participation	-	Cognitive: orientation to time and place, short-term and long-term memory, arithmetic ability, language, and visual construction	Korean version of the MMSE	across 7 waves (2006–2018)	-	↑ global cognition	No
***Artistic and cultural social activities***																
Fancourt et al., 2018 [103]	Middle-aged and older adults	3445	F: 55.2%	52–90	62.9 ± 7.5	Longitudinal prospective cohort study	No	In-house measure	Cultural and artistic attendance	-	Cognitive: executive function since it involves self-initiated activity, organisation and abstraction, response inhibition and set shifting and calls upon memory and language processes, memory.	Test of semantic fluency and an immediate and delayed recall task	10-year follow-up (Wave 2 to Wave 7)	-	↑ semantic fluency ↑ memory	No
Rathje et al., 2021 [104]	Older adults	1622	F: 61.58%	-	61.59 ± 14.70 53.34 ± 16.58 55.22 ± 18.54	Pre-post experimental study	No	In-house measure	Going to the theatre	Single theatre performance	Emotional: Interpersonal reactivity index common measure of emotional empathy, Topic-specific empathy, Altruistic values	Seven-item Empathic Concern subscale of the Interpersonal Reactivity Index, seven-item Perspective Taking subscale, Two questions from the Empathic-Concern subscale.	Pre- and Post-performance assessment	Acute effects	↑ empathy ↑ altruistic values	No
Kühnapfel et al., 2025 [105]	Young, middle-aged and older adults	67	F: 53.73%	21–79	37.31 ± 14.32	Quasi-experimental field study	No	In-house measure	Going to an art exhibition	Single exhibition visit	Emotional: Neighborhood Connectedness, Satisfaction with Neighborhood, Well-being, Cognitive–Affective Experience	ICSS, NOSQ, UCL, list of 16 cognitive-affective phenomenal states	Immediate post-visit assessment	Acute effects	↑ positive mood ↑ satisfaction with neighborhood ↑ connectedness ↓ negative mood ↑ well being	No
Pitkala et al., 2011 [106]	Older adults	235	F:73.65%	75–92	80 ± 3.6 80 ± 4	Pilot quasi-experimental study	No	In-house measure	Peer support group participation	3 months	Cognitive: several areas of cognition	ADAS–Cog	Baseline, 3 months, 6 months, and 12 months	Mixed acute and long-term effects	↑ global cognition ↑ mental well-being	No
***Social activities related to music***																
Linnemann et al., 2016 [107]	Young adults	53	F: 60%	20–32	23.32 ± 3.08	Ambulatory Assessment/Experience Sampling Method (ESM) study	No	In-house measure	Music listening	-	Emotional: stress	Momentary subjective stress levels were measured using the item ‘At this moment, I feel stressed’	Momentary repeated assessments during daily life	-	↓ stress	Yes: salivary cortisol
***Mixed social activities***																
Wang et al., 2013 [108]	Older adults	1463	F: 49%	-	71 ± 5	Longitudinal prospective cohort study	No	In-house measure	Family and friend visits	-	Cognitive: global cognitive function, episodic memory, language, executive function	CSID, Word List Learning, Word List Recall, and IU Story Recall, Animal Fluency Test, Token Test	-	-	↑ global cognition	No
Krueger et al., 2009 [109]	Older adults	838	F: 75%	-	80.2 ± 7.5	Cross-sectional study	Cognitive Activity Scale	In-house measure	Advisory social support	-	Cognitive and emotional: summary measures of episodic memory (seven tests), semantic memory (three tests), working memory (three tests), perceptual speed (four tests), and visuospatial ability (two tests), depressive mood, nevroticism, distress	WLM, LM-I, LM-D, BNT-15, VF, NART-15, DS, digit ordering, oral SDMT, Stroop, JLO, SPM-16	-	-	↑ global cognition, ↑ working memory, ↑ perceptual speed, ↑ visuospatial ability	No
Dixon et al., 2025 [110]	Older adults	1951	F: 69.5%	60–79	68.2 ± 5.12	Cross-sectional study	No	In-house measure and	Social, religious and leisure engagement	-	Cognitive: episodic memory, executive functioning, and processing speed.	FCSRT, Story Recall, VPA, Word Fluency by Category, Word Fluency by Letter, Number Span, TMT.	-	-	↑ global cognition	No

Notes: The arrows indicate the direction of change (upward = increase; downward = decrease). Abbreviations: ADAS-Cog: Alzheimer’s Disease Assessment Scale; ADL: Activities of Daily Living; BNT: Boston Naming Test; BTACT: Brief Test of Adult Cognition by Telephone; CASP-12: Control, Autonomy, Self-realization and Pleasure Scale; CES-D: the 8-item Center for Epidemiologic Studies Depression Scale; CFA: Category Fluency Animals test; CMCV: Choice Motor Coefficient of Variation; CMS: Choice Motor Speed; CR: Cognitive Reserve; CSID: Community Screening Instrument for Dementia; CS-IR/DR: Craft Story immediate and delayed recall; DASS-21: Depression Anxiety Stress Scales; DS: Digit Span; EMA: Ecological Momentary Assessment; ERR: Emotion Expression Recognition; EURO-D: European Depression Scale; F: Female; FCSRT: the Free and Cued Selective Reminding Test; FR: Face Recognition; GDS: Geriatric Depression Scale; HT: Hotel Task; IADL: Instrumental Activities of Daily Living; ICSS: Inclusion of Community in the Self Scale; IU: Indiana University; JLO: Judgment of Line Orientation; LA: Leisure Activities; LM-D: Logical Memory—delayed recall; LEQ: Lifetime of Experiences Questionnaire; LM-I: Logical Memory—immediate recall; LSNS: Lubben Social Network Scale; LTQ: Leisure Travel Questionnaire; M: Mean; MMSE: Mini-Mental State Examination; MoCA: Montreal Cognitive Assessment; NACC UDS V3: National Alzheimer’s Coordinating Center—Uniform Data Set, Version 3; NART-15: National Adult Reading Test; NIH: National Institutes of Health; NIH-EB: NIH-Toolbox Emotion Battery; NOSQ: Neutral Objects Satisfaction Questionnaire; OHQ: Oxford Happiness Questionnaire; OPQOL: Older People’s Quality of Life—brief; PC: Proverb Comprehension; PPP: Picture-Picture Priming; PSS: Perceived Stress Scale; RCT: Randomized Controlled Trial; SC: Sentence Comprehension; SDMT: Symbol Digit Modalities Test; SPM-16: Standard Progressive Matrices (16-item short form); SR: Story recall; SSRS: Social Support Rating Scale; STW: Spot The Word; SWLS: Satisfaction with Life Scale; TICS: Telephone Interview for Cognitive Status Survey; TIL: Three-Item Loneliness Scale; TMT Trail Making Test; UCLA: University of California, Los Angeles; UCL: Museum Wellbeing Toolkit (12-item); VF: Verbal Fluency; VPA: Visual Paired Associates; VSTM: Visual Short-Term Memory; WEMWBS: Warwick–Edinburgh Mental Well-being Scale; WLM: Word List Memory; WMS-R: Weschler Memory Scale-Revised.

### 3.3. The Effects of Physical Leisure Activities

Several studies have documented the cognitive and emotional effects associated with participating in physically stimulating activities. In the context of such activities, physically stimulating activities that promote cognitive and emotional effects comprise a diverse group of practices that differ in terms of potentially strenuous, light, moderate, and mixed activity levels. Such activities include: walking (outdoors or indoors), swimming, cycling, climbing stairs, performing household activities, mobility exercises (stretching), running, playing volleyball, dancing, practising martial arts, playing table tennis, practising yoga, muscle-strengthening activities, skiing and playing golf. In order to enhance clarity, the extant evidence is presented by means of the grouping of studies according to the primary type of physical activity involved.

We included 29 studies referring to physical leisure activities. 34% (10 out of 29) were observational studies (including cross-sectional and longitudinal design), while the remaining 66% were experimental studies (including randomized–controlled trials and non-randomized studies). Among the experimental studies, 89% assessed outcomes immediately post-intervention (acute outcomes), whereas 11% evaluated outcomes in the long term (see Table 5 for further details).

#### 3.3.1. Walking and General Land-Based Physical Activities

A number of studies have been conducted to investigate the cognitive and emotional effects of aerobic activities performed on land.

Kettinen et al. [113] conducted a five-day crossover study on older adults (≥60 years), in which the subjects practiced 18 holes of golf, 6 km of walking, and 6 km of Nordic walking. The investigation revealed an effect of all three light aerobic exercises on cognitive functions (Table 5). Moreover, the analysis indicated that Nordic walking and conventional walking had more impact than golf on executive functions (Table 5). No significant effects were observed on the brain-derived neurotrophic factor (BDNF) and cathepsin B (CTSB) markers; however, a lower HR was detected in the golf group (Figure 3). In a similar vein, Torre et al. [114] conducted a study on an eight-week Nordic walking intervention, implemented in a natural park environment characterised by varied terrain. The older adult (age range: 65–80 years) participants engaged in three 75 min sessions on a weekly basis, integrating walking with spatial orientation demands, motor coordination, and postural control. HR monitoring was utilized to regulate the intensity of the exercise. The results demonstrated enhancements in visuospatial abilities (Table 5), alongside a significant reduction in HR recovery (HRR) (Figure 3). Loprinzi and Kane [115] conducted a study on young adults (average age: 21.4 ± 2.1). The present domain of study pertains to acute and moderate physical activities, encompassing varying levels of intensity, namely: no exercise (control), low intensity, moderate intensity and high intensity. Each participant completed an acute 30 min treadmill exercise, during which HR and perceived exertion were monitored, prior to performing a series of cognitive tests. The cognitive results demonstrated that acute moderate-intensity exercise significantly improved concentration and attention (Table 5). Furthermore, an inverse relationship between sedentary behaviour and cognitive performance was observed, suggesting that prolonged periods of sedentary behaviour may result in reduced cognitive performance. From a neurobiological perspective, an improvement in HR was also observed (Figure 3). In 2017, Singh [116] conducted a study to ascertain the correlation between moderate leisure-time physical activity and emotional intelligence in a sample of young adult women (age range: 20–30 years). Participants were stratified according to their levels of leisure-time physical activity, incorporating activities performed on a daily basis. The findings indicate that an increase in leisure-time physical activity is associated with higher levels of overall emotional intelligence. As shown in Table 5, there is a demonstrable correlation between physical activity and enhanced self-awareness, self-motivation, emotional stability, empathy and interpersonal relationship management skills. The observational study by Potoczny et al. [117] examined the association between regular physical activity and psychological well-being in a sample of young adults (average age: 20.7 ± 2.92). The physical activity levels of the participants included various forms of aerobic exercise, with a weekly volume of 150–300 min of moderate activity, 75–150 min of vigorous activity, or an equivalent combination. The findings suggest that regular physical activity is associated with greater life satisfaction, with this effect being mediated by increased self-control and more frequent use of adaptive emotional regulation strategies. Furthermore, increased levels of physical activity have been associated with positive emotional states, reduced perceived stress, and fewer depressive symptoms (Table 5). The intensity of physical activity has been demonstrated to correlate positively with psychological well-being and positive affect, and negatively with depression (Table 5). Yang et al. [118] conducted a study on the relationship between moderate physical activity during leisure time and cognitive function in a population of middle-aged and older adults (age range: 60–80 years). The findings indicated a positive correlation between moderate physical activity during leisure time and higher cognitive scores. In particular, research indicates that older adults who engage in physical activity three to four times per week demonstrate a reduced likelihood of memory decline (Table 5).

#### 3.3.2. Water-Based Physical Activity

Canton-Martinez et al. [119] conducted a study to investigate the impact of aquatic-based exercise in comparison to traditional land-based exercise regimens on various cognitive domains in elderly individuals (average age: 65.0 ± 8.8). The participants engaged in a structured training programme, which was conducted three times per week and comprised aerobic and resistance exercises. In the aquatic group, the activities were performed in water at waist or chest level, while the land group performed exercises equivalent in type, duration, and load to ensure comparability between the interventions. Following the conclusion of the intervention, both groups demonstrated enhancements in verbal memory (Table 5). No substantial alterations were observed in executive functions or verbal fluency. With regard to visuospatial skills and visual memory, aquatic exercise was associated with improvements in visual reproduction and both immediate and delayed memory (Table 5). In contrast, land-based exercise produced more limited improvements, which were observed in visual reproduction and delayed memory. So et al. [120] conducted a study on middle-aged and elderly adults (age range: 60–80 years), comparing Aquatic Treadmill Exercise (ATM) and Land Treadmill exercise (LTM). Participants performed incremental tests on an aquatic or land treadmill, followed by a single session of moderate-intensity exercise. The exercise conditions were standardized: during aquatic treadmill exercise, the water depth reached chest level and the water temperature was maintained at 28 °C (ATM), while during land treadmill exercise, the ambient air temperature was set to 23 °C (LTM). Moderate-intensity ATM has been shown to specifically enhance the dual-tracking component of working memory, while LTM has been observed to primarily improve the memorisation component. With regard to overall cognitive functioning, both the ATM and LTM groups demonstrated statistically significant improvement (Table 5). This study provides preliminary evidence that a single session of moderate-intensity exercise, whether in water or on land, can improve cognitive function and working memory (Table 5), as well as cerebral blood flow (CBF) in adults (Figure 3). Terra de Oliveira et al. [121] conducted a study on a sample of elderly people (average age: 68.1 ± 5.5). The participants were enrolled in a 3-month group aquatic exercise programme, which was carried out twice a week in a swimming pool. The intervention incorporated elements of aerobic exercise, resistance training, and coordination exercises, in conjunction with cognitive challenges such as naming, counting in reverse, and identifying shapes and colours. This configuration of the intervention resulted in a cognitive-motor physical activity. The control group maintained their habitual daily activities without increasing their levels of exercise. The findings indicated that engagement in the aquatic exercise programme was associated with substantial enhancements in cognitive function, particularly in the domains of abstract reasoning and executive processes (Table 5). Conversely, cognitive performance demonstrated stability within the control group. Finally, Abou-Dest et al. [122] conducted a study of a population of young adults (age range: 18–30 years) and older adults (age range: 65–80 years), examining the habits of regular swimmers and sedentary individuals. The findings indicated that young adults exhibited significantly superior performance in all cognitive domains examined when compared with older adults. In older adults, regular swimming has been shown to be associated with enhanced performance on three cognitive parameters: behavioural inhibition, working memory, and cognitive shifting (Table 5).

#### 3.3.3. Outdoor Activities

Niedermeier et al. [123] conducted a crossover study on middle-aged adults (mean age = 32 years), comparing three hours of outdoor hiking, indoor treadmill walking, and sedentary control. As illustrated in Table 5, participation in outdoor mountain hiking was associated with a significant increase in positive affective states (e.g., pleasure, activation, elation, calmness) and a reduction in negative states (e.g., fatigue, anxiety) in comparison with the sedentary control group. A comparison of the affective responses elicited by outdoor hiking and indoor treadmill exercise revealed that the former elicited a significantly greater increase in positive emotions, accompanied by enhanced activation and reduced fatigue levels immediately after the activity. HR monitoring showed an increase during outdoor mountain hiking (Figure 3). Krautgasser et al. [124] conducted a study on a group of older adults (≥50 years), who participated in 3.5 h of recreational alpine skiing. The investigation found that recreational alpine skiing in older adults generates positive psychological effects, supporting emotional well-being with increased happiness and sociability (Table 5). Furthermore, an increase in HR was observed during challenging descents (Figure 3). De Brito et al. [125] conducted a 9-week crossover trial in which all participants (age range: 35–59 years) were exposed to two walking conditions: walking in green environments and walking in suburban settings. The first 12 subjects undertook green walking, followed by suburban walking, while the subsequent 12 subjects experienced the reverse sequence, with a two-week washout period between the two phases. Each condition comprised walking sessions of 50 min once a week for three consecutive weeks. Cognitive and emotional assessments were conducted prior to and following each walking session. The findings indicated that exposure to green environments was associated with substantial enhancements in emotional well-being, characterised by a decline in anxiety levels, an augmentation in positive affect, and a reduction in negative affect (Table 5). Conversely, suburban walking did not elicit significant changes in mood. The present study exclusively compared green and suburban walking, with no additional control conditions. With regard to cognitive outcomes, no significant improvements in directed attention were observed after either walking condition, despite the emotional benefits associated with green walking.

#### 3.3.4. Yoga-Based Mind–Body Practices

Datta et al. [126] conducted a study on a sample of young adults (age range: 21–30 years) with the aim of evaluating the effects of yoga nidra practice on cognitive functioning and sleep parameters. The experimental intervention comprised a daily guided yoga nidra practice, lasting 20 min, which incorporated body awareness, attention to breathing, visualisation and deep relaxation. This intervention was conducted over a period of one to two weeks. The results demonstrated that yoga nidra practice was associated with enhancements in cognitive performance, particularly a reduction in reaction times, without compromising accuracy, indicating an increase in processing speed (Table 5). As illustrated in Table 5, certain domains demonstrated an enhancement in accuracy, encompassing aspects such as working memory, learning processes, visual memory, abstraction and conceptualisation, and emotional recognition. Concurrently, enhancements in sleep quality were observed, including an increase in sleep efficiency (Figure 3). Another study on yoga is that of Brunner et al. [127], who examined a sample of young people, middle-aged adults, and older adults (age range: 18–80 years) involved in a Hatha yoga programme. The programme encompassed a series of postures (asanas), breathing exercises (pranayama), and concise mindfulness meditation sessions, each spanning 60 min and occurring over a duration of three or six weeks. The findings of the study demonstrated that participation in the yoga programme was associated with a significant enhancement in working memory, as indicated by improved performance in information retention and manipulation tasks, and an increase in mindful attention (Table 5). The effects manifested to a greater extent in participants with prior experience of yoga. Welford et al. [128] conducted a study on a sample of physically inactive older adults (age range: 65–85 years), who were assigned to either a mind–body practice (Hatha yoga, including postures, breathing exercises, and relaxation) with light-to-moderate intensity group aerobic activities (cycling, dance exercise, and synchronized aerobics) or a control group. The duration of both interventions was 12 weeks, encompassing 60 min sessions on multiple occasions throughout the week. The findings indicated that engagement in both the yoga and aerobic exercise programmes was associated with an enhancement in phonological verbal fluency in comparison to the control group (Table 5).

#### 3.3.5. Resistance and Strength-Based Activities

Yoon et al. [129] conducted an experimental study on a sample of older adults (average age: 73.94 ± 4.27). Participants were assigned to either a high-speed resistance training (HSRT) group that utilised resistance bands or a control group that performed balance and stretching exercises using resistance bands. The intervention was conducted over a period of 16 weeks, encompassing training sessions that were held three times a week and each of which lasted approximately one hour. Each session comprised a 10 min warm-up, followed by 40 min of high-speed resistance exercises, including seated rowing, one-leg press, applied pec deck fly, seated leg raise, lateral raises, semi-squats, wide squats, and bridge, concluding with a 10 min cool-down period. It is imperative to note that all sessions were subject to direct supervision by an instructor, a measure implemented to ensure safety and adherence to the prescribed guidelines. It is also noteworthy that a minimum interval of 48 h was observed between sessions to ensure comprehensive recovery and prevent any undue strain on the subjects. The results indicated that HSRT significantly improved processing speed and executive function after the 16-week intervention (Table 5). Mateluna-Núñez et al. [130] conducted a randomised controlled clinical trial in young adults (age range: 18–28 years). The study examined the effects of free weight resistance training on cognitive performance related to text processing. Participants in the experimental group undertook a 10-week programme of free-weight resistance training, comprising three training sessions per week. The cognitive outcomes of the study were focused on text processing performance, and these outcomes were assessed using eye-tracking (ET) technology. To elaborate, the primary objective of the study was to measure first-pass reading times (FPRTs) and total reading times (TRTs) during reading tasks of varying syntactic complexity. The training programme was found to enhance participants’ ability to focus on complex syntactic structures, while concomitantly reducing their reading times for texts of low complexity (Table 5).

#### 3.3.6. Open-Skill and Cognitively Engaging Sports

Chiu et al. [131] conducted a study on young adults, comparing sedentary individuals (average age: 21.75 ± 0.70), participants in regular physical exercise (e.g., running and swimming) (average age: 21.50 ± 0.58), and individuals engaged in a skill-based sport, specifically volleyball (average age: 23.36 ± 0.53). The findings indicated that volleyball players exhibited superior performance in terms of attention, characterised by enhanced accuracy and expedited response times in the flanker task when compared to both the control group and the general exercise group (Table 5). The exercise group demonstrated intermediate effects. Schaefer et al. [132] conducted a study on young adults, comparing experienced table tennis players (average age: 25.0 ± 5.01) with beginners (average age: 28.13 ± 12.3). Participants were tasked with performing table tennis manoeuvres (serves and ball returns) in conjunction with cognitive tasks of working memory and attention (3-back and backward counting), thereby creating a cognitive-motor dual-task situation. The findings indicated that the experts demonstrated superior cognitive-motor integration, exhibiting significantly reduced dual-task costs in comparison to the novices (Table 5). Hola et al. [133] conducted an experimental study on independent older adults (age range: 65–80 years). The domain of study was physical activity, with two forms of intervention: dance and martial arts, in comparison with a control group. The interventions were administered on a biweekly basis, with each session lasting 90 min, for a period of 12 weeks. These interventions were meticulously designed to address the specific requirements of older adults, with a primary focus on inducing global muscle activation and ensuring cognitive and motor involvement. The cognitive results demonstrated that the dance group exhibited significant enhancements in verbal and learning memory, short-term memory, semantic memory, processing speed, attention, and cognitive flexibility (Table 5) in comparison to the control group. As demonstrated in Table 5, the dance group demonstrated enhanced emotional well-being. Furthermore, both intervention groups (dance and martial arts) demonstrated significant increases in peripheral concentration of BDNF levels (Figure 3), a neurobiological indicator of brain plasticity, while the control group exhibited a decrease. The study by Russo et al. [134] examined the association between the type of sport and specific cognitive abilities in a sample of young adults and middle-aged adults (age range: 20–55 years). Participants were divided into three groups: open-skill athletes, closed-skill athletes and non-athlete controls. Open-skill sports (for example, tennis, football, volleyball, rugby, martial arts and multisports) are characterised by dynamic and unpredictable contexts that require continuous perceptual and decision-making adaptation. Conversely, closed-skill sports (for example, swimming, artistic gymnastics, yoga, climbing, bodybuilding and athletics) are characterised by their occurrence in more stable and predictable environments, with reduced demands for real-time cognitive processing. All participants engaged in regular sporting activities and underwent a series of cognitive assessments. The findings of the present study demonstrate that open-skill athletes demonstrate superior performance in visual search skills in comparison to closed-skill athletes (Table 5).

#### 3.3.7. Cycling and Other High-Intensity Activities

Leyland et al. [135] conducted a study on a sample of older adults (age range: 50–83 years), categorised into three groups: non-cyclists, traditional cyclists, and e-bike cyclists. The athletes engaged in this sport for a minimum of three times per week, with each session lasting 30 min, for a period of eight weeks. Improvements in executive functions, specifically letter updating and inhibition, were observed in both groups of cyclists in comparison to the control group (Table 5). An enhancement in processing speed was only observed among the e-bike participants (Table 5). Evidence suggests a tendency towards enhancement in psychological well-being; however, these observations did not attain statistical significance. In the study conducted by Stenling et al. [136], a group of young adults (age range: 18–24 years) who engaged in strenuous physical activity (i.e., stair-climbing interval training) were examined. The protocol specifically incorporated stair climbing intervals: the climbing routine was performed for a duration of 1 min, followed by a 1 min recovery period. This sequence was repeated for a total of 3 min, inclusive of a warm-up and cool-down phase, resulting in a total duration of approximately 10 min. With regard to the cognitive domain, an enhancement in task-switching aptitude was observed among males, a phenomenon that was concomitant with the intensity of the ascent (Table 5). No significant effects were observed in the female subjects. The emotional effects of the treatment were characterised by an increase in energy levels, a decrease in tension and fatigue, and an improvement in positive moods (Table 5). From a neurobiological standpoint, an enhancement in HR was also observed (Figure 3). Another study by Oberste et al. [137] on young adults (≥18 years) included mixed physical activities alongside cycling. For the experimental phase of the study, participants were divided into three groups: an aerobic exercise group, who performed 30 min of moderate-intensity cycling on a cycle ergometer; an active control group, who performed 30 min of lower limb stretching with minimal cardiovascular exertion; and a passive control group, who spent 30 min watching a sitcom. During the aerobic exercise session, HR was monitored. The findings indicate that all groups demonstrated enhancements from the pre- to the post-intervention evaluation (Figure 3), suggesting the potential for a practice- or general activation-related effect. However, the aerobic exercise group demonstrated significantly greater improvements in cognitive flexibility than the active control group, accompanied by enhanced mood, reduced fatigue, increased motivation, and elevated self-perception of cognitive performance (Table 5). Finally, Raichlen et al. [138] conducted a study on a sample of healthy older adults (age range: 60–74 years) living in the community. The present study compared two distinct groups: the first group engaged in aerobic exercise, while the second group underwent cognitive training. The third group underwent a simultaneous combination of aerobic exercise and cognitive training. These two groups were then compared with a passive control group. The physical activity comprised aerobic exercise on a recumbent bicycle, performed three times a week for a period of 12 weeks. In contrast, the cognitive training comprised digital exercises targeting executive functions, memory, and processing speed. In the combined condition, both activities were performed simultaneously. The findings indicated that engagement in both aerobic exercise and cognitive training, particularly in the combined exercise-cognition intervention, was associated with substantial enhancements in cognitive performance under dual-task conditions, as evaluated during a walking test with serial subtraction (Table 5).

#### 3.3.8. Everyday-Life Physical Activity and Domestic Work

Feldberg et al. [139] conducted a study to ascertain the impact of occupational complexity and domestic tasks on cognitive function in the elderly population (≥60 years). The primary objective of the study was to assess the association between involvement in housework of varying complexity and three specific cognitive domains. The physical activities considered in this study were primarily household chores, which were then classified according to their level of complexity. The activities were categorised as follows: simple activities (e.g., reading labels or preparing a drink), intermediate activities (e.g., using appliances and managing communications), and complex activities (e.g., home repairs and financial negotiations). The results indicate that housework, both light and more demanding, with mild to moderate intensity, is associated with certain aspects of cognitive functioning, particularly logical memory, working memory, and specific components of executive functions (Table 5). The study by Lee et al. [140] analysed the association between housework and cognitive functioning in a sample of adults, divided into two groups: middle-aged adults (age range: 21–64 years) and older adults (age range: 65–90 years). The primary focus of the study pertained to the comparison of light and heavy housework. The findings indicate that, in older adults, higher levels of engagement in domestic tasks are associated with superior overall cognitive function, superior performance on attentional tasks, and enhanced memory functioning (Table 5). Specifically, the combination of light (LH) and heavy housework (HH) has been shown to be associated with more favourable cognitive outcomes. In their 2025 study, Wang et al. [141] examined the association between the frequency of housework and the pattern of cognitive decline in a large sample of older adults (≥65 years). The frequency of housework was monitored between 2008 and 2010 and classified into four trajectories: consistently high, low to high, high to low, and consistently low. Cognitive function was then assessed longitudinally from 2010 to 2018, allowing for an analysis of cognitive evolution over approximately ten years. The findings indicate that a decline in the frequency of domestic tasks (from high to low) or the maintenance of consistently low levels was associated with accelerated cognitive decline over time. Conversely, older adults who demonstrated a consistently high frequency of housework or who augmented their engagement from initially low levels exhibited superior overall cognitive function (Table 5).

**Table 5:** Effects of physical-based leisure activities on cognitive functioning, emotional well-being, and neurobiological/neurophysiological outcomes.

Reference	Population	Sample Size (*n*)	Sex	Age Range (Years)	Average Age (Years)	Study	CR Measured by Questionnaire	LA Measured by Questionnaire	Specific Activity	Frequency/Duration	Cognitive or Emotional Outcome	Cognitive or Emotional Function Measure	Outcome Assessment Timing	Acute/Long-Term Effect	Improvement	Neurophysiological/Neurobiological Outcome
***Walking and general land-based physical activities***																
Kettinen et al., 2023 [113]	Older adults	25	F: 36%	≥60 years	69 ± 4	Randomized crossover trial	No	No	Golf round and walking	5-day protocol comparing single bouts of 3 exercise modalities	Cognitive: visuospatial lower and higher cognitive function	TMT	Pre-exercise and post-exercise for each trial session	Acute effect	↑ visuospatial lower ↑ higher cognitive function	Yes: HR, BDNF and CTSB
Torre et al., 2024 [114]	Older adults	14	F: 71.4%	65–80	71.1 ± 3.7	Single-arm prospective pilot intervention study	No	No	Nordic walking	8 weeks (75 min/session, 3 days/week)	Cognitive: executive functions- inhibition function, information processing speed, cognitive switching, visuospatial capacities.	MoCA, CWST, TMT, RCFCT.	Evaluated at 3 timepoints: baseline (pre-test), 4 weeks (intermediate test), and 8 weeks (post-test)	Acute effect	↑ visuospatial capacities	Yes: HR
Loprinzi and Kane, 2015 [115]	Young adults	87	F: 41.3%	-	21.4 ± 2.1	Counterbalanced crossover RCT	No	IPAQ [142]	Treadmill	Single 30 min acute bout + 1-week accelerometry/1-week washout	Cognitive and emotional: cognitive visual attention and task switching, memory, reasoning, concentration, planning and affect	TMT, Spatial Span and Paired Associates, Grammatical Reasoning and Odd One Out, Feature Match Polygon, Spatial Search and Spatial Slider, MSQ	Post-exercise	Acute effect	↑ concentration ↑ visual attention	Yes: HR
Singh, 2017 [116]	Young adults	126	F: 100%	20–30	-	Cross-sectional study	No	In-house measure	Leisure time physical activity	-	Emotional: self-awareness, empathy, self-motivation, emotional stability, managing relations, Integrity, self-development, value orientation, commitment, altruistic behavior, overall emotional intelligence	Emotional Intelligence Scale (EIS; 5-point scale)		-	↑ self-awareness ↑self-motivation ↑emotional stability ↑ managing relations integrity ↑self-development ↑value orientation ↑commitment.	No
Potoczny et al., 2025 [117]	Young adults	186	F: 91%	-	20.7 ± 2.92	Cross-sectional survey study	No	In-house measure	Aerobic activity	-	Cognitive and emotional: self-control, subjective well-being, emotion regulation	SWLS, BSCS, ERQ	-	-	↑ life satisfaction ↑ self-control ↑ emotion regulation ↑ well-being ↓ stress ↓ depression	No
Yang et al., 2024 [118]	Older adults	2657	F: 48.14%	60–80	-	Cross-sectional population-based study	No	GPAQ [143]	Leisure time physical activity	-	Cognitive: immediate and delayed learning ability for new language information, categorical language fluency, processing speed, sustained attention, working memory, subjective cognitive function, assessing self-perceived memory	CERAD-WL, CERAD-DR, AF, DS, family interview	-	-	↑ memory	No
***Water-based physical activity***																
Canton-Martinez et al., 2025 [119]	Older adults	16	F: 93.75%	-	65.0 ± 8.8	Controlled clinical trial (two parallel groups)	No	No	Acquatic excercise/land excercise	4 weeks	Cognitive: general cognitive ability (GCA), learning and verbal memory, Inhibitory control, cognitive flexibility, verbal fluency, visual memory.	MoCA, RAVLT, FDT, COWAT, ROCF	Baseline and post-intervention (4 weeks)	Acute effect	↑ verbal memory ↑ visuospatial function ↑ visual memory	No
So et al., 2024 [120]	Older adults	28	F: 32.14%	60–80	ATM: 65.9 ± 0.8 LTM: 65.9 ± 1.0	Randomized parallel-group trial	No	IPAQ [142]	Aquatic Treadmill Exercise	Single bout of moderate-intensity	Cognitive: General cognitive performance and working memory	DS	Pre-exercise (baseline) and immediately post-exercise	Acute effect	↑ general cognitive performance ↑ working memory	Yes: cerebral blood flow and VO2max
Terra de Oliveira et al., 2024 [121]	Older adults	31	F: 54.8%	-	68.1 ± 5.5	Prospective single-blinded controlled trial	No	No	Aquatic exercise	3 months (2 times/week)	Cognitive: abstract reasoning; speed and accuracy in interpreting information; problem-solving.	Raven’s Progressive Matrices; Wisconsin Card Sorting Test.	Baseline and 12-week re-evaluation	Acute effect	↑ problem-solving ↑ accuracy in interpreting information	No
Abou-Dest et al., 2012 [122]	Young adults and older adults	48	F: 43.75%	65–80; 18–30	23.56 ± 2.56 69.13 ± 3.88 69.25 ± 3.25	Cross-sectional comparative study	No	DPAS [144]	Swimming	-	Cognitive: processing speed, executive functions, inhibition, updating, shifting	SRT, CRT, RNG, The Verbal Running Span Task, The Spatial Running Span Task, Dimension-Switching Task, Stimulus-Response Compatibility Switching Task, WCST	-	-	↑ behavioral inhibition ↑ working memory ↑ shifting	No
***Outdoor activities***																
Niedermeier et al., 2017 [123]	Middle-aged adults	42	F: 48%	-	M = 32	Randomized crossover trial	No	In-house measure	Mountain hiking and indoor treadmill	Single 3 h intervention per condition	Emotional: affective valence (pleasure), perceived activation (high or low), anxiety, calmness, fatigue, or anger are distinguished, perceived effort.	FS, FAS,, MSS, STAI, RPE.	Pre-intervention (T1) and post-intervention (T5) per condition	Acute effect	↑ pleasure ↑ perceived activation ↑ calmness ↓ anxiety	Yes: HR
Krautgasser et al., 2006 [124]	Older adults	49	F: 32.7%	>50 years	63 ± 6	Acute field-based repeated-measures observational study	No	In-house measure	Alpine Skiing	Single 3.5 h session	Emotional: individual mind-set that would reflect the physical sensations of skiing (readiness for the activity, sociability, self-confidence, happiness, nervousness, fatigue or exhaustion, anger, worry, and concentration)	Hackfort and Schlattmann questionnaire	Evaluated at 3 timepoints during/after skiing: 0.5 h (T1), 1.5–2 h (T2), 3.5 h (T3)	Acute effect	↑ individual mind-set ↑ happiness ↑ sociability	Yes: HR
de Brito et al., 2019 [125]	Middle-age adults	23	F: 87%	35–59	49.3 ± 6.7	Pilot crossover trial	No	No	Walking	2 single 50 min walking sessions (Green vs. Suburban)	Cognitive and emotional: directed-attention anxiety and mood	DS STAI-S, PANAS	Pre-walk and post-walk for each environmental condition	Acute effect	↓ anxiety ↑ positive affect ↓ negative affect	No
***Yoga-based mind–body practices***																
Datta et al., 2023 [126]	Young adults	41	F:0%	21–30	25.15 ± 2.27	Pre-post repeated measures pilot study	No	No	Yoga nidra	2 weeks (Yoga Nidra practice following 5 training sessions)	Cognitive and emotional: sensory motor speed, spatial learning and memory, working memory, abstraction and concept information, spatial orientation, abstract reasoning, complex scanning and visual tracking, risk decision making, and vigilant attention, emotion identification	MPT, VOLT, NBACK, AIM, LOT, MRT, DS, BART, PVT:10 min. ERT	Baseline, 1-week, and 2-week follow-up (tested at 3 daily timepoints)	Long-term effect (2-week follow-up)	↑ reaction time ↑ processing speed ↑ accuracy ↑ abstraction ↑concept formation ↑ working memory ↑ accuracy for happy, fear and anger	Yes: Power spectral density (PSD) of EEG daytime recording
Brunner et al., 2017 [127]	Young adults middle-aged and older adults	43	F: 81.4%	18–80	24.77 ± 10.72	Pre-post single-arm trial	No	No	Hatha yoga	6 weeks	Cognitive: working memory; attention, receptive awareness	WAIS DS, LNS, MAAS.	Baseline (pre-intervention) and post-intervention	Acute effect	↑ working memory ↑ attention	No
Welford et al., 2023 [128]	Older adults	82	F: 76.8%	65–85	YI: 72.1 AI: 73.3 CG: 72.1	RCT	No	IPAQ [142]	Yoga and aerobic exercise	12 weeks	Cognitive: verbal fluency	FAS verbal fluency; Animals; Verbs	Baseline and 12-week follow-up	Long-term effect (12-week follow-up)	↑ verbal fluency	No
***Resistance and strength-based activities***																
Yoon et al., 2018 [129]	Older adults	43	F: 69.8%	-	73.94 ± 4.27	RCT	No	In-house measure	Muscle strength exercises and stretching	16 weeks (3 times/week)	Cognitive: verbal fluency, language domain, memory domain, delayed recall, recognition, visuospatial domain	Rey 15-Item memory test, TMT, DS, FAB	Baseline, 8 weeks, and 16 weeks	Acute effect	↑ processing speed ↑ executive function	No
Mateluna-Núñezet al., 2025 [130]	Young adults	18	F: 83.3%	19–28	21.0 ± 2.14	Randomized double-blind clinical trial	No	No	Muscle strength exercises	10 weeks	Cognitive: text processing tasks [FPRTs, TRTs]	ETtechnology	Baseline (pre-intervention) and post-intervention (10 weeks)	Acute effect	↑ processing	No
***Open-skill and cognitively engaging sports***																
Chiu et al., 2017 [131]	Young adults	31	F: 51.6%	-	CG: 21.75 ± 0.70 EXE: 21.50 ± 0.58 V: 23.36 ± 0.53	Cross-sectional comparative study	No	No	Running, swimming, volleyball	-	Cognitive: reaction time and accuracy	Flanker test	-	-	Volleyball: ↑ reaction time ↑ accuracy	No
Schaefer et al., 2022 [132]	Young adults	16	F: 37.5%	-	EXP: 25.0 ± 5.01 NOV: 28.13 ± 12.3	Cross-sectional study	No	In-house measure	Table tennis	-	Cognitive: executive function and attention	3-back and Counting Backwards in steps of 7	-	-	↑ executive function ↑ attention	No
Hola et al., 2024 [133]	Older adults	77	F: 83.1%	65–80	70.3 ± 3.8	RCT	No	RAPA [145]	Dance and martial arts	12 weeks	Cognitive: verbal memory, learning abilities, immediate short-term memory, semantic memory, processing speed, attention, mental flexibility, mood.	RAVLT, the phonemic fluency test of words with the initial letter “P”, the animal fluency test, DS of WAIS TMT, GDS, FAQ-CZ.	Baseline (pre-intervention) and post-intervention	Acute effect	↑ verbal memory ↑ learning abilities ↑ immediate ↑ short-term memory ↑ semantic memory ↑ processing speed ↑ attention ↑ mental flexibility ↑ mood	Yes: BDNF
Russo et al., 2022 [134]	Young and middle-aged adults	95	F: 34.73%	20–55	28.62 ± 8.39	Cross-sectional comparative study	No	In-house measure	Open and closed-skill sports	-	Cognitive: visual search ability, visual working memory, and reasoning	Visual Search Task, Corsi Block Tapping—Backwards Task, Cognitive Reflection Task	-	-	↑ visual search	No
***Cycling and other high-intensity activities***																
Leyland et al., 2019 [135]	Older adults	100	F: 59%	50–83	Non-cycling Controls: 66.04 ± 8.84 Pedal Cyclists: 63.03 ± 7.47 E-bike Cyclists: 61.90 ± 7.00	Mixed-methods intervention study	No	PASE [146]	Outdoor cycling	8 weeks	Cognitive and emotional: verbal fluency, task switching, working memory and updating, inhibition, immediate and delayed memory recall, spatial function, autonomy, environmental mastery, personal growth, positive relations with others, purpose in life, and self-acceptance, life satisfaction, positive and negative Affect.	VF, Plus-minus task, Letter updating task, Stroop task, Stop-it task, Eriksen flanker task, CERAD, PWB, SL, PANAS.	Pre-interventionand post-intervention	Acute effect	↑ executive function ↑ speed of processing ↑ inhibition ↑ updating ↑ well-being	No
Stenling et al., 2019 [136]	Young adults	32	F: 65.62%	18–24	19.4 ± 1.3	Repeated measures crossover study	No	In-house measure	Stair-climbing	Single bout	Cognitive and emotional: visuomotor processing performance, inhibitory control, and switching abilities, sad, energetic, tense, happy, tired, and calm; in that order	Computer-based cognitive battery, VAMS.	Post-exercise assessment per session	Acute effect	↑ switching performance ↑ energetic ↓ tense ↓ tired	Yes: HR
Oberste et al., 2021 [137]	Young adults	99	F: 47.47%	18+	21.61 ± 3.60	Randomized controlled study (3 parallel groups)	No	No	Aerobic exercise	Single 30 min acute bout (post 60 min mental fatigue induction)	Cognitive and emotional: cognitive flexibility performance, mood, tiredness, nervousness, self-perceived cognitive performance capacity, and motivation for cognitively demanding tasks.	TMT, Multidimensional Mood State Questionnaire	Baseline (T-1), post-fatigue (T0), and immediately post-treatment (T1)	Acute effect	↑ well-being ↑ cognitive flexibility ↑ mood ↓ tiredness ↑ motivation ↑ self-perceived cognitive performance	Yes: HR
Raichlen et al., 2020 [138]	Older adults	74	-	60–74	EXCOG: 67.67 ± 4.65 EX: 68.1 ± 3.92 COG: 66.35 ± 3.89 CON: 69.28 ± 4.34	RCT	No	In-house measure	Aerobic exercise training on bicycle	12 weeks (3 days/week)	Cognitive: executive function, memory, and processing speed	Tablet-based cognitive training program, DTWT	Baseline, 6 weeks (mid-point), and 12 weeks (post-intervention)	Acute effect	↑ executive function ↑ memory ↑ processing speed	No
***Everyday-life physical activity and domestic work***																
Feldberg et al., 2024 [139]	Older adults	120	F: 68%	≥60 years	72.34 ± 5.07	Cross-sectional study with path analysis	In-house questionnaire	In-house measure	Housework	-	Cognitive: episodic memory, visual confrontation denomination, verbal fluency, semantic fluency, vocabulary, set-shifting and cognitive flexibility.	Signoret’s memory battery, verbal auditory test spain-complutense, language boston naming test, VF, semantic fluency test, vocabulary subtest WAIS, TMT, digits backwards, analogies subtest WAIS- cubes subtest WAIS-	-	-	↑ logical memory ↑ working memory ↑ executive function	No
Lee et al., 2021 [140]	Young adults and older adults	489	F: 57.06%	21–64 65–90	Young: 44 ± 14 older: 75 ± 6	Cross-sectional study	No	GPAQ [143] and LAPAQ [147]	Housework	-	Cognitive: immediate and delayed memories, visuospatial construction, language and attention	RBANS	-	-	↑ attention ↑ immediate and delayed memories	No
Wang et al., 2025 [141]	Older adults	8141	F: 59.3%	≥65 years	75 ± 6.6	Prospective longitudinal cohort study	No	In-house measure	Housework	-	Cognitive: orientation, executive function, and memory	BC, NPP, RCD, IWR, DWR, S7, ONT.	Baseline (2010) and biennially through 2018 (up to 8 years)	-	↑ executive function	No

Notes: The arrows indicate the direction of change (upward = increase; downward = decrease). Abbreviations: AF: Animal Fluency Test; AI: Aerobic Intervention; AIM: Abstract Matching; ATM: quatic Treadmill exercise; BART: Balloon Analog Risk Task; BC: Backward Counting; BDNF: Brain-Derived Neurotrophic Factor; BSCS: Brief Self-Control Scale; CDT: Clock Drawing Test; CERAD: Consortium to Establish a Registry for Alzheimer’s Disease; CERAD-DR: Consortium to Establish a Registry for Alzheimer’s Disease Recall Module; CERAD-WL: Consortium to Establish a Registry for Alzheimer’s Disease Word Learning; CG: Control Group; CCT: Cube Copying Test; COG: Cognitive Training; CON: Video-Watching Control; COWAT: Controlled Oral Word Association Test; CR: Cognitive Reserve; CRT: Visual 2-Choice Reaction Time Task; CTSB: Cathepsin B; CWST: Color–Word Stroop Test; DS: Digit Span; DPAS: Dijon Physical Activity Score; DTWT: dual-task walking test; DWR: Delayed Word Recall; EEG: Electroencephalogram; EIS: Emotional Intelligence Scale; ERQ: Emotion Regulation Questionnaire; ERT: Emotion Recognition Task; ET: Eye-tracking; EX: aerobic exercise; EXCOG: Combined Aerobic Exercise and Cognitive Training; EXE: Exercise; EXP: Experts; F: Female; FAB: Frontal Assessment Battery; FAQ-CZ: Questionnaire of Functional State; FAS: Felt Arousal Scale; FAS verbal fluency; FDT: Five-Digit Test; FPRTs: First-pass Reading Times; FS: Feeling Scale; GDS: Geriatric Depression Scale; GPAQ: Global Physical Activity Questionnaire; HDS-R: Hasegawa Dementia Score-Revised; IPAQ: International Physical Activity Questionnaire; IWR: Immediate Word Recall; LNS: Letter-Number Sequencing; LA: Leisure Activities; LAPAQ: Longitudinal Ageing Study Amsterdam Physical Activity Questionnaire; LOT: Line Orientation Task; LTM: Land-Based Treadmill exercise; M: Mean; MAAS: Mindful Attention Awareness Scale; MMSE: Mini-Mental State Examination; MoCA: Montreal Cognitive Assessment; MPT: Motor Praxis Task; MRT: Matrix Reasoning Task; MSQ: Mood States Questionnaire; MSS: Mood Survey Scale; NBACK: Fractal-2-back; NOV: Novices; NPP: Naming the President and Vice President; ONT: Objective Naming Test; PANAS: Positive and Negative Affect Schedule; PASE: Physical Activity Scale for the Elderly; PVT: Psychomotor Vigilance Task; PWB: Psychological Well-Being Questionnaire; RAPA: Rapid Assessment of Physical Activity; RAVLT: Rey Auditory Verbal Learning Test; RCD: Recall of the Current Date; RBANS: Repeatable Battery for the Assessment of Neuropsychological Status; RCFCT: Rey Complex Figure Copy Task; RNG: Random Number Generation; RCT: Randomized Controlled Trial; ROCF: Rey–Osterrieth Complex Figure; RPE: Rating of Perceived Exertion; S7: Serial 7’s Test; SBQ: Sedentary Behavior Questionnaire; SL: Satisfaction in Life; SRT: Auditory Simple Reaction Time Test; STAI: State–Trait Anxiety Inventory; STAI-S: State–Trait Anxiety Inventory State subscale; SWLS: Satisfaction with Life Scale; TMT: Trail Making Test; TRTs: Total Reading Times; V: Volleyball; VAMS: 100-point Visual Analogue Mood Scales; VOLT: Visual Object Learning Task; WAIS: Wechsler Adult Intelligence Scale; WCST: Wisconsin Card Sorting Test; YI: Yoga Intervention.

**Figure 3:**
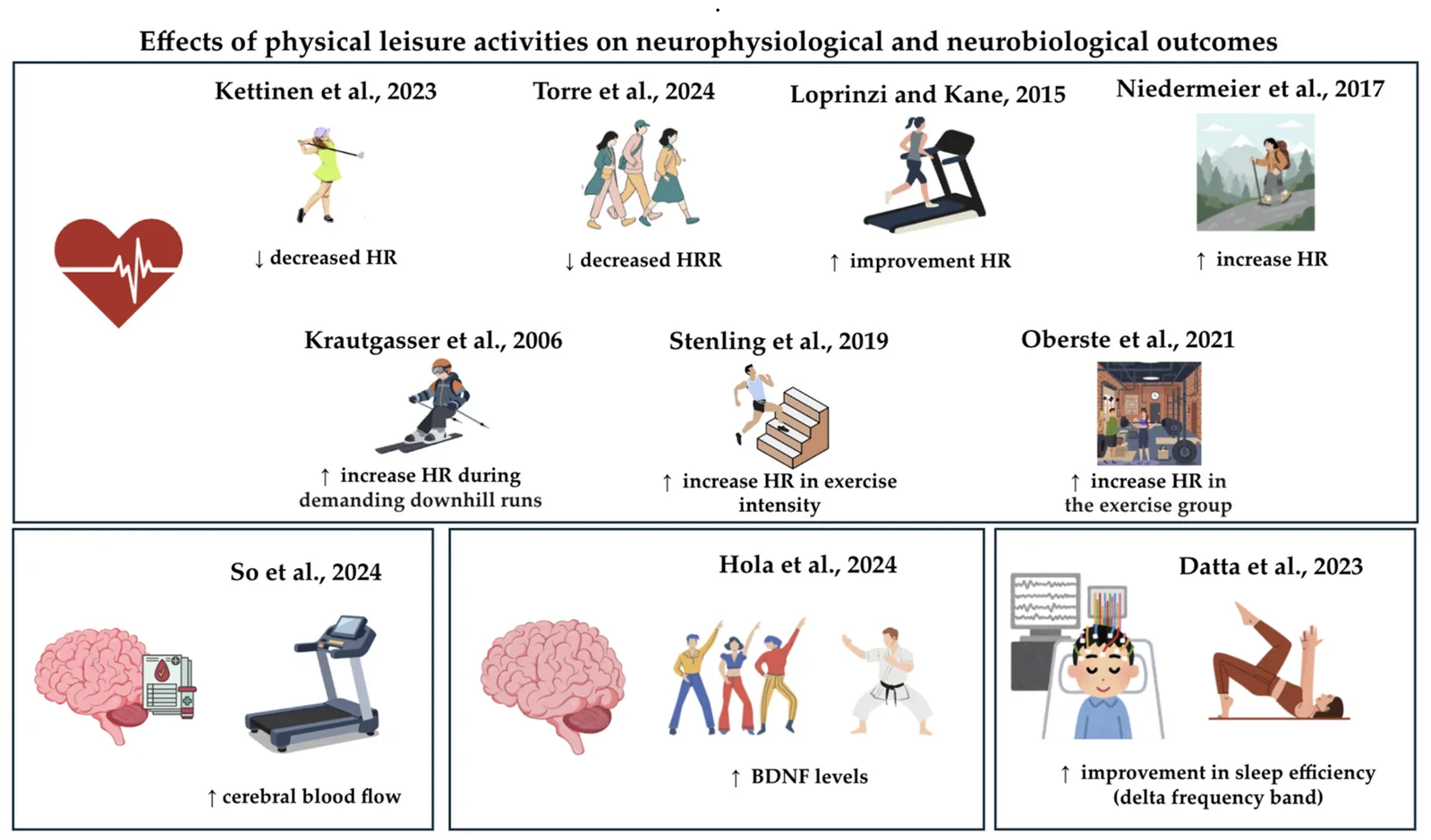
This schematic overview illustrates the impact of physical leisure activities on neurophysiological and neurobiological outcomes. These outcomes were assessed using instrumental techniques (ECG and EEG) and blood sample analysis. The arrows indicate the direction of change (upward = increase; downward = decrease). The upper panel shows that several studies [113,114,115,123,124,136,137] indicate an improvement in HR in all groups performing physical activity, compared to the control groups. In the bottom right panel, Datta and colleagues [126] observed an improvement in sleep efficiency through EEG recording during sleep in a group of participants performing yoga. The final two studies [120,133] on the left demonstrate an increase in BDNF levels and cerebral blood flow through blood sample analysis in activities such as dancing, martial arts and treadmill running.

## 4. Discussion

This narrative review summarises studies on the cognitive and emotional effects of recreational activities in healthy adults, and examines their potential contribution to CR. Across all domains, the findings converge towards a consistent conclusion, namely that regular engagement in cognitively stimulating, socially embedded, and physically active recreational activities is associated with improved cognitive function (attention, memory, executive function, and language) and improved emotional well-being (better emotional regulation, less stress, and less anxiety) during adulthood. In the following sections, the results of the included studies are summarised for each domain and interpreted in light of broader literature, discussing evidence against clinical populations.

### 4.1. Cognitive Leisure Activities

The present review confirms that reading is consistently associated with improved cognition across multiple domains [53,54], in line with Chang et al. [148], who identified reading as a protective factor against cognitive decline in older age. These effects are not limited to cognitive-behavioural changes, but appear to be supported by cerebral functional, structural, and neuroendocrine modifications.

In the case of reading [53], no significant differences in cortical thickness or hippocampal volume emerged between readers and non-readers. However, a positive correlation was observed between years of education and hippocampal volume, indicating that formal schooling is associated with structural brain markers, while reading habits may contribute mainly through functional and network efficiency mechanisms. A resting-state fMRI study showed that the strength of connectivity is associated with reading skills [149]. This is corroborated in writing, which has been shown to benefit working memory, emotional regulation, and life satisfaction [55,56]. This assertion has been corroborated by Guo [150], who found the expressive writing interventions alleviate symptoms of depression, anxiety, and stress in both healthy and subclinical subjects.

Strategic and cognitively demanding games show similar benefits in visuospatial attention, visual working memory, planning, cognitive flexibility, problem solving, and decision making both in healthy individuals [57,58,59], and in prodromal neurodegenerative disease [151,152]. The evidence from other studies supports this concept: Bediou et al. [153] reported benefits in healthy young adult players of action video games for top-down attention and spatial cognition.

The acquisition of a new language has been demonstrated to be associated with improvements in general cognition [60] and in visual working memory [61]. Furthermore, the acquisition of a new language has been linked to heightened functional connectivity in the fronto-parietal regions, specifically the rIFG, the rSFG, and lSPL. These regions play a pivotal role in executive control and working memory [60]. This pattern suggests greater integration between nodes in the cognitive control network, consistent with enhanced neural efficiency. Epidemiological evidence confirms that bilingual individuals show, on average, 3.45 more dementia-free years than monolinguals [154].

Live artistic performances and cultural experiences carry comparable emotional benefits [62]; this converges with independent evidence that recreational activities most strongly affect daily-living functionality [155] and that cultural engagement (museums, cinema, theatre, arts) reduces depression risk [156].

Participatory activities show a similarly broad cognitive and emotional benefit profile in healthy individuals [63,64,65,66,67,68,69]. Moreover, artistic engagement has been associated with reduced cognitive decline both in healthy elderly [157] and in stroke patients [158]. Similarly, Colombo et al. [159] and Fabio et al. [160] reported that leisure activities predicted the level of creative thinking in middle-aged and older adults. Although these results do not resolve the reverse causality question, however, they may indicate an association between leisure activity engagement and higher-order cognitive functions.

Receptive and active musical engagement show consistent cognitive and emotional benefits in healthy adults [70,71,72,73,74,75,76,77] and in different clinical populations [161,162,163]. These results may support the use of more structured interventions based on music practice in clinical populations. As an example, music therapy has been found effective in reducing clinical symptoms in patients with Parkinson’s disease [164]. The results of empirical studies on live artistic experiences [62] have demonstrated increased levels of cognitive absorption, as evidenced by EEG, alongside modulation of HR and electrodermal activation, in response to musical intensity, volume, and speed. In the context of long-term musical training [77], the preservation of verbal working memory has been demonstrated to be associated with greater integrity of the right putamen and more efficient cerebellar functioning. Music listening [70] demonstrated a reduction in salivary cortisol and modulation of alpha-amylase, suggesting a direct effect on the hypothalamic–pituitary–adrenal (HPA) axis and the autonomic nervous system. Recent neurophysiological evidence demonstrates that musical experience activates a substantial neural network associated with emotional processing, encompassing limbic structures such as the amygdala, components of the reward system, and integrated cortical networks that process sensory and emotional signals. Trost et al. [165] conducted a study that observed the impact of live music on neural activity and connectivity. Their findings suggest that musical experiences can modulate aesthetic and emotional responses through neurobiological mechanisms involving the amygdala.

Taken together, although no direct evidence of increased CR has been provided by these kinds of studies, the findings globally suggest that cognitively stimulating recreational activities may support cognitive functioning and emotional well-being.

### 4.2. Social Leisure Activities

In the social domain, activities such as visiting friends and relatives, participating in religious events, volunteering, attending joint artistic and cultural events, travelling, and participating in social and recreational activities have been consistently associated with improvements in cognitive functioning and emotional well-being.

Shared reading recreational activities (such as book clubs) are consistently associated with enhanced cognitive function [83] and emotional well-being [82], a pattern that extends downward in age: shared reading in children aged 2–3 predicts stronger receptive vocabulary and early academic skills [166]. Yaffe et al. [167] found in children comparable attentional and social-bonding benefits from playful interaction.

Group travel experiences show a similar profile, reducing stress and loneliness [86], improving well-being, boosting quality of life [84,85], and enhancing several cognitive domains [86,168].

Visiting friends and relatives is reported to improve general cognition [88,89], episodic memory, and executive functions [87]. Moreover, these activities are known to reduce loneliness, and to be linked with psychological well-being also in neurological populations [169]. Participation in community religious and cultural activities is associated with enhanced global cognition and better emotional states [93,100,102,103,104,105,108,110], these benefits extend to clinical contexts [170,171]. These findings are further substantiated by Pruitt et al. [172], which demonstrated that social engagement interventions led to enhancements in the DAN connectivity. The findings of this study provide biological evidence that social recreational activities exert measurable effects on brain function and stress regulation mechanisms.

Furthermore, emotional benefits have been shown to extend to shared musical participation in a healthy population, where singing in an amateur choir has been linked to increased positive affect and reduced negative affect [173]. From a neurophysiological perspective, Lenemann et al. [107] demonstrated a significant reduction in cortisol levels in participants who listened to music in a shared social context, indicating a decrease in physiological stress. This phenomenon has been extensively documented in the extant literature, with numerous studies and meta-analyses consistently demonstrating that music listening leads to a reduction in cortisol, HR, and blood pressure levels [174,175].

Moreover, social interactions, even when experienced online, result in enhanced semantic fluency and elevated levels of social satisfaction [92,94,96,97]. Furthermore, Dodge et al. [95] utilised fMRI to demonstrate heightened functional connectivity within the DAN in individuals engaged in structured online interpersonal dialogues. Comparable technology-based approaches (e.g., metaverse-based interventions, teleassistance interventions) show promising beneficial effects also in paediatric populations with neurological disorders [176,177].

Numerous studies have demonstrated that adult and elderly populations who participate in organised volunteer and social support groups have exhibited enhanced overall cognition [90,91,98,99,101,106,109]. These benefits extend to clinical populations, including individuals with neurological conditions [178,179]. Furthermore, a systematic review which examined social relationships and cognitive functioning in healthy adults aged 50 and older shows that the association depends on different types of social activities and specific cognitive domains [180]. Ybarra et al. [181] report in young adults aged 18–21, that 10 min of social interaction improved working-memory and processing-speed performance. As for the cognitive activities, although these studies did not resolve the reverse causation issue, globally the findings support the importance of socialization in boosting cognitive functioning and psychological well-being.

### 4.3. Physical Leisure Activities

In the physical domain, these activities are associated with benefits for cognitive functioning and emotional well-being, as confirmed by established literature. The most frequently investigated activities include walking, swimming, cycling, climbing stairs, domestic activities, aerobic and mobility exercises, running, volleyball, dancing, martial arts, table tennis, yoga, muscle strengthening, skiing, and golf.

Aerobic and non aerobic land-based activities such as walking, golf, endurance and muscle strength show a link to enhanced cognitive performance and emotional well-being in healthy subjects [113,114,115,116,117,118], with comparable benefits observed also in clinical populations [46,182]. A systematic review of healthy adults aged 18–45 found a small overall cognitive benefit of acute exercise (a single bout of exercise), with analyses of executive function tasks indicating improvements in working memory and inhibition [183]. Further evidence pertaining to cognitive and affective outcomes is provided by Hogan et al. [184]. Following a 15 min period of moderate stationary cycling, community-dwelling adults exhibited faster responses on a working-memory task and high-arousal positive affect in comparison to the control group. However, the negative affect remained unaltered [184]. Evidence is provided by Voelcker-Rehage et al. [185], who reported improvements in executive functioning and perceptual speed after 12-month long aerobic training (cardiovascular and coordination training) in cognitively healthy elderly. During an executive-control fMRI task, both interventions were associated with reduced prefrontal activation, whereas cardiovascular training was associated with increased activation in the sensorimotor network and coordination training with increased activation in the visuospatial network [185]. A study that collected blood samples from participants who practised dance, martial arts and treadmill walking showed an increase in peripheral concentration of BDNF levels [133]. These findings are consistent with a possible contribution of physical activity to neuroplasticity-related processes.

Moreover, several studies indicate that outdoor activities in natural environments are more psychologically beneficial than those performed in urban contexts [123,124,125,186]. Two studies, additionally, have demonstrated a significant reduction in HR in participants who engaged in both golf and walking (Nordic and conventional) while only walking activities showed more impact on cognitive functions [113,114]. Conversely, other studies have demonstrated an increase in HR in participants who engaged in strenuous physical activities, including skiing, mountain walking, muscle and endurance activities, and stair climbing. Individuals engaged in such physical activities experienced an enhancement in feelings of pleasure, motivation, and happiness [123,124,136,137]. The present findings appear to suggest that the intensity of physical exercise may influence the effect that activity has on psychological well-being, with implications for both the cardiovascular system and emotional sensations.

Aquatic activities exhibit the same pattern of effects across elderly and young populations alike [119,120,121,122], extending to the early phase of neurodegenerative disease [47,182,187,188]. A recent study shows that moderate-intensity in a single bout of aquatic treadmill exercise was associated with increased cerebral blood flow and cognitive functions [120]. Again several studies confirm that higher-intensity physical activities are associated with better executive functions (cognitive flexibility and motivation), performance perception, mood, and sleep quality [131,132,133,134,135,136,137,138,189,190].

Mind–body activities such as yoga show similar cognitive benefits [126,127,128], also in neurological and psychiatric populations [191,192,193,194,195,196,197]. Furthermore, an EEG study [126] in participants who engaged in yoga observed that relaxing activities may exert a positive effect on the Delta-band frequency, suggesting, therefore, a more restorative effect on brain functions during sleep.

Finally, domestic and everyday activities (such as commuting from home to work) are associated with improved attention, memory, and executive functions [139,140,141]. In support of this, Dupré et al. [198] demonstrated that executive functions and verbal fluency were better preserved also in older adults (>72 years old) who performed moderate to high levels of household/transportation activities.

Collectively, these findings are consistent with a possible contribution of physical activity to neuroplasticity-related processes. This contribution may depend on several factors, both related to the methodologies employed (type, duration, intensity, and frequency of exercise) and to individual variables (such as age, educational level, health status, fitness level, personality, social resources, socio-economic status, baseline cognitive status). However, it is worth noting that these neuroplasticity-related processes may accompany, rather than directly demonstrate, an effect on CR.

### 4.4. Conceptual and Methodological Considerations

The concept of CR is a theoretical construct that is not directly observable. It is inferred from the discrepancy between an individual’s functional performance and the one predicted based on their degree of brain aging or pathology [1,2,3]. The studies reviewed here assessed cognitive and emotional outcomes and, in a minority of cases, neurophysiological changes.

The present review offers new perspectives by bringing together evidence on traditional leisure activities (such as reading, writing, painting, individual sports, and going to the cinema) and experiences less commonly considered as recreational stimulating activities (for example, traveling and technology-mediated social activities) within established frameworks of leisure engagement and CR. In fact, a key conceptual issue is that the cognitive demand of an activity is not fixed, but rather varies according to individual characteristics. For instance, activities traditionally considered stimulating (such as the use of mobile phones, computers, and the internet) are now in common use and may therefore no longer be perceived as cognitively demanding, particularly for young adults. Nevertheless, these same activities could still exert a stimulating effect on older individuals. This is consistent with evidence indicating that it is how an activity is engaged (e.g., in terms of novelty and active participation), rather than the activity itself, that shapes cognitive benefits. In the Synapse Project, older adults who spent three months learning a cognitively demanding new skill (digital photography, quilting, or both) showed improved episodic memory. Conversely, the control group, who spent the same amount of time on equally frequent but less demanding activities (such as cooking or watching TV), showed no such improvement [67]. A scoping review synthesizing findings across a wide range of leisure activities reached the same conclusion: the novelty and active engagement involved in an activity, rather than the activity itself, determine its cognitive benefit [199]. This consideration highlights the need to periodically reassess which activities remain cognitively demanding, rather than treating cognitive demand as a fixed property of the activity. The present review deliberately included both activities traditionally assessed by established leisure measures and forms of leisure engagement that may be less readily captured by such predefined categories. In contrast to many previous reviews that focused on a single domain, this study considers multiple domains collectively, thus facilitating a more comprehensive and comparative analysis of the evidence across diverse areas of leisure engagement. This broader perspective underscores the potential limitations of prevailing measurement approaches, as socially shared and context-dependent experiences, such as traveling with others or attending concerts and live performances, may be inadequately represented by predefined activity categories. Conversely, activities such as sewing and knitting, which have historically been part of standard leisure scales, may no longer be as representative of the leisure patterns of younger generations. Together, these factors demonstrate that leisure measures need to be updated and adapted as recreational practices evolve throughout the adult life course.

### 4.5. Limits

It is imperative to acknowledge the limitations of this narrative review when interpreting the results.

Firstly, by its very nature, this narrative approach does not adopt a systematic protocol or quantitative meta-analytic approach; rather, it proposes a broader, panoramic overview of a heterogeneous literature which is not a shortcoming relative to systematic reviews but reflects a different, complementary purpose. This choice, however, means that the selection of studies was not conducted in accordance with rigorously standardised criteria, which may have introduced a degree of selection bias.

The inclusion criteria demanded that studies report cognitive and/or emotional outcomes associated with cognitive, social, or physical leisure activities.

Neurophysiological and neurobiological effects were considered as secondary outcomes. The activities examined included both practices commonly recognised as recreational activities and structured interventions subjected to experimental testing to assess their impact on well-being and potential contribution to cognitive reserve. The definition of a study as experimental was dependent upon the presence of controlled conditions or structured interventions on specific activities. Conversely, studies which analysed spontaneous behaviours or activities already present in the participants’ daily lives were classified as observational. This methodological approach facilitated a comprehensive perspective and ensured ecological validity; however, it inevitably introduced heterogeneity into the study designs and the methods of defining and measuring outcomes.

In addition, the included literature is characterised by significant methodological variability. A diversity of studies is evidenced by marked differences in study design, sample characteristics, assessment tools used, and operational definitions of recreational activities. The intensity, duration, and frequency of engagement in activities also vary, which makes direct comparisons between results more complex.

With regard to the neurophysiological and neuroimaging outcomes, although these offer promising insights into possible underlying mechanisms, many of the studies rely on different sample sizes and heterogeneous methodologies.

Multimodal longitudinal research that simultaneously integrates structural, functional, and neuroendocrine markers remains limited.

Finally, cultural and contextual differences in leisure practices may influence the nature and meaning of the activities studied, reducing the generalizability of the findings to different populations.

Unfortunately, only few studies directly assessed CR by using devoted questionnaires, while no study has considered education as an explicit proxy of CR. The lack of these measures limits the possibility of directly linking the various observed outcomes to the CR framework. We can nonetheless speculate that sustained engagement in diverse leisure activities, which play a key role in cognitive function and psychological well-being, might foster an individual’s CR capacity. Based on the reviewed results, we argue that larger longitudinal cohort studies are essential to observe whether leisure activity engagement across the lifespan prevents dementia risk or delays the onset of age-related cognitive decline, thereby establishing leisure activities as genuine reserve-building factors.

## 5. Conclusions

The studies reviewed here converge in showing a consistent association between engagement in cognitive, social, and physical leisure activities and better cognitive function and emotional well-being in healthy adults, with preliminary support from neurobiological and neurophysiological findings in a subset of studies. Rather than concluding that leisure activities build CR, the available evidence supports the more cautious interpretation that engagement in stimulating activities is associated with cognitive and emotional benefits and, in a subset of studies, with neurophysiological and neurobiological changes that may be relevant to processes underlying CR, resilience, and brain maintenance. The relative contribution of each domain and activity also appears to vary across adulthood, suggesting that leisure-based recommendations may need to be tailored to age, baseline cognitive functioning, and personal preference rather than applied uniformly.

From a practical point of view the evidence reviewed here, while not yet establishing causality, supports encouraging regular engagement in a diverse range of cognitively, socially, and physically stimulating leisure activities across adulthood. It is evident that the most of the studies reviewed did not directly test adherence or enjoyment and the perceived pleasure as moderators of cognitive/emotional benefit. We sustain that the reward properties of leisure activities may differ based on the individual preference and identifying the preferred activities may sustain their engagement over time increasing the beneficial effect on cognition and on the “holistic” well-being.

Future research on leisure activities and their impact on CR should include: (1) longitudinal and randomized controlled study designs to establish causal relationships; (2) multimodal study designs that jointly evaluate cognitive, emotional, and psychological outcomes; (3) neuroimaging techniques to investigate the structural and functional neural correlates underpinning these outcomes; (4) standardized instruments that directly quantify leisure activity engagement across the lifespan; (5) the assessment of non-traditional leisure activities; and (6) the investigation of reward and reinforcement processes linked to leisure engagement.

## Data Availability

No new data were created or analyzed in this study. Data sharing is not applicable to this study.

## Abbreviations

The following abbreviations are used in this manuscript:

AC-FL	Arithmetic Ability
ADAS-Cog	Alzheimer’s Disease Assessment Scale
ADL	Activities of Daily Living
AF	Animal Fluency Test
AFML	Adaptive Functions of Music Listening Scale
AI	Aerobic Intervention
AIM	Abstract Matching
AMNART	American National Adult Reading Test
ANB	Auditory N-Back Task
AR	Arithmetic
AST	Auditory Stroop Task
ATM	Aquatic Treadmill exercise
ATS	Auditory Task-Switching Paradigm
BART	Balloon Analog Risk Task
BBQ-36	36-item Bilingual Questionnaire (Vietnamese–English)
BD	Block Design
BDI	Beck Depression Inventory
BDNF	Brain-Derived Neurotrophic Factor
BM	Brain Maintenance
BMIS	Brief Mood Introspection Scale
BMRQ	Barcelona Music Reward Questionnaire
BNT	Boston Naming Test
BR	Brain Reserve
BSCS	Brief Self-Control Scale
BTACT	Brief Test of Adult Cognition by Telephone
CANTAB	Cambridge Neuropsychological Test Automated Battery
CASP-12	Control, Autonomy, Self-realization and Pleasure scale
CBF	Cerebral Blood Flow
CBT	Corsi Block-Tapping Task
CCT	Cube Copying Test
CD	Coding
CDMNS	Clinical Decision Making in Nursing Scale
CDR	Clinical Dementia Rating
CDT	Clock Drawing Test
CERAD	Consortium to Establish a Registry for Alzheimer’s Disease
CERAD-DR	Consortium to Establish a Registry for Alzheimer’s Disease Recall Module
CERAD-WL	Consortium to Establish a Registry for Alzheimer’s Disease Word Learning
CES-D	Center for Epidemiologic Studies Depression scale
CF	Category Fluency Task
CFA	Category Fluency Animals Test
CG	Control Group
CLAS	Cognitive Leisure Activity Scale
CMC	Choice Motor Correct Responses
CMCV	Choice Motor Coefficient of Variation
COG	Cognitive Training
CON	Video-Watching Control
COWAT	Controlled Oral Word Association Test
CR	Cognitive Reserve
CRASH	Cognitive Reserve Assessment Scale in Health
CRIq	Cognitive Reserve Index Questionnaire
CRQ	Cognitive Reserve Questionnaire
CRT	Visual 2-Choice Reaction Time Task
CSID	Community Screening Instrument for Dementia
CS-IR/DR	Craft Story Immediate and Delayed Recall
CTSB	Cathepsin B
CVLT-II	California Verbal Learning Test, Second edition
CWST	Color–Word Stroop Test
DAN	Dorsal Attention Network
DASS-21	Depression, Anxiety, and Stress Scales
DCT	Digit-Comparison Tasks
DOT	Digit Ordering Test (WAIS-III adapted)
DPAS	Dijon Physical Activity Score
DS	Digit Span
DTWT	Dual-Task Walking Test
DWR	Delayed Word Recall
ECG	Electrocardiogram
EEG	Electroencephalogram
EF	Executive Function
EI	Emotional Intelligence
EMA	Ecological Momentary Assessment
EP	Electrophysiology
ERQ	Emotion Regulation Questionnaire
ERR	Emotion Expression Recognition
ERT	Emotion Recognition Task
ET	Eye-tracking
EURO-D	European Depression Scale
EX	Aerobic Exercise
EXCOG	Combined Aerobic Exercise and Cognitive Training
EXE	Exercise
EXP	Experts
F	Female
FAB	Frontal Assessment Battery
FAQ-CZ	The Questionnaire of Functional State
FAS	Felt Arousal Scale
FAS verbal fluency	F-A-S verbal fluency
FCAS	Florida Cognitive Activities Scale
FDT	The Five-Digit Test
fMRI	functional Magnetic Resonance Imaging
FPRTs	First-pass Reading Times
FR	Face Recognition
FS	Feeling Scale
FT	Flanker Tasks
FTT	Finger Tapping Test
GDS	Geriatric Depression Scale
GE-I	Global Enrichment Index
GPAQ	Global Physical Activity Questionnaire
GSLTPAQ	Godin-Shephard Leisure-Time Physical Activity Questionnaire
HALAs	Handicraft art leisure activities i
HAM	High Activity Musicians
HDS-R	Hasegawa Dementia Score-Revised
HH	Heavy Housework
HPA	Hypothalamic–Pituitary–Adrenal
HR	Heart Rate
HRR	Heart Rate Recovery
HSRT	High-Speed Resistance Training
HT	Hotel Task
HVLT	Hopkins Verbal Learning Test
IADL	Instrumental Activities of Daily Living
ICSS	Inclusion of Community in the Self Scale
IES	Impact of Event Scale
IG	Intervention Group
INF	Information
ISTAT	National Institute of Statistics
IPAQ	International Physical Activity Questionnaire
IU	Indiana University
IWR	Immediate Word Recall
JLO	Judgment of Line Orientation
LA	Leisure Activities
LAM	Low Activity Musicians
LAPAQ	Longitudinal Ageing Study Amsterdam Physical Activity Questionnaire
LEQ	Lifetime of Experiences Questionnaire
LF	Letter Fluency Task
LH	Light Housework
lIPL	left Inferior Parietal Lobule
LM	Logical Memory
LM-D	Logical Memory Delayed Recall
LM-I	Logical Memory Immediate Recall
LNS	Letter-Number Sequencing
LOT	Line Orientation Task
LSI	Leisure Score Index
lSPL	left Superior Parietal Lobule
LSBQ	Language and Social Background Questionnaire
LSNS	Lubben Social Network Scale
LTM	Land-based Treadmill exercise
LTQ	Leisure Travel Questionnaire
M	Mean
MAAS	The Mindful Attention Awareness Scale
MBEA	Montreal Battery of Evaluation of Amusia
mCRS	modified version of Cognitive Reserve Scale
MHC-SF	Mental Health Continuum—Short Form
MMSE	Mini-Mental State Examination
MoCA	Montreal Cognitive Assessment
MPT	Motor Praxis Task
MPQ	Music Preference Questionnaire
MRI	Magnetic Resonance Imaging
MRT	Matrix Reasoning Task
MSQ	Mood States Questionnaire
MSS	Mood Survey Scale
NACC UDS V3	National Alzheimer’s Coordinating Center—Uniform Data Set, Version 3
NART-15	National Adult Reading Test (15-item version)
NBACK	Fractal-2-back
NC	Neural Compensation
NIH	National Institutes of Health
NIH-EB	NIH Toolbox Emotion Battery
NM	Non Musicians
NOSQ	Neutral Objects Satisfaction Questionnaire
NOV	Novices
NPI	NeuropsychiatricInventory
NPP	Naming the President and Vice President
NR	Neural Reserve
OHQ	Oxford happiness questionnaire
ONT	Objective Naming Test
OPQOL	Older People’s Quality of Life-brief
OSPAN	Arithmetic Operation–word Memory Span task
PAL	Paragraphs About Leisure
PANAS	Positive and Negative Affect Schedule
PASE	Physical Activity Scale for the Elderly
PC	Proverb Comprehension
POMS	Profile of Mood States
PPP	Picture–Picture Priming
PSI-A	Problem Solving Inventory Form-A
PSS	Perceived Stress Scale
PSW-B	Psychological and Social Well-Being
PVT	Psychomotor Vigilance Task
PWB	Psychological Well-Being Questionnaire
QoL	Quality of Life
QPAR	Quick Physical Activity Rating
RAPA	Rapid Assessment of Physical Activity
RAVLT	Rey Auditory Verbal Learning Test
RBANS	Repeatable Battery for the Assessment of Neuropsychological Status
RBMT	Rivermead Behavioral Memory Test
RCD	Recall of the Current Date
RCFCT	Rey Complex Figure Copy Task
RCT	Randomized Controlled Trial
rIFG	Right Inferior Frontal Gyrus
RNG	Random Number Generation
ROCF	Rey–Osterrieth Complex Figure Test
RPE	Rating of Perceived Exertion
RPM	Raven’s Progressive Matrices
rSFG	right Superior Frontal Gyrus
s-CRIq	short Cognitive Reserve Index Questionnaire version
S7	Serial 7’s Test
SAM	Self-Assessment Manikin
SANRA	Scale for the Quality Assessment of Narrative Review Articles
SC	Sentence Comprehension
SDMT	Symbol Digit Modalities Test
SFHS	Short-Form Health Survey
SIM	Similarities
SL	Satisfaction in Life
SMT	Spatial Memory Task
SOC	Stockings of Cambridge Task
SPM	Standard Progressive Matrices
SRT	Auditory Simple Reaction Time Test
SSp	Spatial Span
SSB	Spatial Span Backwards
SSF	Spatial Span Forward
SSRS	Social Support Rating Scale
STAI	State–Trait Anxiety Inventory
STAI- Y	State–Trait Anxiety Inventory Y
STAI-S	State–Trait Anxiety Inventory State subscale
STW	Spot the Word
SVAMA	Italian Checklist for Multidimensional Assessment
SWLS	Satisfaction with Life Scale
TAP	Test of Attentional Performance
TICS	Telephone Interview for Cognitive Status
TIL	Three-Item Loneliness Scale
TMT	Trail Making Test
ToL	Tower of London
TRTs	Total Reading Times
UCL	A 12 item Museum Wellbeing Toolkit
UCLA	University of California, Los Angeles
V	Volleyball
VAMS	100-point Visual Analogue Mood Scales
VF	Verbal Fluency
VLS-ALQ	Victoria Longitudinal Study-Activity Lifestyle Questionnaire
VMST	Visual Memory Span Test
VNB	Visual N-Back Task
VOC	Vocabulary
VOLT	Visual Object Learning Task
VP	Visual Puzzles
VRM	Verbal Recognition Memory
VST	Visual Stroop Task
VSTM	Visual Short-Term Memory
VTS	Visual Task-Switching Paradigm
WAIS	Wechsler Adult Intelligence Scale
WCST	The Wisconsin Card Sorting Test
WEMWBS	Warwick–Edinburgh Mental Well-being Scale
WHII	Whitehall II Study
WHOQOL-BREF	World Health Organization Quality of Life Brief Questionnaire
WLM	Word List Memory
WM	Working Memory
WMS-III	Wechsler Memory Scale Third Edition
WMS-R	Wechsler Memory Scale—Revised
YI	Yoga Intervention

## References

[1] Stern Y., Arenaza-Urquijo E.M., Bartrés-Faz D., Belleville S., Cantilon M., Chetelat G., Ewers M., Franzmeier N., Kempermann G., Kremen W.S. (2020). Whitepaper: Defining and Investigating Cognitive Reserve, Brain Reserve, and Brain Maintenance. Alzheimer’s Dement..

[2] Stern Y. (2002). What Is Cognitive Reserve? Theory and Research Application of the Reserve Concept. J. Int. Neuropsychol. Soc..

[3] Stern Y. (2009). Cognitive Reserve. Neuropsychologia.

[4] Stern Y., Barnes C.A., Grady C., Jones R.N., Raz N. (2019). Brain Reserve, Cognitive Reserve, Compensation, and Maintenance: Operationalization, Validity, and Mechanisms of Cognitive Resilience. Neurobiol. Aging.

[5] Serra L., Gelfo F., Petrosini L., Di Domenico C., Bozzali M., Caltagirone C. (2018). Rethinking the Reserve with a Translational Approach: Novel Ideas on the Construct and the Interventions. J. Alzheimer’s Dis..

[6] Serra L., Gelfo F. (2019). What Good Is the Reserve? A Translational Perspective for the Managing of Cognitive Decline. Neural Regen. Res..

[7] Pappalettera C., Carrarini C., Miraglia F., Vecchio F., Rossini P.M. (2024). Cognitive Resilience/Reserve: Myth or Reality? A Review of Definitions and Measurement Methods. Alzheimer’s Dement..

[8] Ji L., Pearlson G.D., Hawkins K.A., Steffens D.C., Guo H., Wang L. (2018). A New Measure for Neural Compensation Is Positively Correlated with Working Memory and Gait Speed. Front. Aging Neurosci..

[9] Kivimäki M., Batty G.D., Singh-Manoux A. (2015). Pointing the FINGER at Multimodal Dementia Prevention. Lancet.

[10] Guzmán-Vélez E., Tranel D. (2015). Does Bilingualism Contribute to Cognitive Reserve? Cognitive and Neural Perspectives. Neuropsychology.

[11] Matyas N., Keser Aschenberger F., Wagner G., Teufer B., Auer S., Gisinger C., Kil M., Klerings I., Gartlehner G. (2019). Continuing Education for the Prevention of Mild Cognitive Impairment and Alzheimer’s-Type Dementia: A Systematic Review and Overview of Systematic Reviews. BMJ Open.

[12] Shatenstein B., Barberger-Gateau P., Mecocci P. (2015). Prevention of Age-Related Cognitive Decline: Which Strategies, When, and for Whom?. J. Alzheimer’s Dis..

[13] Nucci M., Mapelli D., Mondini S. (2012). Cognitive Reserve Index Questionnaire (CRIq): A New Instrument for Measuring Cognitive Reserve. Aging Clin. Exp. Res..

[14] Mondini S., Pucci V., Pastore M., Gaggi O., Tricomi P.P., Nucci M. (2023). S-CRIq: The Online Short Version of the Cognitive Reserve Index Questionnaire. Aging Clin. Exp. Res..

[15] Serra L., Musicco M., Cercignani M., Torso M., Spanò B., Mastropasqua C., Giulietti G., Marra C., Bruno G., Koch G. (2015). Cognitive Reserve and the Risk for Alzheimer’s Disease: A Longitudinal Study. Neurobiol. Aging.

[16] Rami L., Valls-Pedret C., Bartrés-Faz D., Caprile C., Solé-Padullés C., Castellvi M., Olives J., Bosch B., Molinuevo J.L. (2011). Cognitive reserve questionnaire. Scores obtained in a healthy elderly population and in one with Alzheimer’s disease. Rev. Neurol..

[17] Valenzuela M.J., Sachdev P. (2007). Assessment of Complex Mental Activity across the Lifespan: Development of the Lifetime of Experiences Questionnaire (LEQ). Psychol. Med..

[18] Relander K., Mäki K., Soinne L., García-García J., Hietanen M. (2021). Active Lifestyle as a Reflection of Cognitive Reserve: The Modified Cognitive Reserve Scale. Nord. Psychol..

[19] Amoretti S., Cabrera B., Torrent C., Bonnín C.D.M., Mezquida G., Garriga M., Jiménez E., Martínez-Arán A., Solé B., Reinares M. (2019). Cognitive Reserve Assessment Scale in Health (CRASH): Its Validity and Reliability. J. Clin. Med..

[20] Kuykendall L., Tay L., Ng V. (2015). Leisure Engagement and Subjective Well-Being: A Meta-Analysis. Psychol. Bull..

[21] Shin K., You S. (2013). Leisure Type, Leisure Satisfaction and Adolescents’ Psychological Wellbeing. J. Pac. Rim Psychol..

[22] Holder M.D., Coleman B., Sehn Z.L. (2009). The Contribution of Active and Passive Leisure to Children’s Well-Being. J. Health Psychol..

[23] Fancourt D., Aughterson H., Finn S., Walker E., Steptoe A. (2021). How Leisure Activities Affect Health: A Narrative Review and Multi-Level Theoretical Framework of Mechanisms of Action. Lancet Psychiatry.

[24] Đuranović M. (2024). Leisure Activities of Young People as a Predictor of Life Satisfaction. Int. J. Instr..

[25] Jopp D.S., Hertzog C. (2010). Assessing Adult Leisure Activities: An Extension of a Self-Report Activity Questionnaire. Psychol. Assess..

[26] Kleiber D., Larson R., Csikszentmihalyi M. (2014). The Experience of Leisure in Adolescence. Applications of Flow in Human Development and Education.

[27] Everard K.M., Lach H.W., Fisher E.B., Baum M.C. (2000). Relationship of Activity and Social Support to the Functional Health of Older Adults. J. Gerontol. Ser. B Psychol. Sci. Soc. Sci..

[28] Passmore A. (2003). The Occupation of Leisure: Three Typologies and Their Influence on Mental Health in Adolescence. OTJR Occup. Ther. J. Res..

[29] Ellis G.D., Rademacher C. (1987). Development of a Typology of Common Adolescent Free Time Activities: A Validation and Extension of Kleiber, Larson, and Csikszentmihalyi. J. Leis. Res..

[30] Stephan Y., Boiché J., Canada B., Terracciano A. (2014). Association of Personality with Physical, Social, and Mental Activities across the Lifespan: Findings from US and French Samples. Br. J. Psychol..

[31] Karp A., Paillard-Borg S., Wang H.-X., Silverstein M., Winblad B., Fratiglioni L. (2006). Mental, Physical and Social Components in Leisure Activities Equally Contribute to Decrease Dementia Risk. Dement. Geriatr. Cogn. Disord..

[32] Guerrero Barragán A., Lucumí D., Lawlor B. (2021). Association of Leisure Activities with Cognitive Impairment and Dementia in Older Adults in Colombia: A SABE-Based Study. Front. Neurol..

[33] Bartrés-Faz D., Arenaza-Urquijo E., Ewers M., Belleville S., Chételat G., Franzmeier N., Gonneaud J., Echevarri J.M.G., Okonkwo O., Schultz S. (2020). Theoretical Frameworks and Approaches Used within the Reserve, Resilience and Protective Factors Professional Interest Area of the Alzheimer’s Association International Society to Advance Alzheimer’s Research and Treatment. Alzheimer’s Dement. Diagn. Assess. Dis. Monit..

[34] Hultsch D.F., Hertzog C., Small B.J., Dixon R.A. (1999). Use It or Lose It: Engaged Lifestyle as a Buffer of Cognitive Decline in Aging?. Psychol. Aging.

[35] Tinsley H.E.A., Eldredge B.D. (1995). Psychological Benefits of Leisure Participation: A Taxonomy of Leisure Activities Based on Their Need-Gratifying Properties. J. Couns. Psychol..

[36] Galvin J.E., Tolea M.I., Chrisphonte S. (2021). The Cognitive & Leisure Activity Scale (CLAS): A New Measure to Quantify Cognitive Activities in Older Adults with and without Cognitive Impairment. Alzheimer’s Dement..

[37] Schinka J.A., Mcbride A., Vanderploeg R.D., Tennyson K., Borenstein A.R., Mortimer J.A. (2005). Florida Cognitive Activities Scale: Initial Development and Validation. J. Int. Neuropsychol. Soc..

[38] Anatürk M., Suri S., Zsoldos E., Filippini N., Mahmood A., Singh-Manoux A., Kivimäki M., Mackay C.E., Ebmeier K.P., Sexton C.E. (2020). Associations Between Longitudinal Trajectories of Cognitive and Social Activities and Brain Health in Old Age. JAMA Netw. Open.

[39] Godin G. (2011). The Godin-Shephard Leisure-Time Physical Activity Questionnaire. Health Fit. J. Can..

[40] Amireault S., Godin G. (2015). The Godin-Shephard Leisure-Time Physical Activity Questionnaire: Validity Evidence Supporting Its Use for Classifying Healthy Adults into Active and Insufficiently Active Categories. Percept. Mot. Ski..

[41] Galvin J.E., Tolea M.I., Rosenfeld A., Chrisphonte S. (2020). The Quick Physical Activity Rating (QPAR) Scale: A Brief Assessment of Physical Activity in Older Adults with and without Cognitive Impairment. PLoS ONE.

[42] Fitzgerald M., Joseph A.P., Hayes M., O’Regan M. (1995). Leisure Activities of Adolescent Schoolchildren. J. Adolesc..

[43] Yates L.A., Ziser S., Spector A., Orrell M. (2016). Cognitive Leisure Activities and Future Risk of Cognitive Impairment and Dementia: Systematic Review and Meta-Analysis. Int. Psychogeriatr..

[44] Song S., Stern Y., Gu Y. (2022). Modifiable Lifestyle Factors and Cognitive Reserve: A Systematic Review of Current Evidence. Ageing Res. Rev..

[45] Su S., Shi L., Zheng Y., Sun Y., Huang X., Zhang A., Que J., Sun X., Shi J., Bao Y. (2022). Leisure Activities and the Risk of Dementia: A Systematic Review and Meta-Analysis. Neurology.

[46] Yang X., Xu X.Y., Guo L., Zhang Y., Wang S.S., Li Y. (2022). Effect of Leisure Activities on Cognitive Aging in Older Adults: A Systematic Review and Meta-Analysis. Front. Psychol..

[47] Serra L., Petrosini L., Mandolesi L., Bonarota S., Balsamo F., Bozzali M., Caltagirone C., Gelfo F. (2022). Walking, Running, Swimming: An Analysis of the Effects of Land and Water Aerobic Exercises on Cognitive Functions and Neural Substrates. Int. J. Environ. Res. Public Health.

[48] Duffner L., De Jong N., Backes W.H., Jansen J.F., De Vugt M., Deckers K., Koehler S. (2021). The Relationship between Cognitive and Social Leisure Activities across the Lifespan and Neural Markers for Brain Health: A Systematic Literature Review. Alzheimer’s Dement..

[49] Sukhera J. (2022). Narrative Reviews: Flexible, Rigorous, and Practical. J. Grad. Med. Educ..

[50] Grant M.J., Booth A. (2009). A Typology of Reviews: An Analysis of 14 Review Types and Associated Methodologies. Health Inf. Libr. J..

[51] Baethge C., Goldbeck-Wood S., Mertens S. (2019). SANRA—A Scale for the Quality Assessment of Narrative Review Articles. Res. Integr. Peer Rev..

[52] Karsazi H., Rezapour T., Ghamsari A.S.M., Kormi-Nouri R., Hatami J. (2024). Which Intellectual Activities Are Related to Cognitive Reserve? Introduction and Testing a Three-Dimensional Model. Psychol. Res..

[53] Wang Y., Wang S., Zhu W., Liang N., Zhang C., Pei Y., Wang Q., Li S., Shi J. (2022). Reading Activities Compensate for Low Education-Related Cognitive Deficits. Alzheimer’s Res. Ther..

[54] Mao C., Li Z.-H., Lv Y.-B., Gao X., Kraus V.B., Zhou J.-H., Wu X.-B., Shi W.-Y., Li F.-R., Liu S.-M. (2020). Specific Leisure Activities and Cognitive Functions Among the Oldest-Old: The Chinese Longitudinal Healthy Longevity Survey. J. Gerontol. Ser. A.

[55] Klein K., Boals A. (2001). Expressive Writing Can Increase Working Memory Capacity. J. Exp. Psychol. Gen..

[56] Cheung R.Y.M., Chung H.S., Ng M.C.Y. (2021). Expressive Writing and Well-Being in Chinese Emerging Adults: Is Emotion Regulation an Underlying Mechanism?. Emerg. Adulthood.

[57] Urwyler P., Gupta R.K., Falkner M., Niklaus J., Müri R.M., Nef T. (2023). Tablet-Based Puzzle Game Intervention for Cognitive Function and Well-Being in Healthy Adults: Pilot Feasibility Randomized Controlled Trial. JMIR Aging.

[58] Martinez L., Gimenes M., Lambert E. (2023). Video Games and Board Games: Effects of Playing Practice on Cognition. PLoS ONE.

[59] Kaynak S., Ergün S., Karadaş A. (2023). The Effect of Crossword Puzzle Activity Used in Distance Education on Nursing Students’ Problem-Solving and Clinical Decision-Making Skills: A Comparative Study. Nurse Educ. Pract..

[60] Bubbico G., Chiacchiaretta P., Parenti M., Di Marco M., Panara V., Sepede G., Ferretti A., Perrucci M.G. (2019). Effects of Second Language Learning on the Plastic Aging Brain: Functional Connectivity, Cognitive Decline, and Reorganization. Front. Neurosci..

[61] Chen J., Li Z., Xiong Z., Liu G. (2025). Working Memory as the Focus of the Bilingual Effect in Executive Functions. Behav. Sci..

[62] Gabriel D., Devos J., Chapuis S., Comte A., Billot P.-E. (2025). Watching Live Performances Enhances Subjective and Physiological Emotional Responses Compared to Viewing the Same Performance on Screen. IBRO Neurosci. Rep..

[63] Drake J.E., Hastedt I., James C. (2016). Drawing to Distract: Examining the Psychological Benefits of Drawing over Time. Psychol. Aesthet. Creat. Arts.

[64] Bone J.K., Fancourt D., Sonke J.K., Bu F. (2024). Participatory and Receptive Arts Engagement in Older Adults: Associations with Cognition over a Seven-Year Period. Creat. Res. J..

[65] Jiang X., Lu X., Pu M., Li X. (2025). The Impact of Craft Creation Practice on University Students’ Mental Health: A Moderated Network Analysis. Front. Public Health.

[66] Mashinchi G.M., McFarland C.P., Hall S., Strongin D.L., Williams G.A., Cotter K.A. (2024). Handicraft Art Leisure Activities and Cognitive Reserve. Clin. Neuropsychol..

[67] Park D.C., Lodi-Smith J., Drew L., Haber S., Hebrank A., Bischof G.N., Aamodt W. (2014). The Impact of Sustained Engagement on Cognitive Function in Older Adults: The Synapse Project. Psychol. Sci..

[68] Iizuka A., Ogawa S., Cho D., Fujihira K., Li Y., Sato K., Hinakura K., Kobayashi-Cuya K.E., Suzuki H. (2025). The Effects of the MONOZUKURI Program on Executive Function Among Community-Dwelling Older Adults: A Randomized Controlled Trial. Brain Behav..

[69] Wang M.-Y., Chang C.-Y., Su S.-Y. (2011). What’s Cooking?—Cognitive Training of Executive Function in the Elderly. Front. Psychol..

[70] Linnemann A., Ditzen B., Strahler J., Doerr J.M., Nater U.M. (2015). Music Listening as a Means of Stress Reduction in Daily Life. Psychoneuroendocrinology.

[71] Van Goethem A., Sloboda J. (2011). The Functions of Music for Affect Regulation. Music. Sci..

[72] Ferreri L., Rodriguez-Fornells A. (2017). Music-Related Reward Responses Predict Episodic Memory Performance. Exp. Brain Res..

[73] Vincenzi M., Borella E., Sella E., Lima C.F., De Beni R., Schellenberg E.G. (2022). Music Listening, Emotion, and Cognition in Older Adults. Brain Sci..

[74] MacRitchie J., Breaden M., Milne A.J., McIntyre S. (2020). Cognitive, Motor and Social Factors of Music Instrument Training Programs for Older Adults’ Improved Wellbeing. Front. Psychol..

[75] Hanna-Pladdy B., MacKay A. (2011). The Relation between Instrumental Musical Activity and Cognitive Aging. Neuropsychology.

[76] Seinfeld S., Figueroa H., Ortiz-Gil J., Sanchez-Vives M.V. (2013). Effects of Music Learning and Piano Practice on Cognitive Function, Mood and Quality of Life in Older Adults. Front. Psychol..

[77] Wang X., Yamashita M., Guo X., Stiernman L., Kakihara M., Abe N., Sekiyama K. (2025). Never Too Late to Start Musical Instrument Training: Effects on Working Memory and Subcortical Preservation in Healthy Older Adults across 4 Years. Imaging Neurosci..

[78] Anderson J.A.E., Mak L., Keyvani Chahi A., Bialystok E. (2018). The Language and Social Background Questionnaire: Assessing Degree of Bilingualism in a Diverse Population. Behav. Res. Methods.

[79] Nater U.M., Krebs M., Ehlert U. (2005). Sensation Seeking, Music Preference, and Psychophysiological Reactivity to Music. Music. Sci..

[80] Mas-Herrero E., Marco-Pallares J., Lorenzo-Seva U., Zatorre R.J., Rodriguez-Fornells A. (2013). Individual Differences in Music Reward Experiences. Music Percept..

[81] Groarke J.M., Hogan M.J. (2018). Development and Psychometric Evaluation of the Adaptive Functions of Music Listening Scale. Front. Psychol..

[82] Weziak-Bialowolska D., Bialowolski P., Sacco P.L. (2023). Mind-Stimulating Leisure Activities: Prospective Associations with Health, Wellbeing, and Longevity. Front. Public Health.

[83] Plummer J., Nguyen K., Everly J., Kiesow A., Leith K., Neils-Strunjas J. (2023). How an Intergenerational Book Club Can Prevent Cognitive Decline in Older Adults: A Pilot Study. Gerontol. Geriatr. Med..

[84] Baroi B. (2024). Impact of Travel and Recreational Activities on Stress, Happiness, and Life Satisfaction among University Students. Sci. Eng. Health Stud..

[85] Rehman S., Ahmad J., Khan A., Alotaibi K.A. (2025). Frequency of Leisure Travel and Psychological Well-Being in Pharmacists: The Sequential Mediating Roles of Perceived Stress and Social Support. BMC Psychol..

[86] Pugh E., De Vito A., Divers R., Robinson A., Weitzner D.S., Calamia M. (2021). Social Factors That Predict Cognitive Decline in Older African American Adults. Int. J. Geriatr. Psychiatry.

[87] Segel-Karpas D., Lachman M.E. (2018). Social Contact and Cognitive Functioning: The Role of Personality. J. Gerontol. Ser. B Psychol. Sci. Soc. Sci..

[88] Mai S., Cai J., Zheng W., Li L. (2023). Path Analysis of Social Engagement, Loneliness and Cognitive Function among Chinese Older Adults. Psychol. Health Med..

[89] Mao S., Xie L., Lu N. (2023). Activity Engagement and Cognitive Function among Chinese Older Adults: Moderating Roles of Gender and Age. BMC Geriatr..

[90] Yu C., Barr A.B. (2025). Social Activities and Cognitive Functioning Across Mid- and Late Life: Evidence from China. Gerontol..

[91] Jang H., Zhang S., Han S.H. (2026). Social Activities with Friends and Neighbors in Relation to Cognitive Function in Later Life: The Benefits of Contact and Helping. Res. Aging.

[92] Zhaoyang R., Scott S.B., Martire L.M., Sliwinski M.J. (2021). Daily Social Interactions Related to Daily Performance on Mobile Cognitive Tests among Older Adults. PLoS ONE.

[93] Borgeest G.S., Henson R.N., Shafto M., Samu D., Cam-CAN, Kievit R.A. (2020). Greater Lifestyle Engagement Is Associated with Better Age-Adjusted Cognitive Abilities. PLoS ONE.

[94] Jeon S., Charles S.T. (2024). Internet-Based Social Activities and Cognitive Functioning 2 Years Later Among Middle-Aged and Older Adults: Prospective Cohort Study. JMIR Aging.

[95] Dodge H.H., Yu K., Wu C.-Y., Pruitt P.J., Asgari M., Kaye J.A., Hampstead B.M., Struble L., Potempa K., Lichtenberg P. (2024). Internet-Based Conversational Engagement Randomized Controlled Clinical Trial (I-CONECT) Among Socially Isolated Adults 75+ Years Old with Normal Cognition or Mild Cognitive Impairment: Topline Results. Gerontol..

[96] Mois G., Lydon E.A., Danilovich M.K., Myers D., Chen X.S., Mudar R.A., Rogers W.A. (2025). A Social Engagement Technology-Based Randomized Controlled Trial for Older Adults: Outcomes and Lessons Learned. Contemp. Clin. Trials Commun..

[97] Nakahara K., Yokoi K. (2024). Role of Meaningful Social Participation and Technology Use in Mitigating Loneliness and Cognitive Decline Among Older Adults. Am. J. Occup. Ther..

[98] Cohn-Schwartz E. (2020). Pathways from Social Activities to Cognitive Functioning: The Role of Physical Activity and Mental Health. Innov. Aging.

[99] Hyun J., Cerino E.S., Katz M.J., Lovasi G., Lipton R.B., Sliwinski M.J. (2025). Neighborhood Bonding and Bridging Social Capital, Social Activity Participation, and Short-Term Cognitive Variability in Later Life. J. Gerontol. Ser. B Psychol. Sci. Soc. Sci..

[100] Chen A., Ang S. (2026). Social Participation and Cognitive Function in Chinese Older Couples: Urban-Rural Variations in Partner Effects. Soc. Sci. Med..

[101] Ma T., Liao J., Ye Y., Li J. (2024). Social Support and Cognitive Activity and Their Associations with Incident Cognitive Impairment in Cognitively Normal Older Adults. BMC Geriatr..

[102] Son J., Sung P. (2023). The Reciprocal Relationship Between Social Engagement and Cognitive Function Among Older Adults in South Korea. J. Appl. Gerontol..

[103] Fancourt D., Steptoe A. (2018). Cultural Engagement Predicts Changes in Cognitive Function in Older Adults over a 10 Year Period: Findings from the English Longitudinal Study of Ageing. Sci. Rep..

[104] Rathje S., Hackel L., Zaki J. (2021). Attending Live Theatre Improves Empathy, Changes Attitudes, and Leads to pro-Social Behavior. J. Exp. Soc. Psychol..

[105] Kühnapfel C., Trupp M., Pelowski M., Fingerhut J. (2025). On the Impact of Public Art: How Engaging a Pedestrian-Level Exhibition Improves Neighborhood Connectedness and Well-Being. Wellbeing Space Soc..

[106] Pitkala K.H., Routasalo P., Kautiainen H., Sintonen H., Tilvis R.S. (2011). Effects of Socially Stimulating Group Intervention on Lonely, Older People’s Cognition: A Randomized, Controlled Trial. Am. J. Geriatr. Psychiatry.

[107] Linnemann A., Strahler J., Nater U.M. (2016). The Stress-Reducing Effect of Music Listening Varies Depending on the Social Context. Psychoneuroendocrinology.

[108] Wang H.-X., Jin Y., Hendrie H.C., Liang C., Yang L., Cheng Y., Unverzagt F.W., Ma F., Hall K.S., Murrell J.R. (2013). Late Life Leisure Activities and Risk of Cognitive Decline. J. Gerontol. A. Biol. Sci. Med. Sci..

[109] Krueger K.R., Wilson R.S., Kamenetsky J.M., Barnes L.L., Bienias J.L., Bennett D.A. (2009). Social Engagement and Cognitive Function in Old Age. Exp. Aging Res..

[110] Dixon J.S., Amariglio R.E., Howard M., Cella K., Farias S.T., Jackson M.J., Krueger K.R., Sachs B.C., Snyder H., Tangney C.C. (2025). The Association between Social Engagement and Cognition in Older Adults: Examining Sex and Ethnoracial Group as Moderators. J. Gerontol. Ser. B Psychol. Sci. Soc. Sci..

[111] Lubben J., Blozik E., Gillmann G., Iliffe S., Von Renteln Kruse W., Beck J.C., Stuck A.E. (2006). Performance of an Abbreviated Version of the Lubben Social Network Scale Among Three European Community-Dwelling Older Adult Populations. Gerontol..

[112] Lubben J.E. (1988). Assessing Social Networks among Elderly Populations. Fam. Community Health.

[113] Kettinen J., Tikkanen H., Hiltunen M., Murray A., Horn N., Taylor W.R., Venojärvi M. (2023). Cognitive and Biomarker Responses in Healthy Older Adults to a 18-Hole Golf Round and Different Walking Types: A Randomised Cross-over Study. BMJ Open Sport Exerc. Med..

[114] Torre M.M., Carrubba C., Langeard A., Hugues N., Laurin J., Temprado J.-J. (2024). Is an 8-Week Regimen of Nordic Walking Training Sufficient to Benefit Cognitive Performance in Healthy Older Adults? A Pilot Study. J. Clin. Med..

[115] Loprinzi P.D., Kane C.J. (2015). Exercise and Cognitive Function. Mayo Clin. Proc..

[116] Singh H. (2017). Relationship Between Leisure-Time Physical Activity And Emotional Intelligence in Female University Students: A Correlational Study. Eur. J. Phys. Educ. Sport Sci..

[117] Potoczny W., Herzog-Krzywoszańska R., Krzywoszański Ł. (2025). Regular Physical Activity and Life Satisfaction: Unpacking the Roles of Self-Control and Emotion Regulation. Appl. Sci..

[118] Yang J., Zheng X., Wang Y., Wang H., Song G. (2024). The Relationship between Leisure Time Physical Activity and Cognitive Function in Older Americans. J. Frailty Aging.

[119] Canton-Martinez E., Rentería I., Machado Parra J.P., Avilés-Reyes R., Hall-López J.A., Ochoa-Martínez P.Y., García Suárez P.C., Moncada-Jiménez J., Johnson D.K., Jiménez Maldonado A. (2025). Effect of a Short-Term Water-Based Exercise Program on Cognitive Skills in a Mexican Older Adult Population. Front. Hum. Neurosci..

[120] So B., Cheung C.L., Zheng C.Y., Kwok Y.P., Man M.Y., Mok Y.K., Tang F., Ng G., Sze C.N., Tang N.L. (2024). Effect of Moderate-Intensity Aquatic Treadmill Exercise on Cognitive Function and Cerebral Blood Flow for Healthy Older Adults. Exp. Gerontol..

[121] Terra De Oliveira R., Lino T.B., Scarmagnan G.S., Miziara Barbosa S.R., De Souza Pegorare A.B.G., Christofoletti G. (2024). A Controlled Clinical Trial on the Effects of Aquatic Exercise on Cognitive Functions in Community-Dwelling Older Adults. Brain Sci..

[122] Abou-Dest A., Albinet C.T., Boucard G., Audiffren M. (2012). Swimming as a Positive Moderator of Cognitive Aging: A Cross-Sectional Study with a Multitask Approach. J. Aging Res..

[123] Niedermeier M., Einwanger J., Hartl A., Kopp M. (2017). Affective Responses in Mountain Hiking—A Randomized Crossover Trial Focusing on Differences between Indoor and Outdoor Activity. PLoS ONE.

[124] Krautgasser S., Scheiber P., Duvillard S.P.V., Müller E. (2012). Heart Rate, Mood States, and Rating of Perceived Exertion Among Elderly Subjects During 3.5 Hours of Recreational Alpine Skiing. Ann. Kinesiol..

[125] De Brito J.N., Pope Z.C., Mitchell N.R., Schneider I.E., Larson J.M., Horton T.H., Pereira M.A. (2019). Changes in Psychological and Cognitive Outcomes after Green versus Suburban Walking: A Pilot Crossover Study. Int. J. Environ. Res. Public. Health.

[126] Datta K., Bhutambare A., V.L. M., Narawa Y., Srinath R., Kanitkar M. (2023). Improved Sleep, Cognitive Processing and Enhanced Learning and Memory Task Accuracy with Yoga Nidra Practice in Novices. PLoS ONE.

[127] Brunner D., Abramovitch A., Etherton J. (2017). A Yoga Program for Cognitive Enhancement. PLoS ONE.

[128] Welford P., Östh J., Hoy S., Rossell S.L., Pascoe M., Diwan V., Hallgren M. (2023). Effects of Yoga and Aerobic Exercise on Verbal Fluency in Physically Inactive Older Adults: Randomized Controlled Trial (FitForAge). Clin. Interv. Aging.

[129] Yoon D.H., Lee J.-Y., Song W. (2018). Effects of Resistance Exercise Training on Cognitive Function and Physical Performance in Cognitive Frailty: A Randomized Controlled Trial. J. Nutr. Health Aging.

[130] Mateluna-Núñez C., Ibáñez-Orellana R., Campos-Rojas C., Santana-Covarrubias A., Fuentes Figueroa R., Martínez-Flores R. (2025). The Effect of Free Weight Resistance Training on Cognitive Function Explored Through Eye Tracking: A Randomized Double-Blind Clinical Trial. Behav. Sci..

[131] Chiu C.N., Chen C.-Y., Muggleton N.G. (2017). Sport, Time Pressure, and Cognitive Performance. Progress in Brain Research.

[132] Schaefer S., Amico G. (2022). Table Tennis Expertise Influences Dual-Task Costs in Timed and Self-Initiated Tasks. Acta Psychol..

[133] Hola V., Polanska H., Jandova T., Jaklová Dytrtová J., Weinerova J., Steffl M., Kramperova V., Dadova K., Durkalec-Michalski K., Bartos A. (2024). The Effect of Two Somatic-Based Practices Dance and Martial Arts on Irisin, BDNF Levels and Cognitive and Physical Fitness in Older Adults: A Randomized Control Trial. Clin. Interv. Aging.

[134] Russo G., Bigliassi M., Ceciliani A., Tessari A. (2022). Exploring the Interplay between Sport Modality and Cognitive Function in Open- and Closed-Skill Athletes. Psychol. Sport Exerc..

[135] Leyland L.-A., Spencer B., Beale N., Jones T., Van Reekum C.M. (2019). The Effect of Cycling on Cognitive Function and Well-Being in Older Adults. PLoS ONE.

[136] Stenling A., Moylan A., Fulton E., Machado L. (2019). Effects of a Brief Stair-Climbing Intervention on Cognitive Performance and Mood States in Healthy Young Adults. Front. Psychol..

[137] Oberste M., De Waal P., Joisten N., Walzik D., Egbringhoff M., Javelle F., Bloch W., Zimmer P. (2021). Acute Aerobic Exercise to Recover from Mental Exhaustion—A Randomized Controlled Trial. Physiol. Behav..

[138] Raichlen D.A., Bharadwaj P.K., Nguyen L.A., Franchetti M.K., Zigman E.K., Solorio A.R., Alexander G.E. (2020). Effects of Simultaneous Cognitive and Aerobic Exercise Training on Dual-Task Walking Performance in Healthy Older Adults: Results from a Pilot Randomized Controlled Trial. BMC Geriatr..

[139] Feldberg C., Barreyro J.P., Quián M.D.R., Hermida P.D., Ofman S.D., Irrazabal N.C., Tartaglini M.F., Serrano C. (2024). Occupational Complexity of Paid Work and Housework, and Its Impact on the Cognitive Performance in Community Dwelling Older Adults, Preliminary Results. Dement. Neuropsychol..

[140] Lee S.Y., Pang B.W.J., Lau L.K., Jabbar K.A., Seah W.T., Chen K.K., Ng T.P., Wee S.-L. (2021). Cross-Sectional Associations of Housework with Cognitive, Physical and Sensorimotor Functions in Younger and Older Community-Dwelling Adults: The Yishun Study. BMJ Open.

[141] Wang N., Cai W., Pan X., Wang T., Gross A., Jiang C. (2025). Changes in Housework Frequency and Subsequent Cognitive Function and Rate of Decline Among Adults Aged ≥ 65 in the United States, 2008–2018. Perm. J..

[142] Craig C.L., Marshall A.L., Sjöström M., Bauman A.E., Booth M.L., Ainsworth B.E., Pratt M., Ekelund U., Yngve A., Sallis J.F. (2003). International Physical Activity Questionnaire: 12-Country Reliability and Validity. Med. Sci. Sports Exerc..

[143] Bull F.C., Maslin T.S., Armstrong T. (2009). Global Physical Activity Questionnaire (GPAQ): Nine Country Reliability and Validity Study. J. Phys. Act. Health.

[144] Robert H., Casillas J., Iskandar M., Dathis P., Antoine D., Taha S., Didier V., Scaglioni G., Caillaux B., Vanhoecke J. (2004). Le Score d’activité Physique de Dijon: Reproductibilité et Corrélations Avec l’aptitude Physique de Sujets Sains Âgés. Ann. Réadapt. Médecine Phys..

[145] Topolski T.D., LoGerfo J., Patrick D.L., Williams B., Walwick J., Patrick M.B. (2006). The Rapid Assessment of Physical Activity (RAPA) among Older Adults. Prev. Chronic. Dis..

[146] Washburn R.A., Smith K.W., Jette A.M., Janney C.A. (1993). The Physical Activity Scale for the Elderly (PASE): Development and Evaluation. J. Clin. Epidemiol..

[147] Stel V.S., Smit J.H., Pluijm S.M.F., Visser M., Deeg D.J.H., Lips P. (2004). Comparison of the LASA Physical Activity Questionnaire with a 7-Day Diary and Pedometer. J. Clin. Epidemiol..

[148] Chang Y.-H., Wu I.-C., Hsiung C.A. (2021). Reading Activity Prevents Long-Term Decline in Cognitive Function in Older People: Evidence from a 14-Year Longitudinal Study. Int. Psychogeriatr..

[149] Koyama M.S., Di Martino A., Zuo X.-N., Kelly C., Mennes M., Jutagir D.R., Castellanos F.X., Milham M.P. (2011). Resting-State Functional Connectivity Indexes Reading Competence in Children and Adults. J. Neurosci. Off. J. Soc. Neurosci..

[150] Guo L. (2023). The Delayed, Durable Effect of Expressive Writing on Depression, Anxiety and Stress: A Meta-Analytic Review of Studies with Long-Term Follow-Ups. Br. J. Clin. Psychol..

[151] Ferreira-Brito F., Ribeiro F., Aguiar De Sousa D., Costa J., Caneiras C., Carriço L., Verdelho A. (2021). Are Video Games Effective to Promote Cognition and Everyday Functional Capacity in Mild Cognitive Impairment/Dementia Patients? A Meta-Analysis of Randomized Controlled Trials. J. Alzheimer’s Dis..

[152] Zhang H., Peng Y., Li C., Lan H., Xing G., Chen Z., Zhang B. (2020). Playing Mahjong for 12 Weeks Improved Executive Function in Elderly People with Mild Cognitive Impairment: A Study of Implications for TBI-Induced Cognitive Deficits. Front. Neurol..

[153] Bediou B., Adams D.M., Mayer R.E., Tipton E., Green C.S., Bavelier D. (2018). Meta-Analysis of Action Video Game Impact on Perceptual, Attentional, and Cognitive Skills. Psychol. Bull..

[154] Li Z., Coretta S. (2026). Bilingualism Effect for Delaying Dementia Onset: A Bayesian Meta-Analysis. Neuropsychol. Dev. Cogn. B Aging Neuropsychol. Cogn..

[155] Berezuk C., Scott S.C., Black S.E., Zakzanis K.K. (2021). Cognitive Reserve, Cognition, and Real-World Functioning in MCI: A Systematic Review and Meta-Analysis. J. Clin. Exp. Neuropsychol..

[156] Fancourt D., Tymoszuk U. (2019). Cultural Engagement and Incident Depression in Older Adults: Evidence from the English Longitudinal Study of Ageing. Br. J. Psychiatry J. Ment. Sci..

[157] Fioranelli M., Roccia M.G., Garo M.L. (2023). The Role of Arts Engagement in Reducing Cognitive Decline and Improving Quality of Life in Healthy Older People: A Systematic Review. Front. Psychol..

[158] Liu S., Huang X., Liu Y., Yue J., Li Y., Chen L. (2024). A Scoping Review of the Use of Creative Activities in Stroke Rehabilitation. Clin. Rehabil..

[159] Colombo B., Antonietti A., Daneau B. (2018). The Relationships Between Cognitive Reserve and Creativity. A Study on American Aging Population. Front. Psychol..

[160] Fabio R.A., Bellantone A., Colombo B., Bertuccio D.V., Picciotto G. (2026). Cognitive Reserve and Creative Thinking in Aging: A Cross-Sectional Study on the Role of Education, Occupation, and Leisure Activities. J. Ageing Longev..

[161] Gassner L., Geretsegger M., Mayer-Ferbas J. (2022). Effectiveness of Music Therapy for Autism Spectrum Disorder, Dementia, Depression, Insomnia and Schizophrenia: Update of Systematic Reviews. Eur. J. Public Health.

[162] Walsh S., Causer R., Brayne C. (2021). Does Playing a Musical Instrument Reduce the Incidence of Cognitive Impairment and Dementia? A Systematic Review and Meta-Analysis. Aging Ment. Health.

[163] Wolff L., Quan Y., Perry G., Forde Thompson W. (2023). Music Engagement as a Source of Cognitive Reserve. Am. J. Alzheimer’s Dis. Dement..

[164] Demeco A., Bruno R.C., Bonfiglio R., Mancini L., Pisani F., Scozzafava L., Conte C., Ammendolia A., De Sire A., Marotta N. (2026). Effectiveness of Music Therapy with Personalized Rhythmic Auditory Stimulation Plus Music-Contingent Gait Training in Patients with Parkinson’s Disease: A Systematic Review. Neurol. Int..

[165] Trost W., Trevor C., Fernandez N., Steiner F., Frühholz S. (2024). Live Music Stimulates the Affective Brain and Emotionally Entrains Listeners in Real Time. Proc. Natl. Acad. Sci. USA.

[166] Shahaeian A., Wang C., Tucker-Drob E., Geiger V., Bus A.G., Harrison L.J. (2018). Early Shared Reading, Socioeconomic Status, and Children’s Cognitive and School Competencies: Six Years of Longitudinal Evidence. Sci. Stud. Read..

[167] Yaffe D., Shtoots L., Kochav Isakow O., Daniel Y., Reuveni O., Keisari S., Golland Y. (2025). Short Playful Interactions Improve Executive Functions in Children. Sci. Rep..

[168] Hua C., Peng S., Cole S. (2024). Travel and Mind: A Bidirectional Analysis of Travel Participation and Cognitive Functioning among Americans over 50. Work Aging Retire.

[169] Trotman H., Sakellariou D., Wood F., Hamana K. (2026). Mapping Social Participation Interventions for Adults with a Neurodegenerative Condition: A Scoping Review of in-Person and Digital Community Approaches. Disabil. Rehabil..

[170] Simon P., Cuervo-Lombard C.-V. (2025). Drama and Music Workshops for Elderly People with Cognitive Impairment: Mixed Analysis. Gériatrie Psychol. Neuropsychiatr. Vieil..

[171] Bourne P., Camic P.M., Crutch S.J. (2021). Psychosocial Outcomes of Dyadic Arts Interventions for People with a Dementia and Their Informal Caregivers: A Systematic Review. Health Soc. Care Community.

[172] Pruitt P.J., Lahna D., Schwartz D.L., Peltier S., Silbert L.C., Dodge H.H. (2022). Social Engagement Intervention Increases Dorsal Attention Network Functional Connectivity in Socially-isolated Older-old Adults: I-CONECT. Alzheimer’s Dement..

[173] Kreutz G., Bongard S., Rohrmann S., Hodapp V., Grebe D. (2004). Effects of Choir Singing or Listening on Secretory Immunoglobulin A, Cortisol, and Emotional State. J. Behav. Med..

[174] De Witte M., Spruit A., Van Hooren S., Moonen X., Stams G.-J. (2020). Effects of Music Interventions on Stress-Related Outcomes: A Systematic Review and Two Meta-Analyses. Health Psychol. Rev..

[175] Shahidullah A.H.M., Begum M. (2025). Evaluation of Stress Indicators among Stressful Young Individuals after Relaxing Music Intervention. Sch. J. Appl. Med. Sci..

[176] Cerasa A., Gaggioli A., Poggia G., Riva G. (2024). Metaverse in Mental Health: The Beginning of a Long History. Metaverse Ment. Health Begin. Long. Hist..

[177] García I., Martínez O., Amayra I., Salgueiro M., Rodríguez A.A., López-Paz J.F. (2024). Effects of a Neuropsychosocial Teleassistance Intervention on Social Cognition and Health-Related Quality of Life of Pediatric Patients with Neuromuscular Diseases. J. Pediatr. Psychol..

[178] Eisenhut H., Collett J., Yazdani F. (2024). Exploring the Barriers and Facilitators to Volunteering as an Intervention for Those with Long-term Neurological Conditions: How Make Therapeutic Volunteering Possible?. Health Expect..

[179] Huo M., Kim K. (2025). Volunteering with Mild Cognitive Impairment: Implications for Subsequent Cognitive Changes. Gerontol..

[180] Kelly M.E., Duff H., Kelly S., McHugh Power J.E., Brennan S., Lawlor B.A., Loughrey D.G. (2017). The Impact of Social Activities, Social Networks, Social Support and Social Relationships on the Cognitive Functioning of Healthy Older Adults: A Systematic Review. Syst. Rev..

[181] Ybarra O., Burnstein E., Winkielman P., Keller M.C., Manis M., Chan E., Rodriguez J. (2008). Mental Exercising Through Simple Socializing: Social Interaction Promotes General Cognitive Functioning. Pers. Soc. Psychol. Bull..

[182] Gelfo F., Petrosini L., Mandolesi L., Landolfo E., Caruso G., Balsamo F., Bonarota S., Bozzali M., Caltagirone C., Serra L. (2024). Land/Water Aerobic Activities: Two Sides of the Same Coin. A Comparative Analysis on the Effects in Cognition of Alzheimer’s Disease. J. Alzheimer’s Dis..

[183] Garrett J., Chak C., Bullock T., Giesbrecht B. (2024). A Systematic Review and Bayesian Meta-Analysis Provide Evidence for an Effect of Acute Physical Activity on Cognition in Young Adults. Commun. Psychol..

[184] Hogan C.L., Mata J., Carstensen L.L. (2013). Exercise Holds Immediate Benefits for Affect and Cognition in Younger and Older Adults. Psychol. Aging.

[185] Voelcker-Rehage C., Godde B., Staudinger U.M. (2011). Cardiovascular and Coordination Training Differentially Improve Cognitive Performance and Neural Processing in Older Adults. Front. Hum. Neurosci..

[186] Wicks C., Barton J., Orbell S., Andrews L. (2022). Psychological Benefits of Outdoor Physical Activity in Natural versus Urban Environments: A Systematic Review and Meta-analysis of Experimental Studies. Appl. Psychol. Health Well-Being.

[187] Yang L., Yuan Z., Peng C. (2025). Effects of Aerobic Exercise on Cognitive Function and Quality of Life in Patients with Alzheimer’s Disease: A Systematic Review and Meta-Analysis. BMJ Open.

[188] Decaix T., Bonnin C., Götze K., François V., Petit C., Rivière C., Greffard S., Cognat E., Hugon J., Paquet C. (2025). Benefits of Physical Activity on Cognitive Function in Patients with Neurocognitive Disorders: A Systematic Review. J. Frailty Aging.

[189] Aguirre-Loaiza H., Arenas J., Arias I., Franco-Jímenez A., Barbosa-Granados S., Ramos-Bermúdez S., Ayala-Zuluaga F., Núñez C., García-Mas A. (2019). Effect of Acute Physical Exercise on Executive Functions and Emotional Recognition: Analysis of Moderate to High Intensity in Young Adults. Front. Psychol..

[190] Elliott A.S., Moreno-Fernández R.D., Cordellat-Marzal A., Sampedro-Piquero P. (2024). Intensity of Physical Activity in Young People: Focus on Emotional, Cognitive, and Healthy Lifestyle-Related Variables. Behav. Sci..

[191] Krause-Sorio B., Siddarth P., Kilpatrick L., Milillo M.M., Aguilar-Faustino Y., Ercoli L., Narr K.L., Khalsa D.S., Lavretsky H. (2022). Yoga Prevents Gray Matter Atrophy in Women at Risk for Alzheimer’s Disease: A Randomized Controlled Trial. J. Alzheimer’s Dis..

[192] Thakur P., Mohammad A., Rastogi Y.R., Saini R.V., Saini A.K. (2020). Yoga as an Intervention to Manage Multiple Sclerosis Symptoms. J. Ayurveda Integr. Med..

[193] Eyre H.A., Siddarth P., Acevedo B., Van Dyk K., Paholpak P., Ercoli L., St. Cyr N., Yang H., Khalsa D.S., Lavretsky H. (2017). A Randomized Controlled Trial of Kundalini Yoga in Mild Cognitive Impairment. Int. Psychogeriatr..

[194] Brinsley J., Schuch F., Lederman O., Girard D., Smout M., Immink M.A., Stubbs B., Firth J., Davison K., Rosenbaum S. (2021). Effects of Yoga on Depressive Symptoms in People with Mental Disorders: A Systematic Review and Meta-Analysis. Br. J. Sports Med..

[195] Behere R.V., Arasappa R., Jagannathan A., Varambally S., Venkatasubramanian G., Thirthalli J., Subbakrishna D.K., Nagendra H.R., Gangadhar B.N. (2011). Effect of Yoga Therapy on Facial Emotion Recognition Deficits, Symptoms and Functioning in Patients with Schizophrenia. Acta Psychiatr. Scand..

[196] Sureka P., Govil S., Dash D., Dash C., Kumar M., Singhal V. (2014). Effect of Sudarshan Kriya on Male Prisoners with Non Psychotic Psychiatric Disorders: A Randomized Control Trial. Asian J. Psychiatry.

[197] Sharma V.K., Das S., Mondal S., Goswami U., Gandhi A. (2006). Effect of Sahaj Yoga on Neuro-Cognitive Functions in Patients Suffering from Major Depression. Indian J. Physiol. Pharmacol..

[198] Dupré C., Helmer C., Bongue B., Dartigues J.F., Roche F., Berr C., Carrière I. (2021). Associations between Physical Activity Types and Multi-Domain Cognitive Decline in Older Adults from the Three-City Cohort. PLoS ONE.

[199] Wenzel K.C., Van Puymbroeck M., Gagnon R., Lewis S., McGuire F., Vidotto J. (2024). The Attributes and Characteristics of Leisure Activity Engagement That Foster Cognition in Aging: A Scoping Review. J. Cogn. Enhanc..

